# Pitpnc1a Regulates Zebrafish Sleep and Wake Behavior through Modulation of Insulin-like Growth Factor Signaling

**DOI:** 10.1016/j.celrep.2018.07.012

**Published:** 2018-08-07

**Authors:** Tim G. Ashlin, Nicholas J. Blunsom, Marcus Ghosh, Shamshad Cockcroft, Jason Rihel

**Affiliations:** 1 Department of Neuroscience, Physiology and Pharmacology, University College London, London WC1E 6BT, UK; 2 Department of Cell and Developmental Biology, University College London, London WC1E 6BT, UK

**Keywords:** zebrafish, sleep, lipid transporter, behavior, IGF

## Abstract

The lipid transporters of the phosphatidylinositol transfer protein (PITP) family dictate phosphoinositide compartmentalization, and specific phosphoinositides play crucial roles in signaling cascades, membrane traffic, ion channel regulation, and actin dynamics. Although PITPs are enriched in the brain, their physiological functions in neuronal signaling pathways *in vivo* remain ill defined. We describe a CRISPR/Cas9-generated zebrafish mutant in a brain-specific, conserved class II PITP member, *pitpnc1a*. Zebrafish *pitpnc1a* mutants are healthy but display widespread aberrant neuronal activity and increased wakefulness across the day-night cycle. The loss of Pitpnc1a increases insulin-like growth factor (IGF) signaling in the brain, and inhibition of IGF pathways is sufficient to rescue both neuronal and behavioral hyperactivity in *pitpnc1a* mutants. We propose that Pitpnc1a-expressing neurons alter behavior via modification of neuro-modulatory IGF that acts on downstream wake-promoting circuits.

## Highlights

• Zebrafish have two orthologs, one brain enriched, of the lipid transporter PITPNC1
• CRISPR/Cas9-generated *pitpnc1a* mutants are hyperactive across the day-night cycle
• Insulin-like growth factor signaling is dysregulated in *pitpnc1a* mutants
• Blocking IGF signaling rescues *pitpnc1a* mutant behavioral and neuronal hyperactivity

## Graphical Abstract

**Figure:**
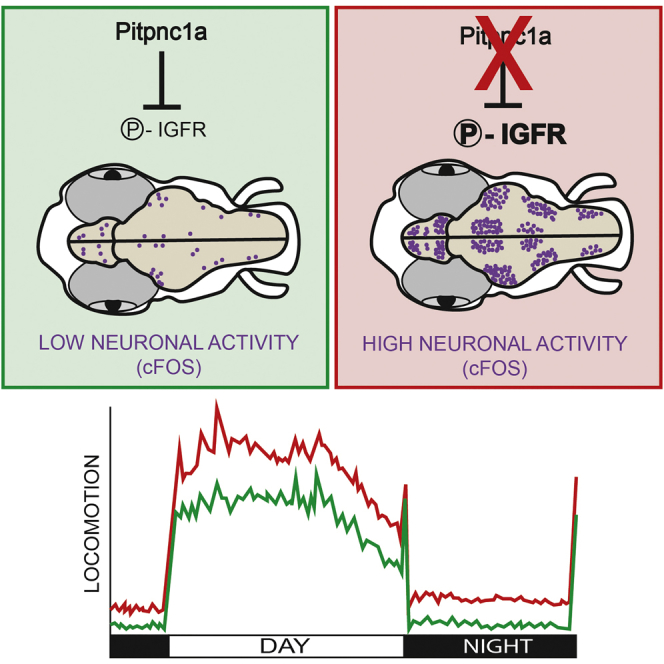

## Teaser

Ashlin et al. find that CRISPR/Cas9 zebrafish mutants that lack the brain-enriched lipid transporter Pitpnc1a have dysregulated insulin-like growth factor (IGF) signaling and behavioral hyperactivity. This work suggests that Pitpnc1a normally regulates the set point of neuronal excitability by dampening IGF.

## Introduction

Lipids are essential signaling molecules, with tight controls governing their levels and subcellular compartmentalization. One class of proteins with major roles in lipid regulation are the phosphatidylinositol transfer proteins (PITPs). PITPs bind and transfer phosphatidylinositol (PI), thereby regulating the levels of phosphoinositides, including PI(4,5)bisphosphate. PITPs have been especially implicated in neuronal lipid regulation to alter functions ranging from phototransduction to neurite outgrowth (Cockcroft, 2012). However, the diversity of PITPs means much is yet to be discovered about critical functions of PITPs in the brain.

The function of PITPNC1 in neurons has not been examined as extensively as it has for other PITP family members, such as RdgBα and PITPα. PITPNC1 has two isoforms generated via alternative splicing: the long variant PITPNC1-sp1 and the short variant PITPNC1-sp2 (Takano et al., 2003). These isoforms likely have distinct biochemical and functional properties, because only PITPNC1-sp1 can bind 14-3-3 proteins (Garner et al., 2011), and they are expressed in distinct regions of the embryonic mammalian brain (Takano et al., 2003). PITPNC1 has also been implicated in breast cancer cell metastasis: PITPNC1 expression is a key target of the metastasis-suppressing microRNA, miR-126 (Png et al., 2011); up to 46% of breast cancer tumors show amplified PITPNC1 expression; and an interaction between PITPNC1 and RAB1B at the Golgi was shown to drive malignant secretion of molecules that promote metastasis (Halberg et al., 2016). How PITPNC1’s role in pathogenic breast cancer relates to its endogenous function remains unknown.

To identify PITPNC1’s physiological function *in vivo*, we used CRISPR/Cas9 to create a zebrafish null mutant of a brain-specific ortholog of the human long isoform *pitpnc1a*. We find that *pitpnc1a* mutants have widespread, elevated neuronal activity and increased wakefulness across the 24 hr day-night cycle. Because insulin-like growth factor (IGF) signaling is upregulated in *pitpnc1a* mutants and inhibition of this pathway restores mutant behavior, we propose that Pitpnc1a modulates IGF signaling cascades to control the set point of neuronal excitability.

## Results

### A Zebrafish Pitpnc1 Ortholog Binds PI and PA

**Figure 1:**
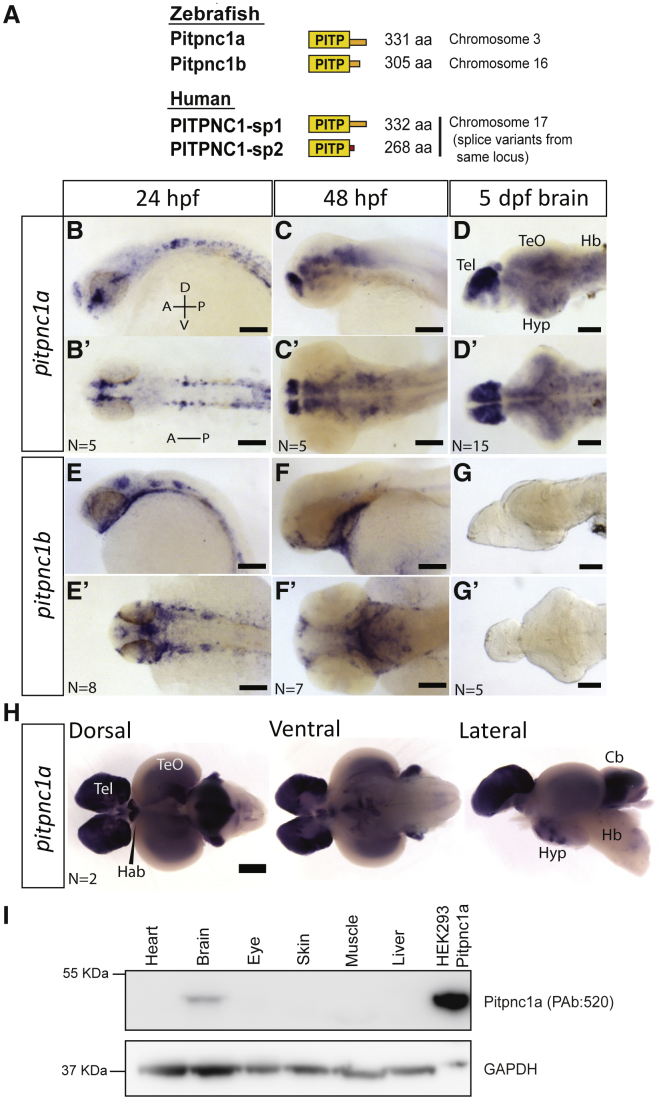
One Zebrafish PITPNC1 Ortholog Is Exclusively Expressed in the Brain (A) Zebrafish PITPNC1 orthologs and human splice variants. (B–D′) *pitpnc1a* mRNA is detected in the nervous system at 24 hpf (B and B′), 48 hpf (C and C′), and is widespread in the brain by 5 dpf (D and D′). (E–G′) *pitpnc1b* mRNA is expressed in non-neuronal tissue, including the pharyngeal arches and olfactory pits (E–F′). *pitpnc1b* mRNA is undetectable in the larval brain (G and G′). (H) *pitpnc1a* mRNA is expressed in many areas of the adult zebrafish brain. (I) Pitpnc1a protein is detected only in the adult zebrafish brain. Transfected HEK293 cells are a positive control. Tel, telencephalon; TeO, optic tectum; Hyp, hypothalamus; Hb, hindbrain; Hab, habenula; Cb, cerebellum. Scale bars, 100 μm (B–G′) and 500 μm (H).

To find zebrafish *pitpnc1*, we used the long human splice variant PITPNC1-sp1 (NP_036549.2) in zebrafish genome database queries (release GRCz10). Basic Local Alignment Search Tool – Protein (BLASTP) searches and expressed sequence tag (EST) alignments identified two zebrafish orthologs on chromosome 3 (renamed *pitpnc1a*), and chromosome 16 (renamed *pitpnc1b*) (Figure 1A; Figure S1A). The zebrafish *pitpnc1a* gene is predicted to encode a 331 amino acid protein that shows 81% identity (90% similarity) with the human long splice variant PITPNC1-sp1 (Figure S1B), while *pitpnc1b* encodes a shorter 305 amino acid protein similar to human PITPNC1-sp2 and only 57% identity (71% similarity) to human PITPNC1-sp1 (Figure S1B). Both zebrafish proteins contain the PITP family, inositol ring-coordinating amino acids (T59, K61, E86, and N90) (Tilley et al., 2004), but only Pitpnc1a retains potential 14-3-3 binding serine residues (Figure 1A; Figure S1B).

Using purified His- and FLAG-tagged Pitpnc1a protein, we tested whether zebrafish Pitpnc1a has biochemical properties similar to those of the human protein. Pitpnc1a-His was able to transfer both PI and PA (phosphatidic acid) *in vitro* at levels comparable to the human PITPNC1-sp1 isoform (Figures S2A and S2B). Immunoprecipitation of transfected, FLAG-tagged versions from Cos-7 cell lysates and probing for 14-3-3 proteins on a western blot revealed that both the human PITPNC1-sp1 and the zebrafish Pitpnc1a bound 14-3-3 proteins, whereas zebrafish Pitpnc1b did not (Figures S2C and S2D). Altogether, these data indicate that the zebrafish Pitpnc1a is capable of transferring both PI and PA and binding 14-3-3, similar to the human PITPNC1-sp1 protein.

### *pitpnc1a* Is Expressed in the Larval and Adult Zebrafish Brain

PITPNC1-sp1 has been detected in the adult mouse heart and brain, with specific enrichment in the dentate gyrus, thalamus, and Purkinje layer of the cerebellum (Garner et al., 2011, Takano et al., 2003). In zebrafish, we detected *pitpnc1a* transcripts by *in situ* hybridization (ISH) in several regions of the developing CNS by 24 hr post fertilization (hpf), including the dorsal forebrain, midbrain, and bilateral clusters of cells in the spinal cord (Figures 1B and 1B′). At 48 hpf, *pitpnc1a* expresses highly in the developing forebrain, midbrain, hypothalamus, and hindbrain (Figures 1C and 1C′). By larval stages (5 days post fertilization [dpf]), *pitpnc1a* expression is exclusively and extensively detected throughout the brain, with particularly strong expression in the dorsal telencephalon (Figures 1D and 1D′). *pitpnc1a* mRNA is also detectable in these areas in the adult zebrafish brain, with strong expression in the forebrain, habenula, and cerebellum, as well as expression in the optic tectum and several hypothalamic and hindbrain nuclei (Figure 1H). We did not detect *pitpnc1a* transcripts in the heart or other non-neuronal tissues. In contrast, *pitpnc1b* transcripts were excluded from the CNS at all stages, with the exception of some expression at 24 hpf around the developing brain ventricles that subsequently is undetectable (Figures 1E–1G′). Expression of *pitpnc1b* is localized to the pharyngeal arches and olfactory vesicles by 48 hpf (Figures 1F and 1F′), as well as in the pronephric duct (data not shown). At 5 dpf, there are no detectable *pitpnc1b* transcripts in the brain (Figures 1G and 1G′). The distinct tissue distribution of *pitpnc1a* and *pitpnc1b* supports the hypothesis that these genes have non-overlapping functions *in vivo*.

To detect Pitpnc1a protein, we developed a polyclonal antibody against the unique C terminus of zebrafish Pitpnc1a that shows no cross-reactivity with Pitpnc1b (Figure S2E). Western blot analysis detected Pitpnc1a protein only in the adult zebrafish brain, with no detectable protein in heart, liver, eye, skin, or muscle (Figure 1I). For comparison, a cross-reacting antibody against human PITPNC1-sp1 detects PITPNC1-sp1 protein in the adult rat heart and brain (Figure S2F). The restricted expression of Pitpnc1a allows functions of vertebrate PITPNC1 in the brain to be experimentally isolated *in vivo*.

### Zebrafish *pitpnc1a* Null Mutants Display Behavioral Hyperactivity

**Figure 2:**
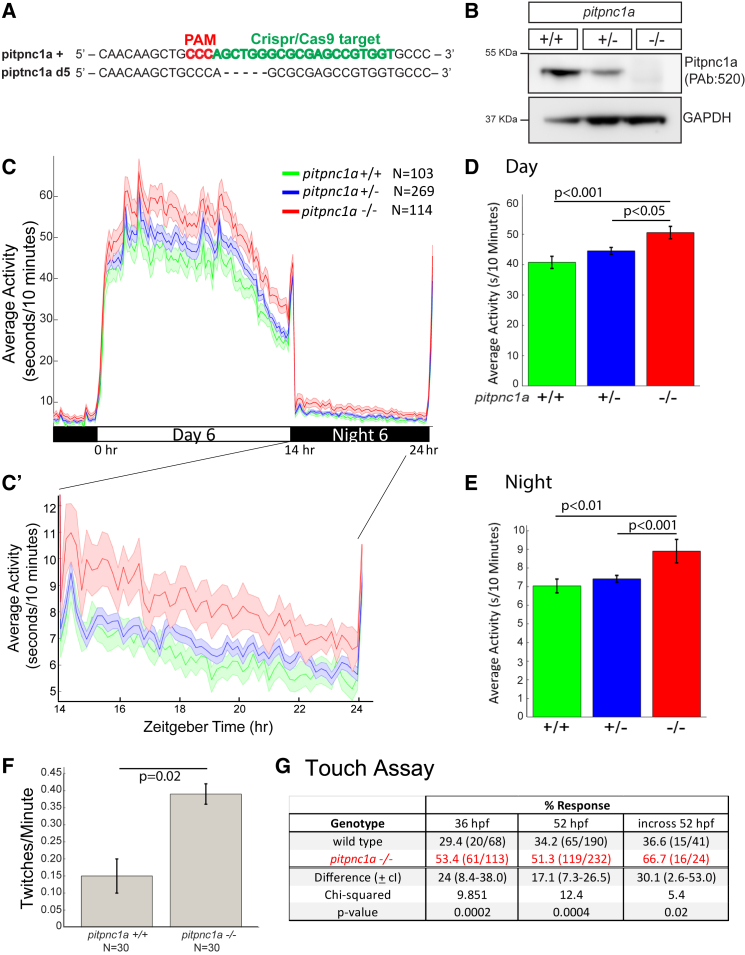
*pitpnc1a*^−/−^ Larvae Are Behaviorally Hyperactive (A) CRISPR/Cas9 generated a 5-base pair deletion in exon 2 of *pitpnc1a*. (B) Pitpnc1a protein is detected in *pitpnc1a*^+/+^ brain lysates, weaker in *pitpnc1a*^+/−^, and undetectable in *pitpnc1a*^−/−^. (C) An activity plot across a 14:10 hr day-night cycle (zoomed in C′) reveals hyperactivity in 6 dpf *pitpnc1a*^−/−^. The shaded ribbons represent ± SEM. (D and E) Bar plots (±SEM) of the mean day (D) and night (E) activity. *pitpnc1a*^−/−^ animals are significantly more active during both day and night (one-way ANOVA, Tukey’s post hoc test). (F) At 36 hpf, *pitpnc1a*^−/−^ embryos twitch significantly more than *pitpnc1a*^+/+^ (mean ± SEM, one-way ANOVA). (G) At both 36 and 52 hpf, *pitpnc1a*^−/−^ embryos are significantly more touch sensitive (pooled data from 5 and 4 independent experiments, respectively, and repeated in a blinded pitpnc1a^+/−^ in-cross; chi-square test).

To knock out Pitpnc1a function, we used CRISPR/Cas9 to introduce a 5-base pair deletion into exon 2 of *pitpnc1a* (Figure 2A) that is easily detected by high-resolution melt curve analysis (Figure S3C). This deletion introduces a frameshift to make a predicted truncated protein that lacks two key inositol binding residues (N90 and E86) (Figures S3A and S3B). Homozygous mutant *pitpnc1a*^Δ5/Δ5^ adult brains lack detectable Pitpnc1a protein by western blot (Figure 2B), indicating the Δ5 *pitpnc1a* allele is likely functionally null, and mutants will hereafter be called *pitpnc1a*^−/−^.

Larval and adult homozygous *pitpnc1a*^−/−^ mutants appear visually indistinguishable from wild-type siblings and are viable and fertile, and the Δ5 allele is inherited in Mendelian ratios (e.g., Figure 2C). Expression analysis of dorsal forebrain (*egr3*, *tbr1a*, and *eomesa*) and hypothalamic (*npvf*) markers found no changes in *pitpnc1a*^−/−^ larvae, suggesting that Pitpnc1a is not required for the specification or differentiation of these areas that strongly express *pitpnc1a* (Figures S3D and S3E).

Because Pitpnc1a is exclusively detected in the zebrafish brain, including areas that have been implicated in larval sleep and arousal (Barlow and Rihel, 2017), we asked whether behavior might be affected in *pitpnc1a*^−/−^ larvae by video-tracking them for several days (Rihel et al., 2010). Mutants showed a consistent increase in waking activity across a 14:10 hr light:dark cycle relative to *pitpnc1a*^+/+^ and *pitpnc1a*^+/−^ (Figures 2C–2E). Larval sleep during the day was also mildly reduced in both *pitpnc1a*^+/−^ and *pitpnc1a*^−/−^ larvae (Table S1). Because expression of *pitpnc1a* was detectable by 24 hpf, we also tested whether early embryonic behaviors were affected. Mutant embryos spontaneously coiled (i.e., twitched) significantly more often than wild-type controls at 36 hpf (Figure 2F) and were significantly more touch sensitive at both 36 and 52 hpf (Figure 2G). These results suggest that Pitpnc1a modulates the sensitivity of neurons involved in both stimulus-evoked and spontaneous behaviors.

### *pitpnc1a*^−/−^ Mutants Have Increased Neuronal Activity in Arousal-Related Circuits

**Figure 3:**
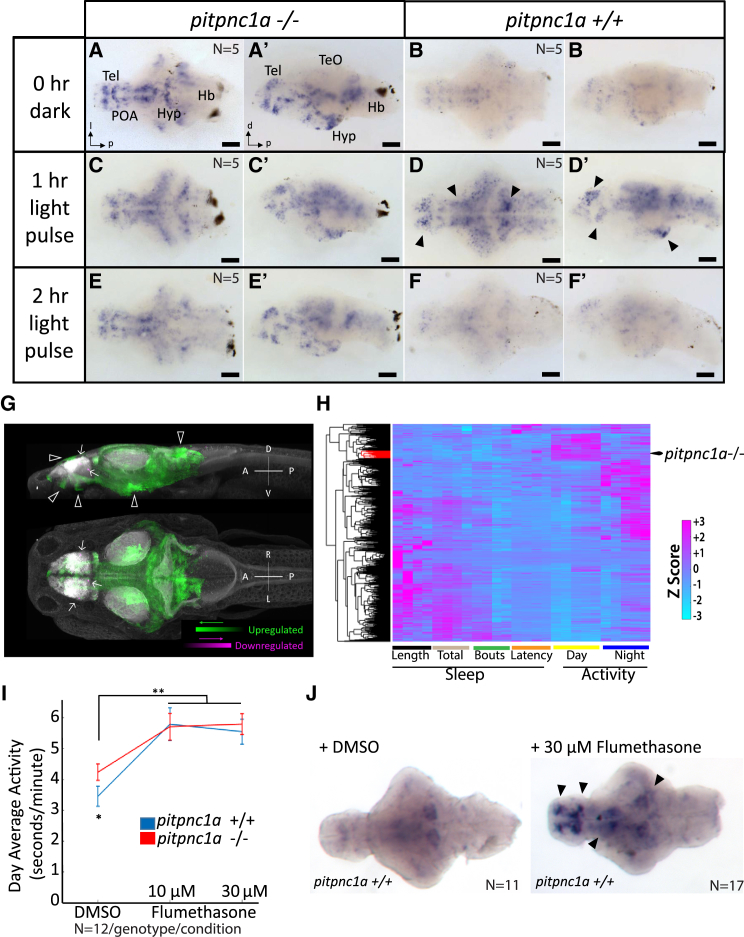
*pitpnc1a*^−/−^ Brains Have Increased Neuronal Activity (A–B′) ISH reveals many areas of *c-fos* expression in 6 dpf *pitpnc1a*^−/−^ larval brains (A and A′) compared with wild-type (B and B′). (C–D′) Following a 1 hr light pulse, additional neurons express *c-fos* in *pitpnc1a*^−/−^ (C and C′) and wild-type (D and D′). Many areas expressing *c-fos* in wild-type after a light pulse overlap with strong *c-fos* signals in *pitpnc1a*^−/−^ brains at 0 hr (black arrowheads, D and D′). (E–F′) After 2 hr of light, *c-fos* levels remain elevated in *pitpnc1a*^−/−^ brains (E and E′) but have returned to baseline in wild-type (F and F′). (G) pERK/tERK comparison of dark-reared, 6 dpf *pitpnc1a*^−/−^ (n = 15) and wild-type (n = 12) brains reveals mutant brain areas with upregulated (green) and downregulated (magenta) expression of pERK relative to controls. White arrowheads indicate areas of agreement between pERK and *c-fos* expression, and white arrows show the downregulated signal. Data are shown as a thresholded maximum projection overlaid on a maximum projection of the Z-Brain tERK reference (gray). (H) Clustering the behavioral fingerprint of *pitpnc1a*^−/−^ against 550 drug-induced behavioral profiles places the phenotype in a strong day-active and modestly night-active cluster (red branches). (I) Flumethasone increases the behavioral activity of both wild-type and *pitpnc1a*^−/−^ to the same level. DMSO-treated *pitpnc1a*^−/−^ animals are hyperactive (^∗^p < 0.05, one-way ANOVA, Tukey’s post hoc test). Both 10 and 30 μM flumethasone increase larval activity (^∗∗^p < 0.01, two-way ANOVA, gene × dose, Tukey’s post hoc test). (J) Flumethasone induces *c-fos* expression in areas similar to *pitpnc1a*^−/−^ and light pulses (black arrows). Hb, hindbrain; POA, preoptic area. (A–F) Dorsal view. (A′–F′) Lateral view. (J) Ventral view. Scale bars, 100 μm.

To map changes in neuronal activity that might underlie the mutant behavior, we raised zebrafish in constant darkness through 6 dpf, conditions that result in low expression of the immediate early gene, *c-fos*, in wild-type larvae (Figures 3B and 3B′). In contrast to dark-reared wild-type larvae, which have barely detectable *c-fos* expression, *pitpnc1a*^−/−^ mutants exhibited strong and extensive *c-fos* expression (Figures 3A and 3A′). The *c-fos* expression was particularly strong in a broad swath of the dorsal forebrain, two bilateral clusters in the ventral forebrain, the preoptic area, the midbrain, the posterior hypothalamus, and a bilateral cluster of neurons in the cerebellum (Figures 3A and 3A′). To determine whether these populations are similarly engaged during acute arousal, we exposed larvae to a light pulse. Following 1 hr light exposure, wild-type brains had increased *c-fos* expression in many areas that overlapped with those regions upregulated in *pipnc1a*^−/−^ larvae in the dark, including those in the dorsal and ventral forebrain, midbrain, and posterior hypothalamus (black arrowheads, Figures 3D and 3D′). Additional circuits involved in the detection and sensorimotor transformation of light also upregulated *c-fos* expression, including the optic tectum and hindbrain (Figures 3C and 3D). This indicates that the baseline *c-fos* expression in *pitpnc1a* mutants can be further elevated, at least in some neurons. After 2 hr of light exposure, *c-fos* levels have returned to near pre-pulse levels, with *pitpnc1a*^−/−^ maintaining higher expression than wild-type (Figures 3E and 3E′). Thus, many neuronal areas activated by light in wild-type are already active in *pitpnc1a*^−/−^ larvae in the dark, which is consistent with the increased mutant behavioral arousal during both day and night.

To complement the *c-fos* evidence, we used a measure of neuronal activity based on changes in the ratio of phosphorylated ERK (pERK) to total ERK (tERK), which has faster kinetics relative to *c-fos* transcription (Randlett et al., 2015). Relative to wild-type, *pitpnc1a*^−/−^ larvae have widespread neuronal activation, as detected by increased pERK/tERK ratio, with extensive overlap with areas observed by *c-fos*, including the dorsal and ventral forebrain, midbrain, and hypothalamus (Figure 3G; Figures S3F and S3G). However, pERK upregulation also extends to areas not observed by *c-fos* expression, including the optic tectum and hindbrain, which may reflect the faster kinetics or higher sensitivity of pERK relative to *c-fos*. Morphing this data onto the Z-Brain reference library identified brain regions and neuronal subtypes that are most strongly enriched for upregulated pERK activity in *pitpcn1a*^−/−^ brains (Table S2). This includes two dopaminergic populations of the diencephalon and the hypocretin neurons of the anterior hypothalamus (Figure 3G), which is consistent with an upregulation of known arousal circuits in the diencephalon. Other upregulated areas overlap with areas implicated in arousal, including the locus coeruleus and dorsal raphe (Table S2). In contrast, few mutant brain areas show a lower relative pERK signal (Figure 3G; Figures S3F and S3G; Table S2).

### Wake-Inducing Glucocorticoids Converge on Neurons Activated in *pitpnc1a* Mutants

If *pitpnc1a* mutants are hyperactive due to dysregulation of wake-promoting neurons, we speculated that wake-inducing drugs would activate the same neuronal populations. To identify likely drugs, we normalized the *pitpnc1a*^−/−^ behavioral parameters to wild-type to generate a behavioral fingerprint (Rihel et al., 2010), which was then hierarchically clustered against a dataset of 550 drug-induced behaviors (Figure 3H). The *pitpnc1a*^−/−^ cluster was enriched with anti-inflammatory compounds (46% of hits with a correlation coefficient > 0.7), including glucocorticoids (7 of the top 50), non-steroidal anti-inflammatory drugs (NSAIDs; 3/50), and phosphodiesterase inhibitors (6/50) (Table S3). This cluster is also enriched for NMDA antagonists (7/50). Thus, the *pitpnc1a*^−/−^ behavioral arousal converges onto drugs with shared biological properties that increase zebrafish wakefulness.

To test whether these drugs and the *pitpnc1a* mutation are altering behavior via common or parallel pathways, we examined *pitpnc1a* mutant and wild-type responses to the glucocorticoid flumethasone. As expected, flumethasone increased the waking activity of wild-type animals (Figure 3I). However, flumethasone only increased the waking activity of *pitpnc1a*^−/−^ larvae to the drug-treated wild-type level, indicating that *pitpnc1a*^−/−^ and flumethasone are non-additive and likely converge onto common neuronal circuits. Consistent with this interpretation, flumethasone upregulated *c-fos* expression in similar neuronal populations that are upregulated in *pitpnc1a*^−/−^ animals (Figure 3J).

### IGFR Signaling Is Upregulated in *pitpnc1a*^−/−^ Brains

**Figure 4:**
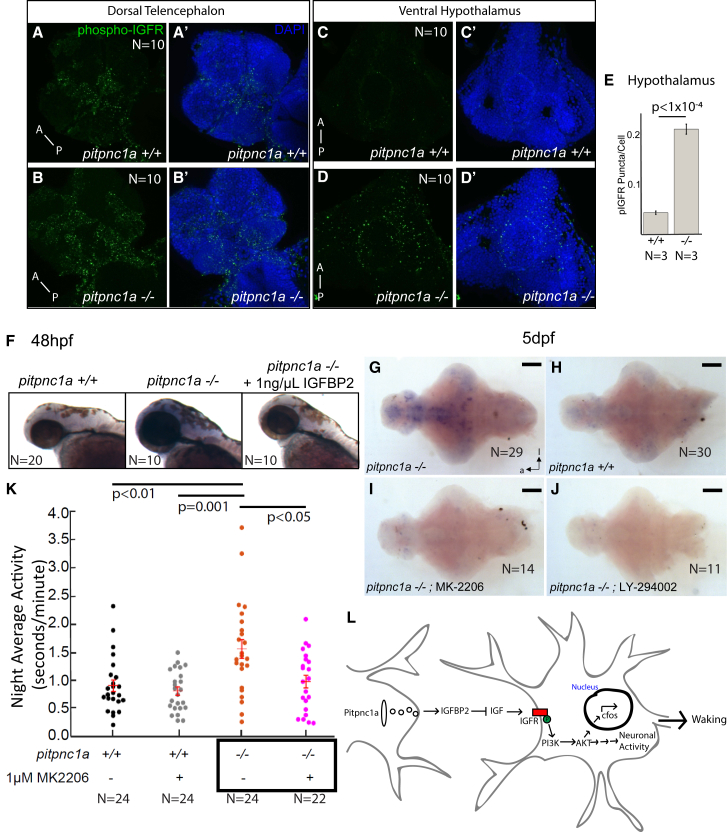
IGFR Signaling Is Enhanced in *pitpnc1a*^−/−^ Brains, and Inhibiting IGFR Signaling Rescues Neuronal and Behavioral Hyperactivity (A–E) 6 dpf *pitpnc1a*^−/−^ larvae have increased pIGFR puncta in the brain. (A–B′) Confocal projections of the dorsal telencephalon in wild-type (A and A′) and *pitpnc1a*^−/−^ (B and B′) reveal pIGFR puncta (green). Nuclei are DAPI stained (blue) in (A′) and (B′). Confocal projections of the *pitpnc1a*^−/−^ ventral hypothalamus (D and D′) have more pIGFR puncta (C and C′), as quantified in (E) (mean ± SEM, one-way ANOVA). (F) Upregulated *c-fos* in *pitpnc1a*^−/−^ embryos is reduced to wild-type by soaking in 1 ng/μL IGFBP2 from 36 to 48 hpf. (G–J) ISH for *c-fos* in 6 dpf brains in constant dark after overnight exposure to PI3K (LY-294002, 2 μM) or Akt (MK-2206, 100 nM) inhibitors. (G and H) *c-fos* is upregulated in *pitpnc1a*^−/−^ brains (G) relative to WT controls (H). (I and J) Akt (I) and PI3K (J) inhibitors reduce mutant *c-fos* expression to wild-type levels. Brains in (G)–(J) are overdeveloped to expose even weak *c-fos* expression. (K) Exposure to 1 μM Akt inhibitor, MK-2206, reduces average night-time activity of 6 dpf *pitpnc1a*^−/−^ larvae to wild-type levels. Each dot represents a single larva, and the crossbars plot the mean ± SEM (two-way ANOVA, genotype × drug interaction, Tukey’s post hoc test). (L) Model of Pitpnc1a modulation of neuronal activity via IGFBP2 and inhibition of IGF-PI3K-Akt. Loss of Pitpnc1a enhances IGF signaling, leading to increased waking behavior. (G–J) Ventral view; scale bars, 100 μm. (A–B′) Scale bars, 100 μm. (C–E′) Scale bars, 20 μm.

Because PITPNC1 enhances the secretion of pro-metastatic factors and insulin growth factor binding protein 2 (IGFBP2) (Halberg et al., 2016, Png et al., 2011), we tested whether IGF signaling was altered in *pitpnc1a*^−/−^ brains. We used antibodies against IGF-1 receptor beta phosphorylated at Y1135 (hereafter called pIGFR) to detect activation of the IGF pathway. We detected several pIGFR puncta in the dorsal telencephalon of 6 dpf wild-type brain that were even more extensive in *pitpnc1a*^−/−^ brains (Figures 4A and 4B). In the ventral hypothalamus, significantly fewer pIGFR puncta were detected in wild-type brains compared to mutants (Figures 4C–4E). In contrast, in the trunk and tail, which lack Pitpnc1a, pIGFR levels were unaffected (Figures S4A and S4B). To confirm that these puncta represent bona fide pIGFR, we soaked wild-type and mutant larvae in IGFBP2, which abolished nearly all pIGFR puncta (Figure S4C). If IGF signaling is upregulated in the *pitpnc1a*^−/−^ brains, IGF should stimulate increased *pitpnc1a*^−/−^ brain growth (Duan et al., 1999). We found that *pitpnc1a* mutants had a significant increase in the ratio of intra-ocular brain width to body length and a trend toward an increased brain length (Figures S4E–S4G). Thus, *piptnc1a* mutants have increased insulin-like growth factor receptor (IGFR) signaling and an increase in brain growth.

### Inhibition of IGF Signaling Rescues *pitpnc1a*^−/−^ Neuronal Activity and Behavior

To test whether increased IGF signaling leads to altered mutant behavior, we first soaked larvae overnight from 36 to 48 hpf in 1 ng/μL recombinant human IGFBP2 and assessed neuronal activity by *c-fos* expression. Similar to 5 dpf brains, untreated *pitpnc1a* mutants had elevated *c-fos* levels, but overnight exposure to IGFBP2 reduced mutant *c-fos* back to wild-type levels (Figure 4F; Figure S4D).

We next tested whether inhibition of phosphatidylinositol 3-kinase (PI3K) and Akt, components downstream of IGFR signaling, could affect the aberrant neural and behavioral hyperactivity in *pitpnc1a* mutants. Overnight (>16 hr) soaking of *pitpnc1a*^−/−^ larvae in either the PI3K inhibitor LY294002 (2 μM) or the allosteric Akt inhibitor MK-2206 (100 nM) rescued *c-fos* staining from strong upregulation in mutants (Figures 4G and 4H) to wild-type levels (Figures 4I and 4J). Soaking *pitpnc1a*^−/−^ larvae in 1 μM MK-2206 also resulted in a reversion of the mutant night-time hyperactivity to wild-type levels (Figure 4K), suggesting that upregulated IGFR signaling in *pitpnc1a* mutants leads to aberrant neuronal and behavioral hyperactivity.

## Discussion

We found that the brain-specific, class II lipid transfer protein Pitpnc1a is essential for maintaining proper neuronal activity and wakefulness via modulation of IGF signaling. Our work not only has implications for understanding the impact of phospholipids on behavior but also may have clinical relevance, because PITPNC1 is upregulated in many metastatic tumors (Halberg et al., 2016) and polymorphisms in PITPNC1 have been linked to type 2 diabetes mellitus (Greenawalt et al., 2012, Liu et al., 2017).

### Pitpnc1a Modulates Baseline Behavioral Activity

Zebrafish have two highly conserved and differentially expressed orthologs of the human PITPNC1 gene, *pitpnc1a* and *pitpnc1b*, which correspond to the long and short human isoforms, respectively. Evolution often redistributes a protein’s expression and function across duplicated genes, or ohnologs (Pasquier et al., 2016), and this feature allowed us to isolate the role of Pitpnc1 proteins in the brain by targeting zebrafish *pitpnc1a*.

CRISPR/Cas9-generated *pitpnc1a* mutants are hyperactive across the day:night cycle and exhibit increased neuronal activity as measured by both *c-fos* and pERK. Many of the neuronal populations that are most strongly upregulated in *pitpnc1a*^−/−^ larvae overlap with neurons implicated in setting levels of zebrafish wakefulness, including arousing hypothalamic and hindbrain populations. In addition, the non-additivity and neuronal convergence between wake-promoting glucocorticoids and *pitpnc1a* mutants indicates that neurons of the ascending arousal system may act in concert to affect *pitpnc1a* mutant hyperactivity. The human PITPNC1 gene resides within a copy number variant associated with a syndromic intellectual disability caused by loss of the neighboring gene PSMD12 (Küry et al., 2017). Individuals that have lost a larger region encompassing PITPNC1 often exhibit hyperactivity.

### Pitpnc1a, IGF, and Neuronal Activity

The *pitpnc1a* mutants had increased activation of IGF signaling, and blocking this cascade upstream with IGFBP2 or downstream with PI3K and Akt inhibitors was sufficient to restore neuronal and behavioral activity. Taking this data together with observations in the cancer literature, we propose a model (Figure 4L) in which Pitpnc1a stimulates the secretion of inhibitory IGFBP2 that acts as an IGF counterbalance onto circuits involved in wakefulness. When Pitpnc1a is missing, IGF signaling is too high, leading to PI3K-Akt activation, upregulation of neuronal activity, and ultimately increased wakefulness.

Altered PI3K-Akt signaling has been implicated in a variety of neurodevelopmental and psychiatric diseases, including autism (Chen et al., 2014) and schizophrenia (Zheng et al., 2012), but the mechanisms by which this may affect neuronal circuits remains obscure. In light of the data that Pitpnc1a modulates IGF-PI3K-Akt signaling in the vertebrate brain, the role of lipid transporters in the progression, mitigation, or exacerbation of these complex mental disorders should be further investigated.

## Experimental Procedures

### Zebrafish Strains

AB/TL zebrafish were maintained at 28.5°C by the University College London (UCL) Fish Facility. *pitpnc1a*^−/−^ animals were generated using CRISPR/Cas9 and outcrossed back to AB/TL for at least 3 generations. Work was in accordance with the Animal Experimental Procedure Act (1986) under license 70/7612.

### CRISPR/Cas9 Targeting *pitpnc1a* and Genotyping

CRISPR/Cas9 targeting of *pitpnc1a* exon 2 was carried out as described (Hwang et al., 2013), using the guide RNA sequence (5′-ACCACGGCTCGCGCCCAGCT-3′), which was synthesized as a DNA template using GeneArt Strings (Invitrogen) and then transcribed with a T7 RNA synthesis kit (New England Biolabs). PCS2-Cas9 (Addgene 47322), a gift from Alex Schier, was transcribed using a SP6 mMESSAGE mMACHINE kit (Ambion) and then co-injected with the guide RNA (gRNA) into one-cell embryos. Genomic DNA was Hotshot extracted from single 24 hpf embryos; 85 base pair flanking the cut site were PCR amplified (forward: 5′-TCTGTCCGTCTGCTCTCTTC-3′; reverse: 5′-AGGCTTTCTCCGTCACGTAG-3′) and subjected to high resolution melt curve analysis (HRMA) using Precision Melt Supermix (Bio-Rad). Mutations were confirmed by Sanger sequencing (Source Biosciences).

### pERK/tERK Activity Mapping

#### Immunohistochemistry

Fish were fixed overnight at 4°C in 4% paraformaldehyde (PFA) and 4% sucrose in PBS; permeabilized 45 min in 0.05% trypsin-EDTA on ice; blocked 6 hr at room temperature (RT) in phosphate buffered saline plus 0.05% Triton (PBT) plus 2% normal goat serum, 1% BSA, and 1% DMSO; and then incubated over sequential nights at 4°C in primary antibodies (Cell Signaling Technology 4370 and 4696; 1:500) and secondary antibodies conjugated with Alexa fluorophores (Life Technologies; 1:200) in PBT plus 1% BSA and 1% DMSO.

#### Imaging

Larvae were mounted in 1.5% low melt agarose and imaged with a custom two-photon microscope (Bruker; Prairie View software) with a 20× water immersion objective (Olympus).

#### Z-Brain Registration and Mapping

Images were noise filtered using a custom MATLAB (The MathWorks) scripts and registered into Z-Brain using the Computational Morphometry Toolkit (http://www.nitrc.org/projects/cmtk/) with the command string: -a -w -r 0102 -l af -X 52 -C 8 -G 80 -R 3 -A "--accuracy 0.4 --auto-multi-levels 4" -W "--accuracy 1.6" -T 4. Registered images were prepared using a custom MATLAB/MIJ (http://bigwww.epfl.ch/sage/soft/mij/) script to downsize, blur, and adjust the maximum brightness of each stack to the top 0.1% of pixel intensities to preserve dynamic range. Activity maps were generated using MATLAB scripts (Randlett et al., 2015).

### Behavioral Analysis

#### Sleep and Wake

Larvae were raised on a 14:10 hr light:dark cycle at 28.5°C. On 4 dpf, larvae were singly placed into a clear 96-square well plate (Whatman) filled with 650 μL of fish water (0.3 g/L Instant Ocean and 1 mg/L methylene blue [pH 7.0]). The plate was placed in a custom-modified Zebrabox (ViewPoint Life Sciences), and each well was monitored by automated tracking software and analyzed as in Rihel et al. (2010).

#### Spontaneous Coiling

Wild-type and mutant embryos from 30–36 hpf were kept in their chorion and placed in rows of 10 under a dissecting microscope. Coils per embryo were manually counted in three independent experiments.

#### Touch Sensitivity

36 and 52 hpf mutant and wild-type larvae were singly pipetted into a dish and then touched on the tail or head with a bent pipet tip. A response was recorded if the larva elicited an escape. One set was performed on larvae from a *pitpnc1a*^+/−^ in-cross followed by genotyping.

### *In Vivo* Drug Experiments

Larvae were dark reared at 28.5°C to 5 dpf and then exposed to the PI3K inhibitor LY294002 or the Akt inhibitor MK-2206 in 5 mL of water in six-well plates (n = 25) overnight, anesthetized with MS-222, and fixed with 4% PFA. For behavioral testing, drugs dissolved in DMSO were added to each well (final concentrations of 100 nM to 10 μM, 0.2% DMSO). Outliers (n = 1) were excluded by Grubb’s test (p < 0.01). For IGFBP2 soaking, 10 dechorionated 34 hpf embryos were transferred into 100 μL of fish water with 0, 0.05, 0.1, 0.5, and 1 ng/μL of recombinant human IGFBP2 (PeproTech, Catalog no. 350-06B) in a 96-well plate overnight at 28.5°C.

### Statistical Analysis

Behavioral data were analyzed for statistical significance using one-way ANOVA followed by Tukey’s post hoc test (α = 0.05). Interactions between genotype and drug were analyzed by two-way ANOVA (α = 0.05). Touch sensitivity was analyzed by chi-square test.

### Molecular and Biochemical Methods

See Supplemental Information for additional information.

## Supplemental Information

Document S1. Supplemental Experimental Procedures, Figures S1–S4, and Tables S1–S3

Document S2. Article plus Supplemental Information
