# Supplementary material for: Pitpnc1a Regulates Zebrafish Sleep and Wake Behavior through Modulation of Insulin-like Growth Factor Signaling
Source: Cell Rep. 2018 Aug 7;24(6):1389–96. doi: 10.1016/j.celrep.2018.07.012 (PMC6092267; doi:10.1016/j.celrep.2018.07.012)
Supplement: Document S1. Supplemental Experimental Procedures, Figures S1–S4, and Tables S1–S3 [file mmc1.pdf]

Cell Reports, Volume *24*

Supplemental Information

Pitpnc1a Regulates Zebrafish Sleep and Wake Behavior through Modulation of Insulin-like Growth Factor Signaling

Tim G. Ashlin, Nicholas J. Blunsom, Marcus Ghosh, Shamshad Cockcroft, and Jason Rihel

<u>Supplementary Materials</u>

Figure S1-related to Figure 1. Zebrafish have two orthologs of human PITPNC1

Figure S2-related to Figure 1. Zebrafish Pitpnc1a shares biochemical properties with human PITPNC1.

Figure S3-related to Figure 2. A CRISPR/Cas9 generated five base deletion of zebrafish *pitpnc1a* leads to a truncated protein lacking key functional residues.

Figure S4-related to Figure 4. IGFBP2 inhibits IGFR, *c-fos* expression, and brain size in *pitpnc1a*$^{-/-}$ animals.

Supplementary Table S1-related to Figure 2. Behavioral parameters of *pitpnc1a*$^{-/-}$ larvae.

Supplementary Table S2-related to Figure 3. Anti-inflammatory compounds with behavioral fingerprints that co-cluster with *pitpnc1a*$^{-/-}$ larvae.

Supplementary Table S3-related to Figure 3. MAP-Mapped brain regions and transgenic lines that overlap with up- and down- regulated pERK signals in *pitpnc1a*$^{-/-}$ larvae.

[A]

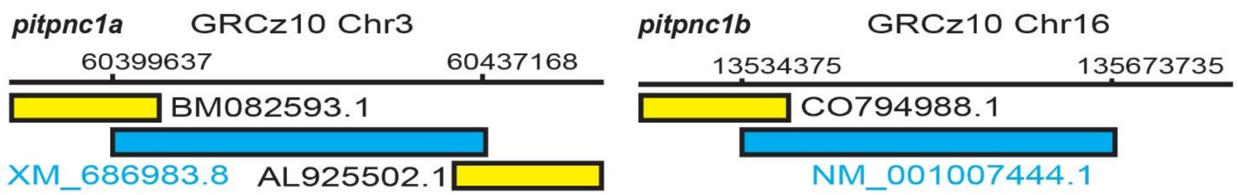

[B]

```
Pitpnc1a_Dr        1 MLMKEYRICMPLTVEEYRIGQLYMISKHSHEQSERGEGVEVVQNEPYDDPNYGSGQFTEK
PITPNC1-sp1_Hs     1 MLLKEYRICMPLTVDEYKIGQLYMISKHSHEQSDRGEGVEVVQNEPFEDPHHGNGQFTEK
Pitpnc1b_Dr        1 MLVKEYRICMPLTVDEYRIGQLYMISKHSNEQSGGGEGVEVVRNEPCTDPKHGSGQITEK
PITPNC1-sp2_Hs     1 MLLKEYRICMPLTVDEYKIGQLYMISKHSHEQSDRGEGVEVVQNEPFEDPHHGNGQFTEK

Pitpnc1a_Dr       61 RIYLNNKLPSWARAVVPKIFYVTEKAWNYYPYTITEYTCSFLPKFSIHIETKYEDNKGVN
PITPNC1-sp1_Hs    61 RVYLNSKLPSWARAVVPKIFYVTEKAWNYYPYTITEYTCSFLPKFSIHIETKYEDNKGSN
Pitpnc1b_Dr       61 RIYLNSKLPTWMRRFVPMVFYLTETAWNFYPYTITEYTCSFLPRFHVKIETHFKNDNGCS
PITPNC1-sp2_Hs    61 RVYLNSKLPSWARAVVPKIFYVTEKAWNYYPYTITEYTCSFLPKFSIHIETKYEDNKGSN

Pitpnc1a_Dr      121 DHIFDTELRDEETEVCIVDIAYDEIPERYYKESEDPRQFKSQKTSRGMLKEGWRDTQDPI
PITPNC1-sp1_Hs   121 DTIFDNEAKDVEREVCFIDIACDEIPERYYKESEDPKHFKSEKTGRGQLREGWRDSHQPI
Pitpnc1b_Dr      121 KNVFGDDPTPMD-NVCFLDILSDPIPEKNYKKSEDLSDWVSEKTGRGPLVDGWRNDTKPI
PITPNC1-sp2_Hs   121 DTIFDNEAKDVEREVCFIDIACDEIPERYYKESEDPKHFKSEKTGRGQLREGWRDSHQPI

Pitpnc1a_Dr      181 MCSYKLVTVKFEVWGLQTRVEQFVHKVVRDVLLLGHRQAFAWVDEWIDMTMEEVREYERA
PITPNC1-sp1_Hs   181 MCSYKLVTVKFEVWGLQTRVEQFVHKVVRDILLIGHRQAFAWVDEWYDMTMDEVREFERA
Pitpnc1b_Dr      180 MCSYKRVTCSFEVYGFQGRTEDFVHRNIRDILLVGHRQAVAWIDEWHGMTLEQVREFERK
PITPNC1-sp2_Hs   181 MCSYKLVTVKFEVWGLQTRVEQFVHKVVRDILLIGHRQAFAWVDEWYDMTMDDVREYEKN

Pitpnc1a_Dr      241 TQEATNKKLGTFPPAIAISETP-LPACARSGPSSAPSTPLSTEAPDFLSVPKDRPRKKSA
PITPNC1-sp1_Hs   241 TQEATNKKIGIFPPAISISSIPLLPSSVRSAPSSAPSTPLSTDAPEFLSVPKDRPRKKSA
Pitpnc1b_Dr      240 QQEETNCKLKSDI----------------ASPGSDPAIRP--SFTRSISVTDESSLKKMG
PITPNC1-sp2_Hs   241 MHEQTNIKVCNQH----------------SSPVDDIESHA--------------------

Pitpnc1a_Dr      300 PETLTLPDPARRDSAFRLPSLFSWG-SSSP--QPE
PITPNC1-sp1_Hs   301 PETLTLPDPEK-KATLNLPGMHSSDKPCRP--KSE
Pitpnc1b_Dr      282 VTTLDIPSS---QSSLKGP--------VRLKSTPE
PITPNC1-sp2_Hs   265 ------------Q-TST------------------
```

Figure S1-related to Figure 1

Figure S1. Zebrafish have two orthologs of human PITPNC1, related to Figure 1 A) A schematic showing the EST sequences that were used to assemble *pitpnc1a* and *pitpnc1b* genes in the absence of annotated sequences. The EST XM_686983.8 was missing part of the expected 5' and 3' ends of the *pitpnc1a* transcript, including the expected start and stop codons. Alignments revealed partial ESTs BM082593.1 and AL925502.1 overlapped with the partial transcript and contained the full length ORF. The complete *pitpnc1b* sequence was assembled from the two ESTs CO794988.1 and NM_001007444.1. B) Alignments of zebrafish Pitpnc1a and Pitpnc1b with the human PITPNC1-sp1 and PITPNC1-sp2 protein sequences. The black background indicates amino acid residues that are identical and gray background indicates amino acid similarity. Key residues important for binding of the inositol ring of phosphatidylinositol (T59, K61, E86, and N90, mouse numbering) are highlighted in red and the phosphorylation sites that are important for 14-3-3 binding are highlighted in green.

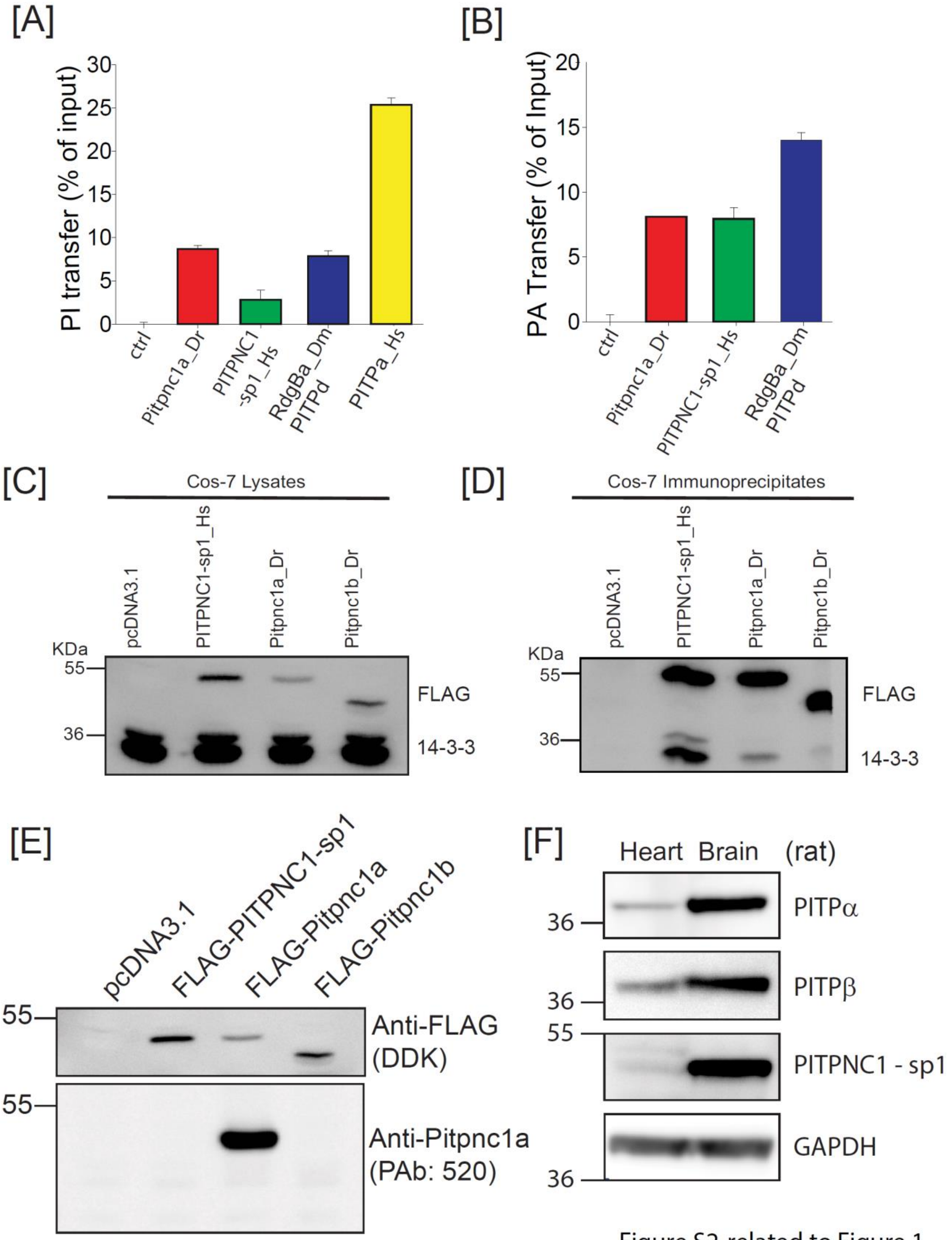

Figure S2-related to Figure 1

Figure S2. Zebrafish Pitpnc1a shares biochemical properties with human PITPNC1, related to Figure 1. A-B) Purified recombinant His-tagged zebrafish Pitpnc1a transfers both PI (A) and PA (B) *in vitro*, similar to human PITPNC1. The data is from a representative experiment done in duplicate; the error bars represent the range. C-D) FLAG-tagged PITPNC1-sp1_Hs, Pitpnc1a_Dr and Pitpnc1b_Dr constructs were electroporated into Cos-7 cells, immunoprecipiated against the FLAG-tags, and simultaneously probed by Western blot with antibodies against the Flag-tag and 14-3-3. Recombinant proteins and 14-3-3 (The 14-3-3 protein family comprises of 28-33 kDa proteins and the antibody detects all isoforms) were detectable in all lysates (C), but only the long form PITPNC1_Hs and Pitpnc1a_Dr were able to pull down 14-3-3 proteins (D). E) Flag-tagged Pitpnc1a and Pitpnc1b were expressed in Cos-7 cells by electroporation. Western blotting with a monoclonal anti-FLAG antibody serves as a positive control (top panel). The antibody recognizes only zebrafish Pitpnc1a. F) Western blot of the cytosolic fractions of rat brain and heart tissues probed with a cross-reacting anti-PITPNC1-sp1 antibody (RB59, see methods) strongly detects PITPNC1-sp1 in the rat brain and weakly in the heart. Antibodies to PITPα (Ab:674) and PITPβ (4A7) also detect these proteins in rat brain and heart.

[A]

```
pitpnc1a-delta5_Dr/1-336     1 ATGTTGATGAAGGAATACCGGATATGCATGCCGCTGACCGTGGAGGAGTA  50
pitpnc1a-delta5_Dr/1-336    51 CAGGATTGGTCAGCTGTACATGATCAGCAAACACAGTCATGAGCAGAGCG 100
pitpnc1a-delta5_Dr/1-336   101 AGAGAGGAGAGGGTGTGGAGGTGGTGCAAAACGAACCCTACGATGACCCA 150
pitpnc1a-delta5_Dr/1-336   151 AACTACGGCTCTGGACAGTTCACAGAGAAGCGCATTTATCTCAACAACAA 200
pitpnc1a-delta5_Dr/1-336   201 GCTGCCCAGCGCGAGCCGTGGTGCCCAAAATCTTCTACGTGACGGAGAAA 250
pitpnc1a-delta5_Dr/1-336   251 GCCTGGAATTACTATCCTTACACCATCACAGAGTATACATGCTCTTTCCT 300
pitpnc1a-delta5_Dr/1-336   301 GCCAAAGTTCTCCATCCACATCGAGACAAAGTATGA               336
```

[B]

```
Pitpnc1a_Dr/1-331            1 MLMKEYRICMPLTVEEYRIGQLYMISKHSHEQSERGEGVEVVQNEPYDDPN  51
Pitpnc1a-delta5_Dr/1-111     1 MLMKEYRICMPLTVEEYRIGQLYMISKHSHEQSERGEGVEVVQNEPYDDPN  51

Pitpnc1a_Dr/1-331           52 YGSGQFTEKRIYLNNKLPSWARAVVPKIFYVTEKAWNYYPYTITEYTCSFL 102
Pitpnc1a-delta5_Dr/1-111    52 YGSGQFTEKRIYLNNKLPSASRG----------------------------  74

Pitpnc1a_Dr/1-331          103 PKFSIHIETKYEDNKGVNDHIFDTELRDEETEVCIVDIAYDEIPERYYKES 153
Pitpnc1a-delta5_Dr/1-111       ---------------------------------------------------

Pitpnc1a_Dr/1-331          154 EDPRQFKSQKTSRGMLKEGWRDTQDPIMCSYKLVTVKFEVWGLQTRVEQFV 204
Pitpnc1a-delta5_Dr/1-111    75 -----------AQNLLRDG--------------------ESLELLSLHHHRV  95

Pitpnc1a_Dr/1-331          205 HKVVRDVLLLGHRQAFAWVDEWIDMTMEEVREYERATQEATNKKLGTFPPA 255
Pitpnc1a-delta5_Dr/1-111    96 YMLFPAKVLHPHRDKV----------------------------------- 111

Pitpnc1a_Dr/1-331          256 IAISETPLPACARSGPSSAPSTPLSTEAPDFLSVPKDRPRKKSAPETLTLP 306
Pitpnc1a-delta5_Dr/1-111       ---------------------------------------------------

Pitpnc1a_Dr/1-331          307 DPARRDSAFRLPSLFSWGSSSPQPE                           331
Pitpnc1a-delta5_Dr/1-111       -------------------------
```

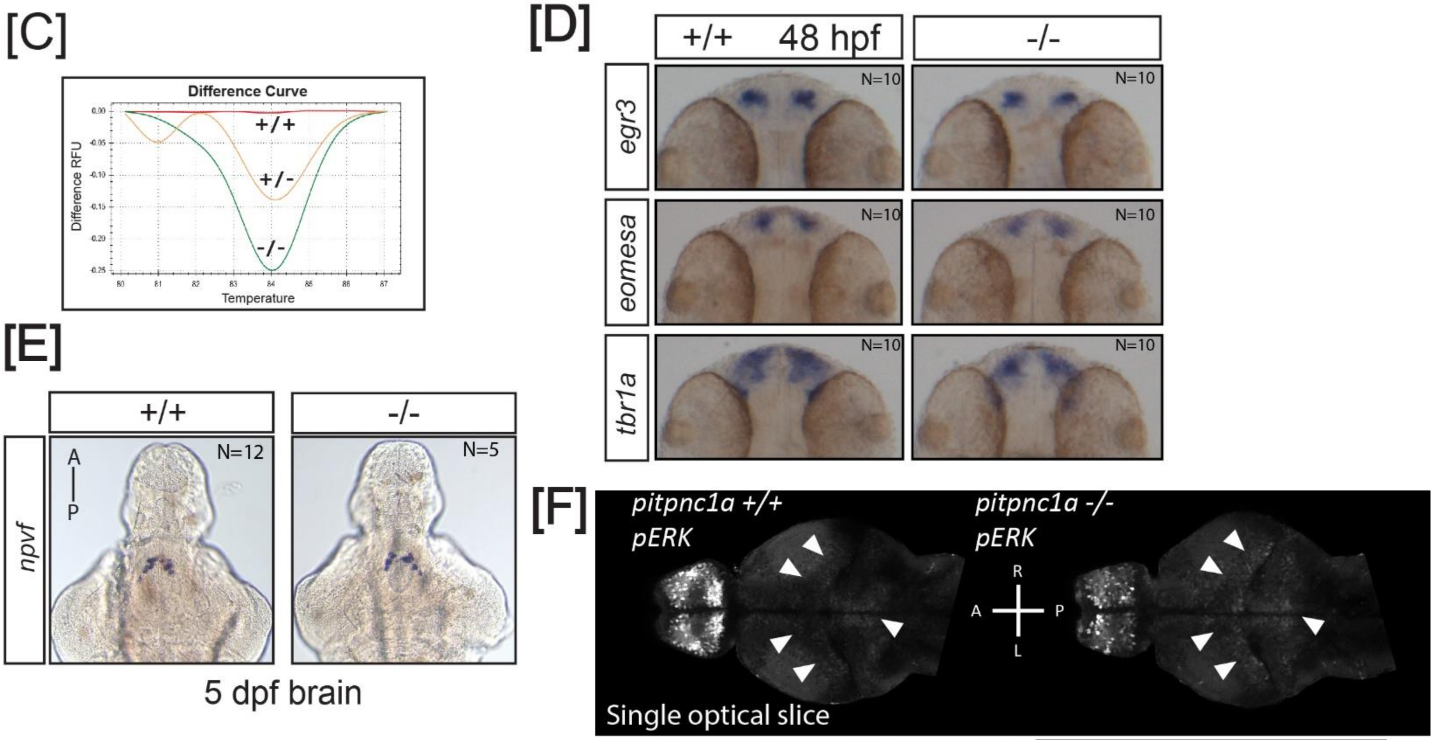

[G]

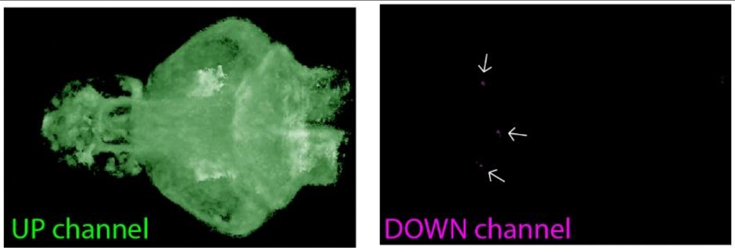

Figure S3-related to Figure 3

Figure S3. A CRISPR/Cas9 generated five base deletion of zebrafish *pitpnc1a* leads to a truncated protein lacking key functional residues, related to Figure 3. A) The *pitpnc1a* Δ5 allele leads to a truncated 336 bp open reading frame. B) Alignment of the full length Pitpnc1a and predicted truncated protein of the *pitpnc1a Δ5* allele. Critical amino acids for binding of the inositol ring of phosphatidylinositol (T59, K61, E86, and N90, mouse numbering) are highlighted, demonstrating the truncated protein lacks two of these critical residues.

C) DNA extracted from whole larvae and subjected to high resolution melt curve analysis (HRMA) is able to distinguish the wild type (+/+, red), heterozygous (+/-, yellow), and homozygous mutant (-/-, green) *pitpnc1a* genotypes. D) In situ hybridization revealed the expression of the dorsal forebrain markers *egr3*, *eomesa*, and *tbr1a* are unaffected in *pitpnc1a*$^{-/-}$ animals. Dorsal views; anterior to the top. E) In situ hybridization for an anterior hypothalamic marker, *npvf*, is unaffected in *pitpnc1a*$^{-/-}$ animals. Ventral views; anterior to the top. F) Representative optical pERK slices (plane 88 of the Z-brain) from wild type and mutant brains stained with pERK/tERK and linearly registered to the Z-Brain reference using the tERK channel. White arrowheads point to examples of areas with upregulated pERK in mutant brains. Images were normalized for intensity using the Stack Normalizer plugin https://imagej.nih.gov/ij/plugins/normalizer.html in Fiji. G) Single channels for the unthresholded maximum projections for the mutant upregulated (green, left) and downregulated (magenta, right) pERK signals.

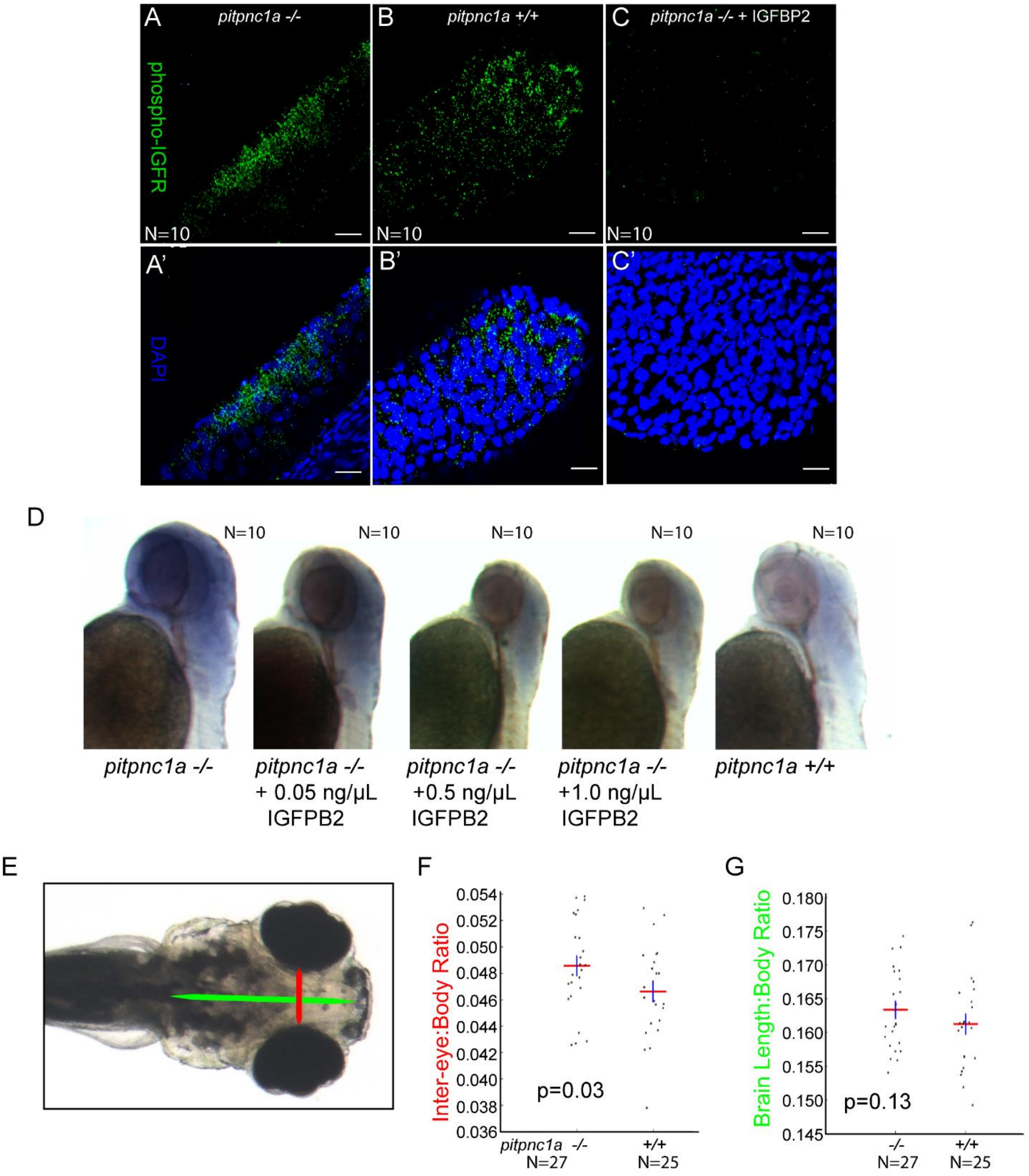

Figure S4-related to Fig 4

Figure S4. IGFBP2 dampens IGF signaling in zebrafish larvae, related to Figure 4. A-C') Confocal images of 48 hpf tailfins labeled with anti-pIGFR antibody (green) reveals numerous puncta in both *pitpnc1a* $^{-/-}$ (B, B') and *pitpnc1a* $^{+/+}$ (C, C') embryos, which are nearly eliminated after soaking in 1 ng/µL IGFBP2 (C, C'). D) IGFBP2 dose-dependently reduces brain *c-fos* levels and reduces growth in *pitpnc1a* $^{-/-}$ embryos. E-G) *pitpnc1a* $^{-/-}$ animals have slightly larger brains than *pitpnc1a* $^{+/+}$ larvae. E) Inter-ocular distance (red line) and brain length (green) were measured relative to total body length (not shown). F) Mutant brains are significantly (4.2%) larger than wild type (p=0.03, one-tailed t-test) as measured by inter-ocular distance G) and trending (1.3%) larger by length (p=0.13, one-tailed t-test). Scale bars, A-C', 20 µm.

Table 1. Behavioral Data

	**pitpnc1a +/+** Mean +/- SEM	**pitpnc1a +/-** Mean +/- SEM	**pitpnc1a -/-** Mean +/- SEM	**ANOVA**
Sleep (min/hr)				
Day 6	8.3 ± 0.9*	6.0 ± 0.5	5.3 ± 0.5*	p=0.03
Night 6	22.3 ± 1.2	19.4 ± 0.7	19.0 ± 1.1	p=0.06
Sleep Bouts (#/hr)				
Day 6	2.7 ± 0.3*	2.0 ± 0.1*	1.8 ± 0.2*	p=0.01
Night 6	7.0 ± 0.2	6.8 ± 0.1	6.7 ± 0.2	p=0.65
Sleep Length (min/bout)				
Day 6	2.7 ± 0.1	2.4 ± 0.9	2.5 ± 0.2	p=0.10
Night 6	3.3 ± 0.2	2.9 ± 0.1	2.8 ± 0.2	p=0.09
Sleep Latency (min)				
Day 6	57.5 ± 18.7	61.8 ± 10.6	74.6 ± 19.5	p=0.76
Night 6	12.6 + 1.2	12.7 ± 0.8	14.6 ± 3.1	p=0.68
Average Activity (s/hr)				
Day 6	244.6 ± 12.1***	267.3 ± 6.8***	303.6 ±12.4***	p=0.001
Night 6	42.2 ± 2.2	44.5 ± 1.1	53.4 ± 3.8	p=0.002
Waking Activity (s/min)				
Day 6	4.4 ± 0.2***	4.8 ± 0.1***	5.3 ± 0.2***	p=0.002
Night 6	1.00 ± 0.04***	1.00 ± 0.02***	1.17 ± 0.07***	p=0.004

Table S1. Sleep-Wake Measurements on 6 dpf *pitpnc1a* mutants and their siblings, related to Figure 2. This table shows mean ± SEM of all behavioral parameters quantified in the primary screen during day and night for all three genotypes. Only the day and night waking activity measurements are strongly different between *pitpnc1a* $^{-/-}$ and their wild type and heterozygous siblings. The *pitpnc1a* mutants also have a modest reduction in daytime sleep compared to wild type, but not heterozygous, animals.

Regions UPREGULATED in *pitpnc1a* mutants

ROI name	Signal in ROI	Top Label	Signal
**Diencephalon - Otpb Cluster 1**	**25981.0186**	**Anti-TH**	**2.5212**
Rhombencephalon - Cerebellar Neuropil 1	25772.6359	Anti-Zrf2	4.3018
Rhombencephalon - Otpb Cluster 1	25277.9683	Vglut2a-GFP	1.8137
Diencephalon - Hypothalamus 6.7FRhcrtR-Gal4 cluster 2	25024.2484	Elavl3-H2BRFP	2.1732
**Diencephalon - Dopaminergic Cluster 4/5 - posterior tuberculum and**	**24980.4805**	**Anti-TH**	**2.1162**
Rhombencephalon - RoL-R1	24697.8915	SpinalBackfills	4.0324
**Diencephalon - Hypothalamus Hcrt Neurons**	**24444.5146**	**Hcrt-RFP**	**6.4504**
**Diencephalon - Anterior group of the posterior tubercular vmat2 neu**	**24422.164**	**Hcrt-RFP**	**3.7191**
**Diencephalon - Dopaminergic Cluster 3 - hypothalamus**	**24407.3153**	**Hcrt-RFP**	**4.1452**
Rhombencephalon - Oculomotor Nucleus nIV	24391.4688	Isl1-GFP	2.6829
Diencephalon - Oxtl Cluster 3	24326.3727	Oxtl-GFP	2.6515
Rhombencephalon - Neuropil Region 6	24130.3709	Isl2bGal4-uasDendra	1.1822
**Diencephalon - Hypothalamus s1181t Cluster**	**23800.4091**	**Qrfp-GFP**	**10.5892**
Rhombencephalon - Gad1b Cluster 3	23632.5067	EtVmat2-GFP	2.3026
**Diencephalon - Hypothalamus Vglut2 Cluster 2**	**23536.9321**	**Vglut2a-GFP**	**2.5232**
**Diencephalon - Dopaminergic Cluster 2 - posterior tuberculum**	**23485.6244**	**Qrfp-GFP**	**3.3125**
Diencephalon - Hypothalamus 6.7FRhcrtR-Gal4 cluster 1	23337.569	6.7FRhcrtR-Gal4-uas	3.5525
Rhombencephalon - Valvula Cerebelli	23328.3832	Ptf1aGal4-uasKaede	1.8429
Mesencephalon - Torus Longitudinalis	23166.4114	Anti-Zrf1(GFAP)	2.1295
Rhombencephalon - RoM1	23108.3556	SpinalBackfills	1.8507
Mesencephalon - Ptf1a Cluster	22919.6422	Ptf1aGal4-uasKaede	1.9644
Diencephalon - Oxtl Cluster 4 - sparse in hypothalamus	22694.3046	EtVmat2-GFP	1.2291
Diencephalon - Migrated Posterior Tubercular Area (M2)	22472.4579	Vglut2a-GFP	1.4983
Diencephalon - Isl1 cluster 3	22368.3456	Elavl3-H2BRFP	2.0971
Rhombencephalon - Oxtl Cluster 1 Sparse	22030.9352	Gad1b-GFP	1.3576
Rhombencephalon - Interpeduncular Nucleus	22016.2018	Anti-Zrf1(GFAP)	1.4155
Rhombencephalon - Locus Coreuleus	21928.3881	EtVmat2-GFP	2.1319
Mesencephalon - NucMLF (nucleus of the medial longitudinal fascicle)	21688.0025	SpinalBackfills	4.7695
Rhombencephalon - Cerebelluar-Vglut2 enriched areas	21641.3931	Vglut2a-GFP	2.1027
Diencephalon - Hypothalamus Gad1b Cluster 2	21586.3209	Qrfp-GFP	2.6368
Diencephalon - Dopaminergic Cluster 6 - hypothalamus	21585.2101	Anti-Zrf1(GFAP)	1.3578
Rhombencephalon - Lobus caudalis cerebelli	21557.0639	Anti-Zn1	1.9184
Mesencephalon - Otpb Cluster	20895.8368	Isl1-GFP	2.1006
Rhombencephalon - Otpb Cluster 2 - locus coeruleus	20874.9172	EtVmat2-GFP	2.9075
Rhombencephalon - Raphe - Superior	20781.7916	EtVmat2-GFP	2.6177
Mesencephalon - Vmat2 cluster of paraventricular organ	20657.8203	EtVmat2-GFP	2.0563
Rhombencephalon - RoM2	20329.8217	SpinalBackfills	3.9807
Rhombencephalon - Gad1b Cluster 17	20272.9659	Gad1b-GFP	1.4565
Mesencephalon - Oculomotor Nucleus nIII	20207.035	Isl1-GFP	1.9912
Rhombencephalon - Gad1b Cluster 2	20183.2407	Gad1b-GFP	1.2934
Diencephalon - Oxtl Cluster 5	20178.0336	Oxtl-GFP	1.456
Rhombencephalon - 6.7FDhcrtR-Gal4 Stripe 4	20023.4785	6.7FRhcrtR-Gal4-uas	2.4838
Rhombencephalon - Cerebellum	19723.509	Ptf1aGal4-uasKaede	3.5764
Mesencephalon - Sparse 6.7FRhcrtR cluster	19695.47	6.7FRhcrtR-Gal4-uas	1.6002
Mesencephalon - Vglut2 cluster 1	19655.5664	Vglut2a-GFP	2.2592
Rhombencephalon - 6.7FDhcrtR-Gal4 Stripe 3	19617.8059	6.7FRhcrtR-Gal4-uas	2.1304
Diencephalon - Otpb Cluster 4	19520.048	Qrfp-GFP	2.8398
Mesencephalon - Oxtl Cluster Sparse	19519.7829	Anti-Zrf1(GFAP)	1.703
Diencephalon - Posterior Tuberculum	19417.4002	Qrfp-GFP	2.3476
Rhombencephalon - Corpus Cerebelli	19340.7978	Ptf1aGal4-uasKaede	2.5346
Diencephalon - Olig2 Band	19308.3995	Olig2-GFP	2.0385
Diencephalon - Medial vglut2 cluster	19140.9075	Vglut2a-GFP	1.6591
Rhombencephalon - Rhombomere 1	19113.368	Ptf1aGal4-uasKaede	2.2558
Diencephalon - Dorsal Thalamus	19062.259	Elavl3-H2BRFP	1.1639
Diencephalon - Retinal Arborization Field 2 (AF2- Approximate Location	19007.1551	Isl2bGal4-uasDendra	11.7149
Mesencephalon - Tegmentum	18843.8642	Elavl3-H2BRFP	1.1758
Rhombencephalon - Gad1b Cluster 7	18827.3396	Ptf1aGal4-uasKaede	2.6501
Diencephalon - Dopaminergic Cluster 1 - ventral thalamic and periventr	18810.4778	Oxtl-GFP	2.8134
Diencephalon - Hypothalamus Gad1b Cluster 1	18709.752	Gad1b-GFP	1.9097
Mesencephalon - Vmat2 cluster2	18664.0675	Elavl3-H2BRFP	1.7239
Diencephalon - Retinal Arborization Field 3 (AF3)	18483.294	Isl2bGal4-uasDendra	4.2447
Diencephalon - Dopaminergic Cluster 7 - Caudal Hypothalamus	18389.0041	Anti-TH	1.3741
Rhombencephalon - RoL3	18322.6043	SpinalBackfills	3.8889
Diencephalon - Oxtl Cluster 2	18307.4762	Isl2bGal4-uasDendra	2.8835
Diencephalon - Ventral Thalamus	17759.7376	Gad1b-GFP	2.226
Diencephalon - Hypothalamus Qrfp neuron cluster	17530.7811	Qrfp-GFP	13.2006

Rhombencephalon - Cerebellum Gad1b Enriched Areas	17521.9014	Ptf1aGal4-uasKaede	4.1139
Rhombencephalon - Eminentia Granularis	17499.9396	Ptf1aGal4-uasKaede	2.7132
Rhombencephalon - Spiral Fiber Neuron Anterior cluster	17238.6357	6.7FRhcrtR-Gal4-uas	5.2927
Rhombencephalon - RoL2	16980.4361	Anti-Zrf2	1.9921
Rhombencephalon - Posterior Cluster of nV Trigeminal Motorneurons	16410.2899	Isl1-GFP	3.052
Rhombencephalon - RoM3	16289.9706	SpinalBackfills	5.3922
Diencephalon - Pretectal dopaminergic cluster	16280.5438	EtVmat2-GFP	2.0998
Rhombencephalon - Spiral Fiber Neuron Posterior cluster	16252.4079	6.7FRhcrtR-Gal4-uas	4.2119
Diencephalon - Anterior pretectum cluster of vmat2 Neurons	16248.0535	EtVmat2-GFP	2.2833
Rhombencephalon - Olig2 enriched areas in cerebellum	16125.4539	Olig2-GFP	2.2364
Diencephalon - Pretectum	15567.4039	EtVmat2-GFP	1.6205
Rhombencephalon - Glyt2 Cluster 8	15432.0625	SpinalBackfills	1.5654
Rhombencephalon - Otpb Cluster 3	15293.4305	S1181tGal4-uasKaed	1.3101
Rhombencephalon - Glyt2 Cluster 7	15277.5816	SpinalBackfills	1.6773
Rhombencephalon - MiR2	15258.2692	SpinalBackfills	3.9808
Diencephalon - Retinal Arborization Field 4 (AF4)	15138.4915	Isl2bGal4-uasDendra	2.6436
Rhombencephalon - RoV3	15064.6138	SpinalBackfills	2.0345
Mesencephalon - Retinal Arborization Field 9 (AF9)	14977.7142	Anti-Zrf2	2.5295
Rhombencephalon - Vmat2 Cluster 1	14959.5042	EtVmat2-GFP	2.576
Rhombencephalon - Vmat2 Cluster 2	14828.3563	EtVmat2-GFP	2.4008
Rhombencephalon - Anterior Cluster of nV Trigeminal Motorneurons	14795.0733	Isl1-GFP	1.905
Rhombencephalon - Otpb Cluster 4	14765.1643	Anti-Zn1	1.2819
Diencephalon - Pretectal Gad1b Cluster	14085.8988	EtVmat2-GFP	2.4021
Rhombencephalon - Gad1b Cluster 15	14032.8187	Elavl3-H2BRFP	1.7698
Diencephalon - Isl1 cluster 2	13900.8569	Elavl3-H2BRFP	2.4804
Rhombencephalon - Isl1 Cluster 2	13731.7645	Isl1-GFP	1.637
Rhombencephalon - Gad1b Cluster 16	13655.763	Isl1-GFP	1.5329
Diencephalon - Isl1 cluster 1	13567.142	Elavl3-H2BRFP	2.1193
Rhombencephalon - Vglut2 cluster 1	13491.4218	Oxtl-GFP	0.96964
Diencephalon - Oxtl Cluster 1 in Preoptic Area	13280.4796	Oxtl-GFP	6.1066
Rhombencephalon - Gad1b Cluster 8	13176.4207	Elavl3-H2BRFP	1.1957
Rhombencephalon - Vglut2 cluster 2	13120.4868	Vglut2a-GFP	3.2905
Rhombencephalon - S1181t Cluster	13025.7548	Vglut2a-GFP	2.7142
Telencephalon - Anterior Commisure	13002.4827	Anti-Zrf2	4.9087
Rhombencephalon - MiR1	12768.2045	SpinalBackfills	4.3695
Mesencephalon - Isl1 cluster of the mesencephalic region	12710.5215	Elavl3-H2BRFP	1.5298
Mesencephalon - Medial Tectal Band	12628.6121	Gad1b-GFP	2.2948
Rhombencephalon - Rhombomere 2	12488.7939	Anti-GlyR	1.5336
Rhombencephalon - Glyt2 Cluster 14	12428.2177	S1181tGal4-uasKaed	2.6781
Rhombencephalon - Glyt2 Cluster 2	12085.8795	EtVmat2-GFP	1.7867
Diencephalon - Migrated Area of the Pretectum (M1)	11991.6766	Anti-Zrf2	1.9103
Rhombencephalon - Glyt2 Cluster 10	11965.2451	Anti-GlyR	1.6519
Rhombencephalon - Glyt2 Cluster 1	11581.9456	Glyt2-GFP	1.7723
Diencephalon - Rostral Hypothalamus	11512.9118	Qrfp-GFP	4.6131
Rhombencephalon - Oxtl Cluster 2 Near MC axon cap	11119.678	Anti-Zn1	1.5114
Diencephalon -	10916.8181	Elavl3-H2BRFP	1.6346
Rhombencephalon - MiM1	10603.0699	SpinalBackfills	5.774
Rhombencephalon - Neuropil Region 5	10387.5568	Anti-GlyR	2.567
Rhombencephalon - Rhombomere 3	10202.2821	Anti-GlyR	1.8715
Rhombencephalon - Vmat2 Cluster 5	10129.1022	EtVmat2-GFP	2.1134
Diencephalon - Intermediate Hypothalamus	10039.5219	Anti-tERK	1.9929
Rhombencephalon - Gad1b Cluster 18	9819.5775	Gad1b-GFP	2.5535
Mesencephalon - Tectum Stratum Periventriculare	9773.364	Elavl3-GCaMP5G	3.1547
Diencephalon - Otpb Cluster 3	9635.6113	Vglut2a-GFP	2.1056
Rhombencephalon - MiV1	9466.2577	SpinalBackfills	3.9809
Rhombencephalon - Gad1b Cluster 1	9436.1355	Gad1b-GFP	1.5393
Rhombencephalon - Isl1 Cluster 1	9304.3085	Isl1-GFP	2.2877
Diencephalon - Hypothalamus Vglut2 Cluster 1	9300.2474	Vglut2a-GFP	1.9122
Rhombencephalon - Spinal Backfill Vestibular Population	9275.8864	SpinalBackfills	4.302
Diencephalon - Otpb Cluster 2	9253.0628	Oxtl-GFP	9.0204
Rhombencephalon -	9103.588	Anti-GlyR	2.4467
Rhombencephalon - Neuropil Region 4	9018.7376	Anti-Znp1(Synaptota	4.2576
Mesencephalon -	8977.596	Isl2bGal4-uasDendra	4.4506
Rhombencephalon - Olig2 Cluster	8815.4639	Olig2-GFP	2.8556
Diencephalon - Retinal Arborization Field 5 (AF5)	8717.035	EtVmat2-GFP	3.6528
Rhombencephalon - Gad1b Cluster 19	8705.9207	Anti-GlyR	3.159
Diencephalon - Diffuse Nucleus of the Intermediate Hypothalamus	8446.8826	Anti-5HT	2.1637
Diencephalon - Olig2 Band 2	8399.2527	Gad1b-GFP	1.1791

Rhombencephalon - Glyt2 Cluster 9	8342.509	Anti-Znp1(Synaptota	1.766
Rhombencephalon - Gad1b Cluster 9	8218.1532	EtVmat2-GFP	1.4168
Rhombencephalon - Vmat2 Cluster 4	8188.5054	EtVmat2-GFP	2.6607
Rhombencephalon - Ptf1a Cluster 1	8118.8909	Ptf1aGal4-uasKaede	1.6627
Telencephalon - Subpallial Vglut2 Cluster	7846.0808	Vglut2a-GFP	4.7758
Diencephalon - Right Habenula Vglut2 Cluster	7748.972	Vglut2a-GFP	5.4079
Diencephalon - Preoptic Area	7726.9329	Oxtl-GFP	2.5649
Rhombencephalon - MiV2	7578.3955	SpinalBackfills	4.0993
Rhombencephalon - Rhombomere 4	7257.3402	Anti-GlyR	2.4306
Diencephalon - Preoptic area posterior dopaminergic cluster	7187.3107	Elavl3-H2BRFP	2.8357
Rhombencephalon - 6.7FDhcrtR-Gal4 Cluster 3	7139.9224	Elavl3-H2BRFP	2.028
Mesencephalon - Torus Semicircularis	6915.9372	Anti-Znp1(Synaptota	2.1369
Rhombencephalon - Mauthner Cell Axon Cap	6793.16	6.7FRhcrtR-Gal4-uas	4.7516
Rhombencephalon - MiD2	6557.1018	SpinalBackfills	5.1494
Rhombencephalon - 6.7FDhcrtR-Gal4 Cluster 1	6507.8588	6.7FRhcrtR-Gal4-uas	2.136
Diencephalon - Caudal Hypothalamus	6187.7406	Anti-GlyR	2.0664
Rhombencephalon - Rhombomere 5	6170.0327	Anti-GlyR	2.2369
Diencephalon - Hypothalamus - Intermediate Hypothalamus Neural Clu	6140.1942	EtVmat2-GFP	1.4936
Diencephalon - Hypothalamus Olig2 cluster 2	5967.6493	Vglut2a-GFP	2.1072
Rhombencephalon - Gad1b Stripe 3	5723.338	Elavl3-H2BRFP	1.3249
Rhombencephalon - Gad1b Cluster 6	5529.5194	S1181tGal4-uasKaed	1.7194
Rhombencephalon - Glyt2 Cluster 13	5508.4537	Anti-Znp1(Synaptota	3.6382
Rhombencephalon - Mauthner	5495.4979	SpinalBackfills	5.1721
Rhombencephalon - VII Facial Motor and octavolateralis efferent neuro	5429.2693	Isl1-GFP	2.048
Rhombencephalon - Medial Vestibular Nucleus	5362.1351	6.7FRhcrtR-Gal4-uas	2.5527
Rhombencephalon - Glyt2 Stripe 3	5361.9914	Elavl3-H2BRFP	1.5001
Rhombencephalon - Gad1b Cluster 13	5243.6938	6.7FRhcrtR-Gal4-uas	2.4967
Rhombencephalon - Tangential Vestibular Nucleus	5233.631	6.7FRhcrtR-Gal4-uas	6.3976
Rhombencephalon - Glyt2 Cluster 3	5189.2915	Glyt2-GFP	2.1791
Rhombencephalon - Rhombomere 6	5170.1871	Vglut2a-GFP	3.2099
Rhombencephalon - Gad1b Cluster 4	5067.4164	Elavl3-H2BRFP	1.4278
Ganglia - Lateral Line Neuromast OC1	5054.0315	Anti-Zrf1(GFAP)	3.9653
Rhombencephalon - Olig2 Stripe	5005.9219	EtVmat2-GFP	1.6519
Rhombencephalon - Gad1b Cluster 5	4909.4778	Elavl3-H2BRFP	1.5384
Diencephalon - Retinal Arborization Field 6 (AF6)	4781.3851	Isl2bGal4-uasDendra	8.1399
Rhombencephalon - 6.7FDhcrtR-Gal4 Cluster 2 Sparse	4778.3954	Elavl3-H2BRFP	1.5556
Telencephalon - Isl1 cluster 1	4755.3119	Gad1b-GFP	2.7935
Telencephalon - Isl1 cluster 2	4641.2773	Gad1b-GFP	2.1503
Rhombencephalon - Gad1b Cluster 10	4571.3911	SpinalBackfills	2.2392
Rhombencephalon - Ventrolateral population of serotonergic neurons	4333.667	Anti-GlyR	1.6709
Rhombencephalon - Raphe - Inferior	4280.5882	Pet1-GFP	2.7938
Rhombencephalon - Vglut2 Stripe 1	4166.3896	Vglut2a-GFP	6.7061
Rhombencephalon - Gad1b Stripe 1	4156.8606	Gad1b-GFP	1.8978
Diencephalon - Habenula	4101.0755	Vglut2a-GFP	5.4536
Rhombencephalon - VII' Facial Motor and octavolateralis efferent neur	4031.4279	Vglut2a-GFP	1.7762
Rhombencephalon - Vglut2 cluster 3	3878.5315	EtVmat2-GFP	1.3907
Telencephalon - Olig2 Cluster	3872.6523	Gad1b-GFP	2.6034
Mesencephalon - Retinal Arborization Field 8 (AF8)	3866.7417	EtVmat2-GFP	2.9323
Diencephalon - Pineal Vmat2 cluster	3663.9802	EtVmat2-GFP	5.0057
Rhombencephalon - 6.7FDhcrtR-Gal4 Stripe 2	3612.9157	6.7FRhcrtR-Gal4-uas	2.408
Telencephalon - Subpallium	3576.9327	Gad1b-GFP	3.2122
Rhombencephalon - Vglut2 Stripe 3	3532.7284	Elavl3-H2BRFP	1.4873
Rhombencephalon - Vglut2 Stripe 4	3381.3756	Vglut2a-GFP	1.6588
Rhombencephalon - Glyt2 Cluster 11	3321.0078	6.7FRhcrtR-Gal4-uas	2.656
Mesencephalon - Tecum Neuropil	3318.6878	Isl2bGal4-uasDendra	7.6604
Rhombencephalon - Glyt2 Cluster 6	3145.5263	Elavl3-H2BRFP	1.5583
Rhombencephalon - Otpb Cluster 5	3097.0262	S1181tGal4-uasKaed	2.066
Telencephalon - Subpallial Otpb Cluster 2	2901.4851	Gad1b-GFP	2.3515
Rhombencephalon - Isl1 Cluster 3	2826.002	Vglut2a-GFP	1.4495
Rhombencephalon - Glyt2 Cluster 5	2767.0874	Elavl3-H2BRFP	1.6957
Ganglia - Statoacoustic Ganglion	2576.675	Elavl3-H2BRFP	2.8031
Rhombencephalon - Vmat2 Cluster 3	2416.4021	6.7FRhcrtR-Gal4-uas	2.7124
Rhombencephalon - Gad1b Cluster 12	2316.9806	SpinalBackfills	2.8084
Rhombencephalon - Glyt2 Cluster 12	2315.2386	6.7FRhcrtR-Gal4-uas	2.9278
Telencephalon -	2238.306	Anti-Zrf2	3.5627
Telencephalon - Telencephalic Migrated Area 4 (M4)	2209.1071	Anti-Zrf2	3.6968
Rhombencephalon - Vglut2 Stripe 2	2196.7309	Elavl3-H2BRFP	1.6476
Diencephalon - Left Habenula Vglut2 Cluster	2190.1366	Vglut2a-GFP	5.1551

Telencephalon - Subpallial Gad1b cluster	2175.3643	Gad1b-GFP	4.2321
Rhombencephalon - MiD3	2162.7295	SpinalBackfills	4.6403
Diencephalon - Hypothalamus Vglut2 Cluster 5	2066.5736	Vglut2a-GFP	1.5754
Diencephalon - Pineal	2020.9022	Anti-TH	4.7874
Diencephalon - Preoptic Otpb Cluster	1997.6182	Anti-Zrf2	3.1383
Rhombencephalon - Qrfp neuron cluster sparse	1927.5861	Vglut2a-GFP	5.7229
Rhombencephalon - Vmat2 Stripe1	1647.006	Elavl3-H2BRFP	3.0626
Telencephalon - Vglut2 rind	1640.981	Vglut2a-GFP	3.6075
Rhombencephalon - 6.7FDhcrtR-Gal4 Cluster 4	1567.8025	6.7FRhcrtR-Gal4-uas	2.88
Diencephalon - Eminentia Thalami	1543.9245	Vglut2a-GFP	5.2082
Rhombencephalon - 6.7FDhcrtR-Gal4 Stripe 1	1510.3111	Gad1b-GFP	3.7183
Diencephalon - Hypothalamus Vglut2 Cluster 3	1411.1358	Anti-tERK	2.5902
Rhombencephalon - MiT	1326.4305	Anti-Znp1(Synaptota	1.5978
Telencephalon - Olfactory Bulb	1318.1344	Anti-Zrf1(GFAP)	2.2991
Rhombencephalon - Ptf1a Stripe	1304.9609	Gad1b-GFP	3.0223
Telencephalon - S1181t Cluster	1288.9228	Gad1b-GFP	1.9741
Rhombencephalon - Glyt2 Cluster 4	1207.5299	Glyt2-GFP	1.7452
Rhombencephalon - 6.7FDhcrtR-Gal4 Cluster 5	1196.9757	6.7FRhcrtR-Gal4-uas	2.5026
Ganglia - Lateral Line Neuromast SO3	1093.2377	Anti-Zrf1(GFAP)	5.0817
Diencephalon - Hypothalamus - Caudal Hypothalamus Neural Cluster	1037.0168	Anti-GlyR	2.1094
Telencephalon - Pallium	1026.6531	Vglut2a-GFP	3.2722
Diencephalon - Hypothalamus Gad1b Cluster 3 Sparse	1023.2356	Anti-GlyR	3.0996
Diencephalon - Retinal Arborization Field 1 (AF1- Approximate Location	950.8348	Isl2bGal4-uasDendra	3.3186
Telencephalon - Postoptic Commissure	924.856	Anti-Zrf2	4.2737
Rhombencephalon - Neuropil Region 2	910.6231	Anti-Znp1(Synaptota	4.0571
Rhombencephalon - Neuropil Region 3	910.5548	Anti-GlyR	2.98
Diencephalon - Hypothalamus Vglut2 Cluster 6	677.2467	Anti-tERK	2.6876
Diencephalon - Postoptic Commissure	594.5268	Qrfp-GFP	4.2828
Rhombencephalon - Rhombomere 7	585.4897	Gad1b-GFP	4.5624
Rhombencephalon - Gad1b Cluster 11	570.2383	Elavl3-H2BRFP	1.7336
Telencephalon - Optic Commissure	547.3087	Isl2bGal4-uasDendra	23.5869
Rhombencephalon - Glyt2 Stripe 2	534.1997	Elavl3-H2BRFP	1.6053
Diencephalon - Anterior preoptic dopaminergic cluster	491.3548	Gad1b-GFP	3.6267
Rhombencephalon - Gad1b Cluster 14	304.9341	6.7FRhcrtR-Gal4-uas	2.4987
Rhombencephalon - Glyt2 Stripe 1	294.9262	Elavl3-H2BRFP	1.7704
Diencephalon - Preoptic area Vglut2 cluster	271.119	Elavl3-H2BRFP	2.2216
Rhombencephalon - Vmat2 Stripe2	239.9459	6.7FRhcrtR-Gal4-uas	1.7529
Rhombencephalon - X Vagus motorneuron cluster	154.2477	Elavl3-H2BRFP	1.9454
Rhombencephalon - Otpb Cluster 6	131.0848	Elavl3-H2BRFP	1.9067
Ganglia - Lateral Line Neuromast SO2	109.4393	Otpb.A-Gal4-UAS-GC	3.0218
Ganglia - Facial Sensory Ganglion	105.8146	Elavl3-H2BRFP	3.0389
Telencephalon - Vmat2 cluster	103.7865	Vglut2a-GFP	2.378
Ganglia - Anterior Lateral Line Ganglion	78.0785	Elavl3-H2BRFP	4.4
Ganglia - Olfactory Epithelium	61.0479	Elavl3-H2BRFP	4.6833
Ganglia - Lateral Line Neuromast N	59.6494	Anti-GlyR	NaN
Ganglia - Trigeminal Ganglion	58.9868	Isl2bGal4-uasDendra	3.2657
Mesencephalon - Retinal Arborization Field 7 (AF7)	42.9445	Isl2bGal4-uasDendra	5.8098
Spinal Cord - 6.7FDhcrtR-Gal4 Stripe	33.9127	Elavl3-H2BRFP	2.9276
Rhombencephalon - Gad1b Stripe 2	33.9108	Elavl3-H2BRFP	1.7682
Diencephalon - Hypothamic VentroLateral VMAT cluster	19.0024	Elavl3-H2BRFP	5.0399
Rhombencephalon - Vglut2 cluster 4	12.8417	EtVmat2-GFP	6.3303
Telencephalon - Olfactory bulb dopaminergic neuron areas	8.0333	Gad1b-GFP	2.8758
Rhombencephalon - Caudal Ventral Cluster Labelled by Spinal Backfills	5.6987	SpinalBackfills	2.5794

Regions DOWNREGULATED in *pitpnc1a* mutants

ROI name	Signal in ROI	Top Label	Signal
Telencephalon - Pallium	5.5763	Anti-tERK	3.7072
Telencephalon - Vglut2 rind	4.8038	Vglut2a-GFP	4.3117
Telencephalon -	2.7383	Anti-Zrf2	5.7175
Rhombencephalon - Rhombomere 7	0.11117	Pet1-GFP	5.2942
Rhombencephalon -	0.04976	Pet1-GFP	6.3857

Table S2. MAP-Mapped brain regions and transgenic lines that overlap with up- and down-regulated pERK signals in *pitpnc1a*$^{-/-}$ larvae, related to Figure 3. Differential pERK signal in *pitpnc1a*$^{-/-}$ versus wild type larvae were morphed into the Z-brain atlas to identify annotated brain regions and the most correlated, overlapping transgenic lines. As Z-brain uses overlapping identifiers to name brain sub-regions, some areas and transgenes are represented multiple times.

Supplementary Table 3 - Enriched Drug Classes that Cluster with *pitpnc1a* mutant phenotypes

Rank	Name	Correlation
**PDE Inhibitor**		
1	Skf 94836	0.9353
6	Fosfosal	0.8754
7	Propentofylline	0.8733
24	Ibudilast	0.8004
33	Papaverine	0.7479
36	Ro20-1724	0.7377
**NSAID**		
16	Fenoprofen	0.8359
20	Aspirin	0.8243
26	Bufexamac	0.7931
**Glucocorticoids**		
4	Flunisolide	0.8953
12	Clobetasol	0.8489
30	Desoxycorticosterone	0.7541
32	Betamethasone	0.7509
34	Flumethasone	0.7443
42	Medrysone	0.7184
48	Hydrocortisone	0.7103
**Other Anti-inflammatory**		
5	Aminophenazone	0.8782
8	Capsazepine	0.8600
17	Nicotine Ditartrate	0.8321
25	(-)-Nicotine	0.7980
29	Theaflavin	0.7567
37	Diphenylpyraline	0.7283
38	Valproate	0.7264
**NMDA Antagonists**		
3	L-701,324	0.9083
10	Sinapic Acid, Methyl Ester	0.8571
11	Dizocilpine	0.8492
15	L-701,324	0.8457
19	L-701,324	0.8258
45	(-)-Mk801	0.7117
47	7-Chlorokynurenic Acid	0.7108

Table S3. Anti-inflammatory compounds with behavioral fingerprints that co-cluster with *pitpnc1a*$^{-/-}$ larvae, related to Figure 3. The compounds are organized by functional class, in order of rank (1=most closely correlated with *pitpnc1a* $^{-/-}$). Weighted Pearson correlation coefficients are also listed.

Extended Methods

Antibodies

A custom rabbit polyclonal antibody was raised to a specific peptide from zebrafish Pitpnc1a called PAb:520; the peptide sequence used for immunisation was LPSLFSWGSSSPQPE (Eurogentec). A rabbit polyclonal antibody raised against a specific peptide from human PITPNC1 called RB59 was a kind gift from S.F.Tavazoie. RB59 was raised to the C-terminus of human PITPNC1-sp1 with a sequence of DPEKKATLNLPGMHSSDK. Antibodies against PITPα (PAb:674) and PITPβ (MAb 4A7) were raised in house and have been described elsewhere (Carvou et al., 2010). Antibodies against the FLAG-tag and pan 14-3-3 were obtained commercially from Origene (TA50011) and Santa Cruz Biotechnology (sc-629), respectively. Antibodies against phospho-IGF1 receptor beta (Y1135) (#3918) were obtained from Cell Signaling Technologies, and antibody against GAPDH was obtained from Thermo (MA5-15738).

Identification and alignment of zebrafish PITPNC1 orthologs

A BLASTP search was carried out within the *Danio rerio* protein database (Refseq, NCBI) using the amino acid sequence of human PITPNC1-sp1 as a query (NP_036549.2). The obtained sequences of the zebrafish orthologues were truncated, therefore further EST analysis was then performed to obtain the full-length protein coding sequences by aligning up and downstream EST sequences.

Isolation of zebrafish cDNA

Total RNA was prepared from 24 hpf embryos using the RNeasy Plus Mini Kit (Qiagen). Around 30 embryos were homogenised in 350 µl of Buffer RLT plus and frozen at -80ºC until ready for purification. The lysate was then thawed and centrifuged at 13,000 rpm for 3 min to remove any debris and pigmentation. The resulting supernatant was subjected to spin column purification following the manufacturer's protocol. Total RNA was then used for a reverse transcriptase reaction using Superscript II (Life Technologies) according to the manufacturer's instructions.

PCR

RT-PCR against regions of *pitpnc1a* (LOC563621) and *pitpnc1b* (ENSDARG00000022807) was carried out using our zebrafish cDNA library as a template. PCR reactions were completed using Platinum Taq Polymerase (Life Technologies). The resulting PCR products were analysed by agarose gel electrophoresis and then purified using a QIAquick PCR purification kit (Qiagen). The PCR products were cloned using the pGEM-T easy vector system (Promega) and their sequence was verified by Sanger sequencing (MWG Eurofins).

pitpnc1a Forward Primer 5' - CGGATTCCAGGAGTCATTTC

pitpnc1a Reverse Primer 5' - TGTCTGTGACCCAAGAGCAG

pitpnc1b Forward Primer 5' - TCCTACCGAGGTTCCATGTC

pitpnc1b Reverse Primer 5' - ATTGAATGGTTTCGCTCCAG

Cloning of the complete coding sequence with an additional FLAG-tag

RT-PCR was carried out on our zebrafish cDNA library to amplify the complete coding sequences of *pitpnc1a* and *pitpnc1b* using PfuTurbo Hot Start (Agilent Technologies) according to the manufacturer's instructions. An N-terminal FLAG epitope tag (DYKDDDDK) was engineered during the PCR reaction. The resulting PCR products were analysed by gel electrophoresis and then restriction digested with BamHI and HindIII (Promega). The digested product was ligated into the mammalian expression vector PCDNA3.1. The sequence of the resulting vector was verified by Sanger sequencing (MWG Eurofins).

FLAG-Pitpnc1a_Dr Forward Primer 5' - GACGGATCCCCACCATGGATTACAAGGATGACGACGATAAGGCAGTGTTGATGAAGGAATACCGGATATG

FLAG- Pitpnc1a_Dr Reverse Primer 5' - CTAAAGCTTTTATTACTCGGGCTGCGGGCTGCTG

FLAG- Pitpnc1b_Dr Forward Primer 5' - GACGGATCCCCACCATGGATTACAAGGATGACGACGATAAGGCAGTGTTGGTCAAAGAGTACCGGATATGCATGCCGCTC

FLAG- Pitpnc1b_Dr Reverse Primer 5' - CTAAAGCTTTTATTATTCTGGGGTTGATTTTAATCTCACAGGACCCTTG

Sub cloning with 6XHis-tag

The coding sequence of *pitpnc1a* was sub cloned by PCR from the FLAG-Pitpnc1a_Dr vector using PfuTurbo Hot Start (Agilent Technologies) according to the manufacturer's instructions. The N-terminal FLAG epitope tag (DYKDDDDK) was replaced with a 6xHis epitope tag during the PCR reaction. The resulting PCR products were analysed by gel electrophoresis and then restriction digested with BamHI and HindIII (Promega). The digested product was ligated into the mammalian expression vector PCDNA3.1. The sequence of the resulting vector was verified by Sanger sequencing (MWG Eurofins).

6xHis-Pitpnc1a_Dr Forward Primer 5' - GACGGATCCCCACCATGCATCATCATCATCATCATGCAGCAGTGTTGATGAAGGAATACCGGATATG

6xHis-Pitpnc1a_Dr Reverse Primer 5' - CTAAAGCTTTTATTACTCGGGCTGCGGGCTGCTG

Protein purification

293F cells were grown to a density of 1.0-1.4 x 10^6 cells/mL in a volume of 1 L. The cells were transfected with plasmid DNA using polyethylenenimine (PEI). To prepare the transfection mix 40 mL of OptiPro was mixed with L-glutamine to a final concentration of 4 mM. The resulting mixture was subsequently filter sterilised through a 0.22 µm filter. 1.25 mg of plasmid DNA was then added along with 1.875 mg of PEI and this mixture was allowed to complex at room temperature for 10 min. The PEI:DNA complex was added to the cells and the protein was harvested after 3-4 days of culture. To purify the protein cells were

harvested by centrifugation at 3000 rpm for 15 min at 4°C. The cell pellet was then re-suspended in 20 mL of ice-cold lysis buffer (25 mM Tris/HCl pH 7.4, 250 mM NaCl, 20 mM Imidazole, 10 mM Benzamidine) that was supplemented with protease inhibitor cocktail I and II (Sigma Aldrich). The cells were then lysed by sonication (on ice) and the resulting lysate was cleared by centrifugation at 18000 rpm for 2 hr at 4°C. The supernatant was added to a HIS-Select Nickel Affinity gel as described previously (Fensome et al., 1996). The recombinant proteins were desalted into Pipes buffer (20 mM Pipes, 137 mM NaCl and 3 mM KCl, pH 6.8) and analysed by SDS/PAGE for purity. The protein concentration was adjusted accordingly and the proteins were stored at −80ºC. From a 1 L prep, only 100 µg of protein was obtained.

Lipid transfer assays

Phosphatidylinositol (PI) transfer activity was assayed by measuring the transfer of [^{3}H]-PI from radiolabelled rat liver microsomes to unlabelled synthetic liposomes [Phosphatidylcholine (PC)/PI molar ration of 98:2] by recombinant Pitpnc1a_Dr (250 µL, 10 µg/µL), as described previously (Thomas et al., 1993). Phosphatidic acid (PA) transfer activity was assayed by measuring the transfer of [^{3}H]-PA from liposomes to unlabelled rat liver mitochondrial preparations by recombinant Pitpnc1a_Dr (250 µL, 10 µg/µL) (Yadav et al., 2015). Transfer activity was calculated as a percentage of the total radioactivity present in the assay after subtraction of the number of counts transferred in the absence of a recombinant source. Transfer activity was monitored in duplicate samples.

Electroporation of Cos-7 cells

Cos-7 cells were transfected with either pcDNA3.1 or pcDNA3.1-FlagPITPNC1 by electroporation. The cells were trypsinised and mixed with 10 µg of the respective construct in a sterile electroporation cuvette. For electroporation two pulses of 0.220 kV and 950 µF were delivered and the cells were transferred directly to ice for 5 min. Cells were then transferred to a tissue culture flask and returned to the incubator (37°C, 5% CO_2). Cells were ready for experimental use 48 hr post transfection.

Immunoprecipitations

Cells were harvested by trypsinisation and were washed with ice cold PBS. The cells were re-suspended in PIPES buffer (20 mM Pipes, 137 mM NaCl and 3 mM KCl, pH 6.8) supplemented with protease and phosphatase I and II inhibitors (Sigma–Aldrich, P8340, P2850 and P5726 respectively). The cells were sonicated and the membrane fraction was pelleted by centrifugation (50,000 rpm for 1 hr at 4°C), the cytosol fraction (supernatant) was retained for further analysis. The BCA (bicinchoninic acid) assay was used to determine the protein concentration of the cytosol fraction. For immunoprecipitation, 1200 µg of cytosolic protein was incubated with 50 µL of equilibrated anti-FLAG M2 affinity gel (Sigma–Aldrich, A2220) on a rotating wheel for 1 hr at 4°C. The tubes were centrifuged at 10,000 rpm for 2 min (4°C) and the supernatant was discarded. The beads were washed four times with PIPES buffer; the final wash was carried out in a fresh Eppendorf tube. After the final centrifugation, the supernatant was aspirated and replaced with 30 µL of NuPAGE LDS sample buffer under reducing conditions (Life Technologies).

Cos-7 protein preparation

The Cos-7 cell monolayer was washed with ice cold PBS and the cells were then harvested with RIPA buffer (50 mM Tris/HCl pH 8, 150 mM NaCl, 1% Triton-X 100, 0.5% Sodium Deoxycholate, 0.1% SDS) supplemented with protease inhibitors (Sigma Aldrich) on ice. The lysates were briefly sonicated and then cleared by centrifugation at 15,000 rpm, 4°C. The BCA assay was used to determine the protein concentration of the samples.

Zebrafish tissue protein preparation

Zebrafish tissues were homogenised with RIPA buffer (50 mM Tris/HCl pH 8, 150 mM NaCl, 1% Triton-X 100, 0.5% Sodium Deoxycholate, 0.1% SDS) supplemented with protease inhibitors (Sigma Aldrich) on ice. The homogenates were briefly sonicated and then cleared by centrifugation at 15,000 rpm, 4°C. The BCA assay was used to determine the protein concentration of the samples.

Cytosol protein preparation from rat tissues

Rat tissues were homogenized in PIPES buffer (20 mM PIPES, 137 mM NaCl and 3 mM KCl, pH 6.8) in the presence of a protease inhibitor cocktail (Sigma). To prepare membranes and cytosol, the lysates were centrifuged for 10 minutes (15,000 rpm, 4°C) to pellet the nuclei and unbroken cells. The lysate was then centrifuged for 1 hour at 50,000 rpm, 4°C (rotor type TLA55, Beckman Optima benchtop ULTRA) to pellet the membranes, and the supernatant consisting of the cytosolic fraction was retained for further analysis.

HEK293 standard preparation

1 x 10^6 HEK293 cells were seeded into a 10 cm dish and allowed to adhere overnight (37ºC, 5% CO_2). The cells were then transfected using FugeneHD (Promega) with FLAG-Pitpnc1a according to the manufacturer's instructions. The cells were ready for experimental use 24 hr after transfection.

Western blot analysis

Protein samples were prepared in NuPAGE LDS Sample Buffer under reducing conditions (Life Technologies) and were boiled prior to loading on the gel (70°C, 5 min). SDS-PAGE was carried out using a Novex NuPAGE gel electrophoresis system (Life Technologies). Within this system the samples were analysed on NuPAGE 4-12% Bis-Tris gels (1.5 mm thick, 10-well) according to the manufacturer's instructions. After SDS-PAGE the proteins were transferred to an Immobilin-P PVDF membrane (Millipore) for blotting. The following primary antibody dilutions were used; PAb:520 = 1:100; GAPDH = 1:3000; FLAG = 1:2000; 14-3-3 = 1,1000; PAb:647 = 1:1000; MAb:4A7 = 1:1000; RB59 = 1:50.

Whole-mount in situ hybridisation (colorimetric)

Digoxigenin (DIG)-labelled sense and antisense riboprobes corresponding to regions of *pitpnc1a*, *pitpnc1b*, *npvf*, and *galanin* mRNA were synthesized using T7 and SP6 RNA polymerases (Roche) using linearized

PGEMT vectors as a template. DIG-labelled anti-sense probes for *egr3*, *tbr1a*, *eomesa* and *fosab* were a kind gift from the Steve Wilson Group (UCL).

Prior to in situ hybridization, dechorionated embryos were fixed at the appropriate stage using 4% Paraformaldehyde (PFA) in PBS overnight at 4°C. The following day the embryos were rinsed into PBST and bleached (3% H_2O_2, 1% KOH) for 10-15 minutes at room temperature. Adult brains were dissected and fixed using 4% PFA in PBS overnight at 4°C. The samples were then dehydrated through a PBST:methanol series (25%, 50%, 75%, 100%) each step taking 5 minutes at room temperature. Embryos were stored in 100% methanol at -20°C at least for overnight and until ready for use.

Embryos that were prepared for brain dissection were fixed with 4% PFA and 4% Sucrose in PBS overnight at 4°C, followed by the brain dissection with sharp forceps and methanol dehydration and storage (as above).

Embryos or dissected brains were then rehydrated back through a methanol:PBST series (25%, 50%, 75%, 100%) each step taking 5 minutes at room temperature. The samples were then subjected to a proteinase K treatment followed by a post fixation for 20 minutes at room temperature in 4% PFA in PBS.

Samples were treated in pre-hybridisation buffer (50% formamide, 5X SSC, 0.1mg/mL tRNA, 0.01 mg/mL heparin, 9.2 mM citric acid, 0.1% Tween 20) at 67°C for at least 1 hour. Probes were then added and the samples were incubated at 67°C overnight. The samples were then subjected to washes to remove excess unbound probes. The hybridisations were developed using nitroblue tetrazolium (NBT)/5-bromo-4-chloro-3-indolyl phosphate (BCIP) substrate (Roche). Whole-mount specimens were mounted in 80% glycerol and photographed with a Nikon SMZ1500 microscope mounted with a Leica MC190HD camera.

Immunohistochemistry

Wild type and *pitpnc1a*$^{-/-}$ larvae were fixed overnight at 4ºC in 4% PFA, washed 2 times for 5 min in PBST, then dehydrated through a MeOH series (25%, 50%, 75%, 100%) and stored at -80ºC. Larvae were rehydrated in PBST and the brains were exposed by dissection of the skin and head with forceps. Mutant and wild type larvae were then placed in the same tubes for all subsequent steps, with the mutants distinguished from wild type by cutting the tail. Larvae were washed 3X in PBS then blocked for 2 hr in 10% goat serum, 1%DMSO, PBT, then incubated overnight at 4ºC with anti-pIGFR beta (Y1135 DA7A8) diluted 1:500 in blocking solution. The next day, larvae were washed 6X in PBT for 15 min, then incubated overnight at 4ºC with goat anti-rabbit Alexa 488 (A11034, Life Technologies) at 1:200. Larvae were then washed 6X in PBT before mounting in 60% glycerol for imaging with a confocal microscope.

Supplemental References

Carvou, N., Holic, R., Li, M., Futter, C., Skippen, A., and Cockcroft, S. (2010). Phosphatidylinositol- and phosphatidylcholine-transfer activity of PITPbeta is essential for COPI-mediated retrograde transport from the Golgi to the endoplasmic reticulum. J. Cell Sci. *123*, 1262–1273.

Fensome, A., Cunningham, E., Prosser, S., Tan, S.K., Swigart, P., Thomas, G., Hsuan, J., and Cockcroft, S. (1996). ARF and PITP restore GTP gamma S-stimulated protein secretion from cytosol-depleted HL60 cells by promoting PIP2 synthesis. Curr. Biol. CB *6*, 730–738.

Thomas, G.M.H., Cunningham, E., Fensome, A., Ball, A., Totty, N.F., Troung, O., Hsuan, J.J.C., and S. (1993). An essential role for phosphatidylinositol transfer protein in phospholipase C-mediated inositol lipid signalling. Cell *74*, 919–928.
