# Supplementary material for: Pitpnc1a Regulates Zebrafish Sleep and Wake Behavior through Modulation of Insulin-like Growth Factor Signaling
Source: Cell Rep. 2018 Aug 7;24(6):1389–96. doi: 10.1016/j.celrep.2018.07.012 (PMC6092267; doi:10.1016/j.celrep.2018.07.012)
Supplement: Document S2. Article plus Supplemental Information [file mmc2.pdf]

Cell Reports

Report

Pitpnc1a Regulates Zebrafish Sleep and Wake Behavior through Modulation of Insulin-like Growth Factor Signaling

Graphical Abstract

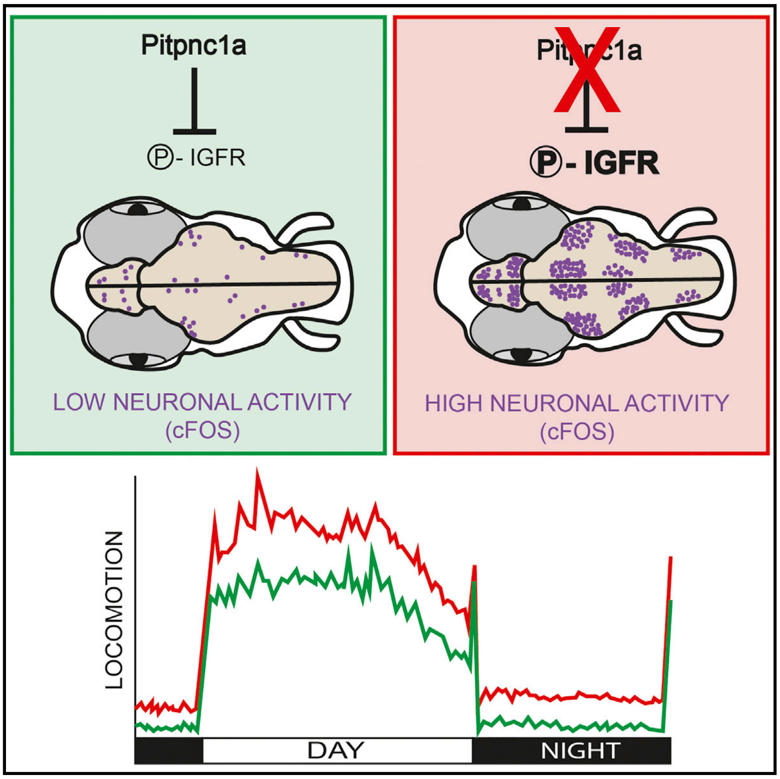

Highlights

- Zebrafish have two orthologs, one brain enriched, of the lipid transporter PITPNC1
- CRISPR/Cas9-generated *pitpnc1a* mutants are hyperactive across the day-night cycle
- Insulin-like growth factor signaling is dysregulated in *pitpnc1a* mutants
- Blocking IGF signaling rescues *pitpnc1a* mutant behavioral and neuronal hyperactivity

Authors

Tim G. Ashlin, Nicholas J. Blunsom, Marcus Ghosh, Shamshad Cockcroft, Jason Rihel

Correspondence

s.cockcroft@ucl.ac.uk (S.C.),
j.rihel@ucl.ac.uk (J.R.)

In Brief

Ashlin et al. find that CRISPR/Cas9 zebrafish mutants that lack the brain-enriched lipid transporter Pitpnc1a have dysregulated insulin-like growth factor (IGF) signaling and behavioral hyperactivity. This work suggests that Pitpnc1a normally regulates the set point of neuronal excitability by dampening IGF.

Ashlin et al., 2018, Cell Reports *24*, 1389–1396
August 7, 2018 © 2018 The Author(s).
https://doi.org/10.1016/j.celrep.2018.07.012

Cell Reports
Report

OPEN ACCESS
CellPress

Pitpnc1a Regulates Zebrafish Sleep and Wake Behavior through Modulation of Insulin-like Growth Factor Signaling

Tim G. Ashlin,[1,3] Nicholas J. Blunsom,[1] Marcus Ghosh,[2] Shamshad Cockcroft,[1,*] and Jason Rihel[2,4,*]
[1]Department of Neuroscience, Physiology and Pharmacology, University College London, London WC1E 6BT, UK
[2]Department of Cell and Developmental Biology, University College London, London WC1E 6BT, UK
[3]Present address: The Francis Crick Institute, 1 Midland Rd, Kings Cross, London NW1 1AT, UK
[4]Lead Contact
*Correspondence: s.cockcroft@ucl.ac.uk (S.C.), j.rihel@ucl.ac.uk (J.R.)
https://doi.org/10.1016/j.celrep.2018.07.012

SUMMARY

The lipid transporters of the phosphatidylinositol transfer protein (PITP) family dictate phosphoinositide compartmentalization, and specific phosphoinositides play crucial roles in signaling cascades, membrane traffic, ion channel regulation, and actin dynamics. Although PITPs are enriched in the brain, their physiological functions in neuronal signaling pathways *in vivo* remain ill defined. We describe a CRISPR/Cas9-generated zebrafish mutant in a brain-specific, conserved class II PITP member, *pitpnc1a*. Zebrafish *pitpnc1a* mutants are healthy but display widespread aberrant neuronal activity and increased wakefulness across the day-night cycle. The loss of Pitpnc1a increases insulin-like growth factor (IGF) signaling in the brain, and inhibition of IGF pathways is sufficient to rescue both neuronal and behavioral hyperactivity in *pitpnc1a* mutants. We propose that Pitpnc1a-expressing neurons alter behavior via modification of neuro-modulatory IGF that acts on downstream wake-promoting circuits.

INTRODUCTION

Lipids are essential signaling molecules, with tight controls governing their levels and subcellular compartmentalization. One class of proteins with major roles in lipid regulation are the phosphatidylinositol transfer proteins (PITPs). PITPs bind and transfer phosphatidylinositol (PI), thereby regulating the levels of phosphoinositides, including PI(4,5)bisphosphate. PITPs have been especially implicated in neuronal lipid regulation to alter functions ranging from phototransduction to neurite outgrowth (Cockcroft, 2012). However, the diversity of PITPs means much is yet to be discovered about critical functions of PITPs in the brain.

The function of PITPNC1 in neurons has not been examined as extensively as it has for other PITP family members, such as RdgBα and PITPα. PITPNC1 has two isoforms generated via alternative splicing: the long variant PITPNC1-sp1 and the short variant PITPNC1-sp2 (Takano et al., 2003). These isoforms likely have distinct biochemical and functional properties, because only PITPNC1-sp1 can bind 14-3-3 proteins (Garner et al., 2011), and they are expressed in distinct regions of the embryonic mammalian brain (Takano et al., 2003). PITPNC1 has also been implicated in breast cancer cell metastasis: PITPNC1 expression is a key target of the metastasis-suppressing microRNA, miR-126 (Png et al., 2011); up to 46% of breast cancer tumors show amplified PITPNC1 expression; and an interaction between PITPNC1 and RAB1B at the Golgi was shown to drive malignant secretion of molecules that promote metastasis (Halberg et al., 2016). How PITPNC1's role in pathogenic breast cancer relates to its endogenous function remains unknown.

To identify PITPNC1's physiological function *in vivo*, we used CRISPR/Cas9 to create a zebrafish null mutant of a brain-specific ortholog of the human long isoform *pitpnc1a*. We find that *pitpnc1a* mutants have widespread, elevated neuronal activity and increased wakefulness across the 24 hr day-night cycle. Because insulin-like growth factor (IGF) signaling is upregulated in *pitpnc1a* mutants and inhibition of this pathway restores mutant behavior, we propose that Pitpnc1a modulates IGF signaling cascades to control the set point of neuronal excitability.

RESULTS

A Zebrafish Pitpnc1 Ortholog Binds PI and PA

To find zebrafish *pitpnc1*, we used the long human splice variant PITPNC1-sp1 (NP_036549.2) in zebrafish genome database queries (release GRCz10). Basic Local Alignment Search Tool – Protein (BLASTP) searches and expressed sequence tag (EST) alignments identified two zebrafish orthologs on chromosome 3 (renamed *pitpnc1a*), and chromosome 16 (renamed *pitpnc1b*) (Figure 1A; Figure S1A). The zebrafish *pitpnc1a* gene is predicted to encode a 331 amino acid protein that shows 81% identity (90% similarity) with the human long splice variant PITPNC1-sp1 (Figure S1B), while *pitpnc1b* encodes a shorter 305 amino acid protein similar to human PITPNC1-sp2 and only 57% identity (71% similarity) to human PITPNC1-sp1 (Figure S1B). Both zebrafish proteins contain the PITP family, inositol ring-coordinating amino acids (T59, K61, E86, and N90) (Tilley

This is an open access article under the CC BY license (http://creativecommons.org/licenses/by/4.0/).

OPEN
ACCESS
CellPress

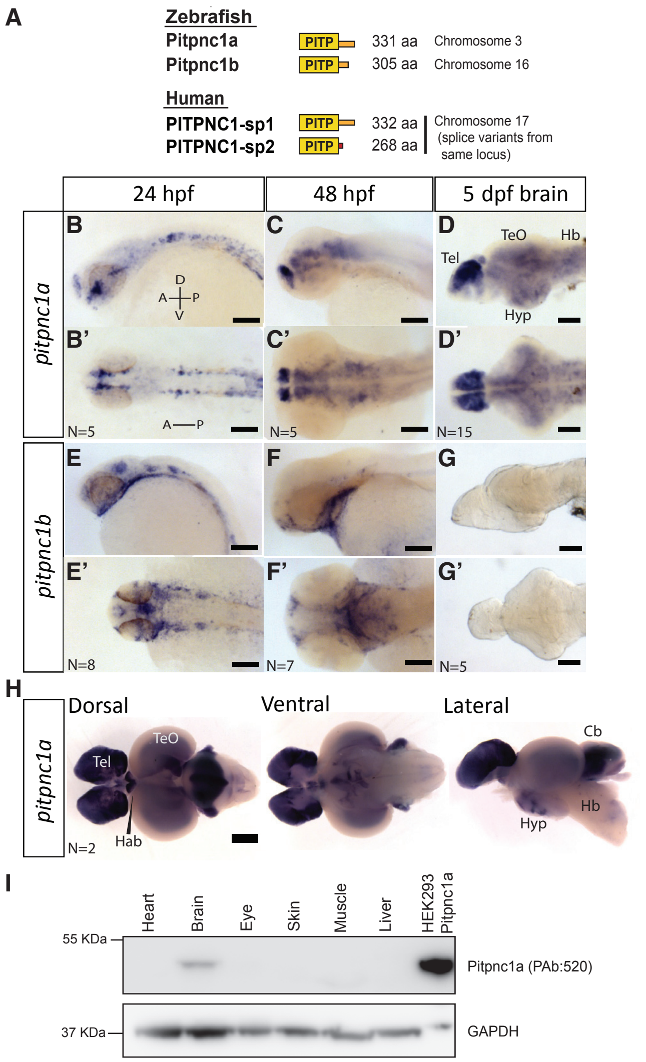

Figure 1. One Zebrafish PITPNC1 Ortholog Is Exclusively Expressed in the Brain

(A) Zebrafish PITPNC1 orthologs and human splice variants.

(B–D′) *pitpnc1a* mRNA is detected in the nervous system at 24 hpf (B and B′), 48 hpf (C and C′), and is widespread in the brain by 5 dpf (D and D′).

(E–G′) *pitpnc1b* mRNA is expressed in non-neuronal tissue, including the pharyngeal arches and olfactory pits (E–F′). *pitpnc1b* mRNA is undetectable in the larval brain (G and G′).

(H) *pitpnc1a* mRNA is expressed in many areas of the adult zebrafish brain.

(I) Pitpnc1a protein is detected only in the adult zebrafish brain. Transfected HEK293 cells are a positive control.

Tel, telencephalon; TeO, optic tectum; Hyp, hypothalamus; Hb, hindbrain; Hab, habenula; Cb, cerebellum. Scale bars, 100 μm (B–G′) and 500 μm (H).

et al., 2004), but only Pitpnc1a retains potential 14-3-3 binding serine residues (Figure 1A; Figure S1B).

Using purified His- and FLAG-tagged Pitpnc1a protein, we tested whether zebrafish Pitpnc1a has biochemical properties similar to those of the human protein. Pitpnc1a-His was able to transfer both PI and PA (phosphatidic acid) *in vitro* at levels comparable to the human PITPNC1-sp1 isoform (Figures S2A and S2B). Immunoprecipitation of transfected, FLAG-tagged versions from Cos-7 cell lysates and probing for 14-3-3 proteins on a western blot revealed that both the human PITPNC1-sp1 and the zebrafish Pitpnc1a bound 14-3-3 proteins, whereas zebrafish Pitpnc1b did not (Figures S2C and S2D). Altogether, these data indicate that the zebrafish Pitpnc1a is capable of transferring both PI and PA and binding 14-3-3, similar to the human PITPNC1-sp1 protein.

pitpnc1a Is Expressed in the Larval and Adult Zebrafish Brain

PITPNC1-sp1 has been detected in the adult mouse heart and brain, with specific enrichment in the dentate gyrus, thalamus, and Purkinje layer of the cerebellum (Garner et al., 2011; Takano et al., 2003). In zebrafish, we detected *pitpnc1a* transcripts by *in situ* hybridization (ISH) in several regions of the developing CNS by 24 hr post fertilization (hpf), including the dorsal forebrain, midbrain, and bilateral clusters of cells in the spinal cord (Figures 1B and 1B′). At 48 hpf, *pitpnc1a* expresses highly in the developing forebrain, midbrain, hypothalamus, and hindbrain (Figures 1C and 1C′). By larval stages (5 days post fertilization [dpf]), *pitpnc1a* expression is exclusively and extensively detected throughout the brain, with particularly strong expression in the dorsal telencephalon (Figures 1D and 1D′). *pitpnc1a* mRNA is also detectable in these areas in the adult zebrafish brain, with strong expression in the forebrain, habenula, and cerebellum, as well as expression in the optic tectum and several hypothalamic and hindbrain nuclei (Figure 1H). We did not detect *pitpnc1a* transcripts in the heart or other non-neuronal tissues. In contrast, *pitpnc1b* transcripts were excluded from the CNS at all stages, with the exception of some expression at 24 hpf around the developing brain ventricles that subsequently is undetectable (Figures 1E–1G′). Expression of *pitpnc1b* is localized to the pharyngeal arches and olfactory vesicles by 48 hpf (Figures 1F and 1F′), as well as in the pronephric duct (data not shown). At 5 dpf, there are no detectable *pitpnc1b* transcripts in the brain (Figures 1G and 1G′). The distinct tissue distribution of *pitpnc1a* and *pitpnc1b* supports the hypothesis that these genes have non-overlapping functions *in vivo*.

To detect Pitpnc1a protein, we developed a polyclonal antibody against the unique C terminus of zebrafish Pitpnc1a that shows no cross-reactivity with Pitpnc1b (Figure S2E). Western blot analysis detected Pitpnc1a protein only in the adult zebrafish brain, with no detectable protein in heart, liver, eye, skin, or muscle (Figure 1I). For comparison, a cross-reacting antibody against human PITPNC1-sp1 detects PITPNC1-sp1 protein in the adult rat heart and brain (Figure S2F). The restricted expression of Pitpnc1a allows functions of vertebrate PITPNC1 in the brain to be experimentally isolated *in vivo*.

Zebrafish *pitpnc1a* Null Mutants Display Behavioral Hyperactivity

To knock out Pitpnc1a function, we used CRISPR/Cas9 to introduce a 5-base pair deletion into exon 2 of *pitpnc1a* (Figure 2A) that is easily detected by high-resolution melt curve analysis (Figure S3C). This deletion introduces a frameshift to make a predicted truncated protein that lacks two key inositol binding

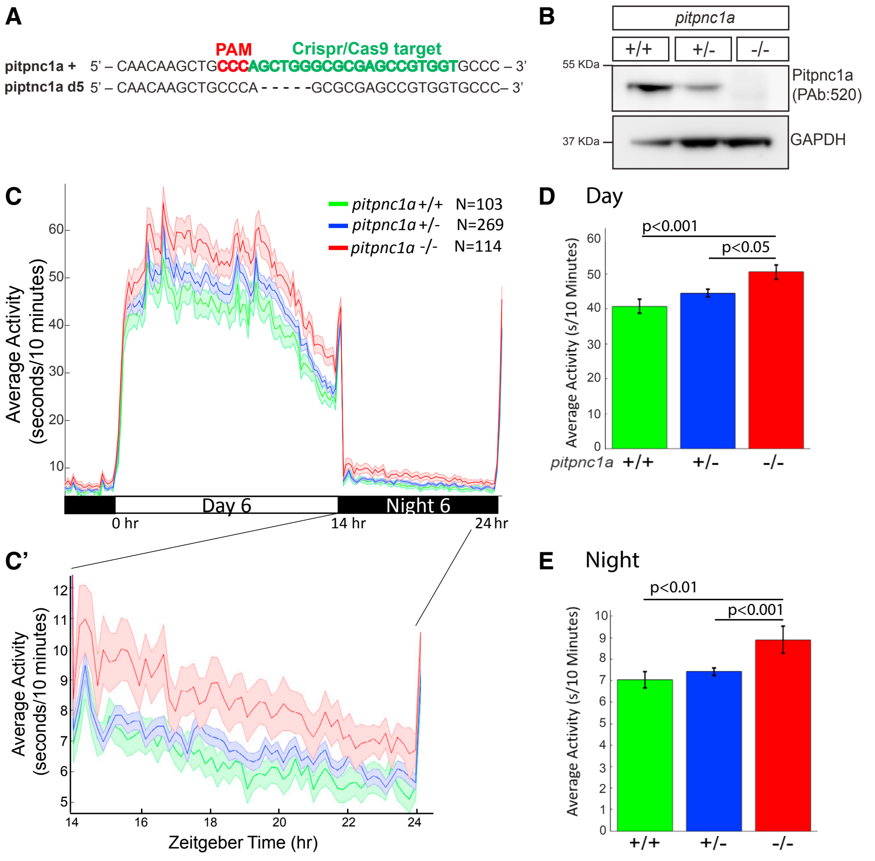

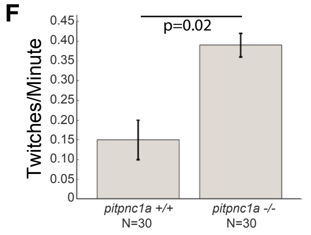

G Touch Assay

	% Response		
Genotype	36 hpf	52 hpf	incross 52 hpf
wild type	29.4 (20/68)	34.2 (65/190)	36.6 (15/41)
pitpnc1a -/-	53.4 (61/113)	51.3 (119/232)	66.7 (16/24)
Difference (± cI)	24 (8.4-38.0)	17.1 (7.3-26.5)	30.1 (2.6-53.0)
Chi-squared	9.851	12.4	5.4
p-value	0.0002	0.0004	0.02

Figure 2. *pitpnc1a*$^{-/-}$ Larvae Are Behaviorally Hyperactive

(A) CRISPR/Cas9 generated a 5-base pair deletion in exon 2 of *pitpnc1a*.

(B) Pitpnc1a protein is detected in *pitpnc1a*$^{+/+}$ brain lysates, weaker in *pitpnc1a*$^{+/-}$, and undetectable in *pitpnc1a*$^{-/-}$.

(C) An activity plot across a 14:10 hr day-night cycle (zoomed in C′) reveals hyperactivity in 6 dpf *pitpnc1a*$^{-/-}$. The shaded ribbons represent ± SEM.

(D and E) Bar plots (±SEM) of the mean day (D) and night (E) activity. *pitpnc1a*$^{-/-}$ animals are significantly more active during both day and night (one-way ANOVA, Tukey's post hoc test).

(F) At 36 hpf, *pitpnc1a*$^{-/-}$ embryos twitch significantly more than *pitpnc1a*$^{+/+}$ (mean ± SEM, one-way ANOVA).

(G) At both 36 and 52 hpf, *pitpnc1a*$^{-/-}$ embryos are significantly more touch sensitive (pooled data from 5 and 4 independent experiments, respectively, and repeated in a blinded pitpnc1a$^{+/-}$ incross; chi-square test).

residues (N90 and E86) (Figures S3A and S3B). Homozygous mutant *pitpnc1a*$^{\Delta5/\Delta5}$ adult brains lack detectable Pitpnc1a protein by western blot (Figure 2B), indicating the Δ5 *pitpnc1a* allele is likely functionally null, and mutants will hereafter be called *pitpnc1a*$^{-/-}$.

Larval and adult homozygous *pitpnc1a*$^{-/-}$ mutants appear visually indistinguishable from wild-type siblings and are viable and fertile, and the Δ5 allele is inherited in Mendelian ratios (e.g., Figure 2C). Expression analysis of dorsal forebrain (*egr3*, *tbr1a*, and *eomesa*) and hypothalamic (*npvf*) markers found no changes in *pitpnc1a*$^{-/-}$ larvae, suggesting that Pitpnc1a is not required for the specification or differentiation of these areas that strongly express *pitpnc1a* (Figures S3D and S3E).

Because Pitpnc1a is exclusively detected in the zebrafish brain, including areas that have been implicated in larval sleep and arousal (Barlow and Rihel, 2017), we asked whether behavior might be affected in *pitpnc1a*$^{-/-}$ larvae by video-tracking them for several days (Rihel et al., 2010). Mutants showed a consistent increase in waking activity across a 14:10 hr light:dark cycle relative to *pitpnc1a*$^{+/+}$ and *pitpnc1a*$^{+/-}$ (Figures 2C–2E). Larval sleep during the day was also mildly reduced in both *pitpnc1a*$^{+/-}$ and *pitpnc1a*$^{-/-}$ larvae (Table S1). Because expression of *pitpnc1a* was detectable by 24 hpf, we also tested whether early embryonic behaviors were affected. Mutant embryos spontaneously coiled (i.e., twitched) significantly more often than wild-type controls at 36 hpf (Figure 2F) and were significantly more touch sensitive at both 36 and 52 hpf (Figure 2G). These results suggest that Pitpnc1a modulates the sensitivity of neurons involved in both stimulus-evoked and spontaneous behaviors.

pitpnc1a$^{-/-}$ Mutants Have Increased Neuronal Activity in Arousal-Related Circuits

To map changes in neuronal activity that might underlie the mutant behavior, we raised zebrafish in constant darkness through 6 dpf, conditions that result in low expression of the immediate early gene, *c-fos*, in wild-type larvae (Figures 3B and 3B′). In contrast to dark-reared wild-type larvae, which have barely detectable *c-fos* expression, *pitpnc1a*$^{-/-}$ mutants exhibited strong and extensive *c-fos* expression (Figures 3A and 3A′). The *c-fos* expression was particularly strong in a broad swath of the dorsal forebrain, two bilateral clusters in the ventral forebrain, the preoptic area, the midbrain, the posterior hypothalamus, and a bilateral cluster of neurons in the cerebellum (Figures 3A and 3A′). To determine whether these populations are similarly engaged during acute arousal, we exposed larvae

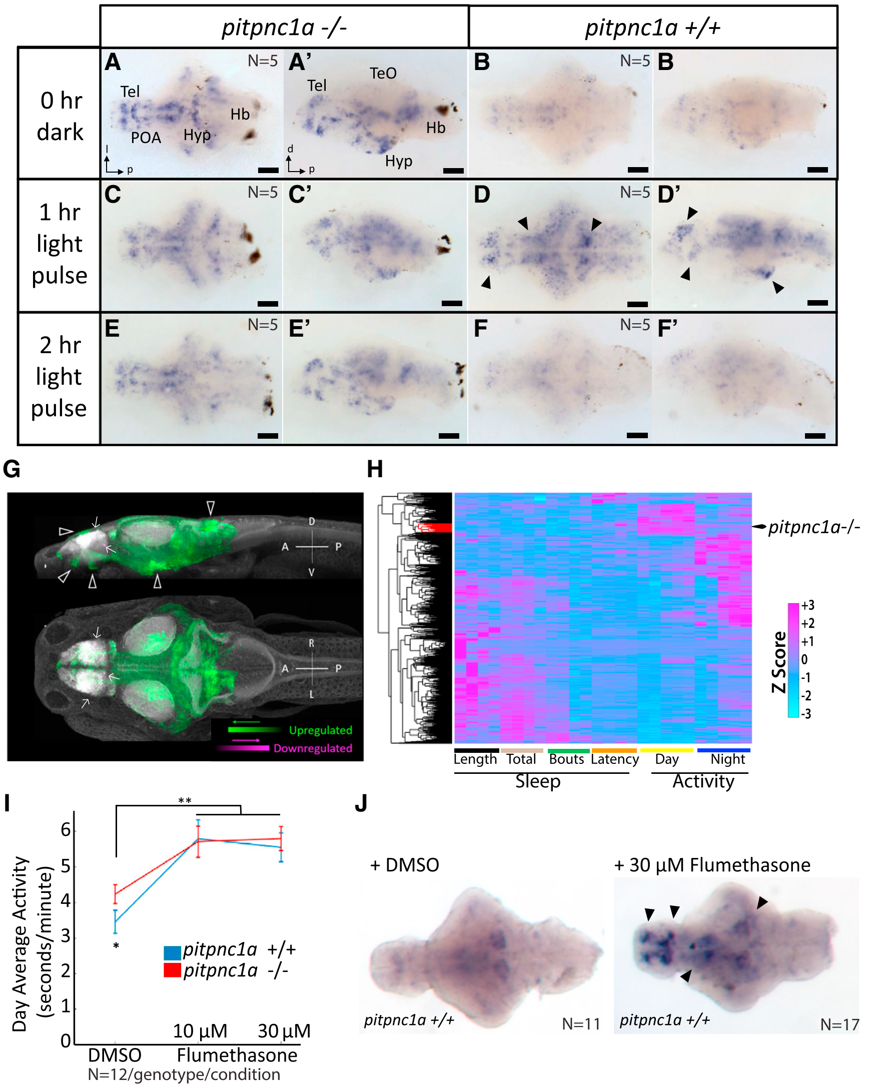

Figure 3. *pitpnc1a*$^{-/-}$ Brains Have Increased Neuronal Activity

(A–B′) ISH reveals many areas of *c-fos* expression in 6 dpf *pitpnc1a*$^{-/-}$ larval brains (A and A′) compared with wild-type (B and B′).

(C–D′) Following a 1 hr light pulse, additional neurons express *c-fos* in *pitpnc1a*$^{-/-}$ (C and C′) and wild-type (D and D′). Many areas expressing *c-fos* in wild-type after a light pulse overlap with strong *c-fos* signals in *pitpnc1a*$^{-/-}$ brains at 0 hr (black arrowheads, D and D′).

(E–F′) After 2 hr of light, *c-fos* levels remain elevated in *pitpnc1a*$^{-/-}$ brains (E and E′) but have returned to baseline in wild-type (F and F′).

(G) pERK/tERK comparison of dark-reared, 6 dpf *pitpnc1a*$^{-/-}$ (n = 15) and wild-type (n = 12) brains reveals mutant brain areas with upregulated (green) and downregulated (magenta) expression of pERK relative to controls. White arrowheads indicate areas of agreement between pERK and *c-fos* expression, and white arrows show the downregulated signal. Data are shown as a thresholded maximum projection overlaid on a maximum projection of the Z-Brain tERK reference (gray).

(H) Clustering the behavioral fingerprint of *pitpnc1a*$^{-/-}$ against 550 drug-induced behavioral profiles places the phenotype in a strong day-active and modestly night-active cluster (red branches).

(I) Flumethasone increases the behavioral activity of both wild-type and *pitpnc1a*$^{-/-}$ to the same level. DMSO-treated *pitpnc1a*$^{-/-}$ animals are hyperactive (*p < 0.05, one-way ANOVA, Tukey's post hoc test). Both 10 and 30 μM flumethasone increase larval activity (**p < 0.01, two-way ANOVA, gene × dose, Tukey's post hoc test).

(J) Flumethasone induces *c-fos* expression in areas similar to *pitpnc1a*$^{-/-}$ and light pulses (black arrows).

Hb, hindbrain; POA, preoptic area. (A–F) Dorsal view. (A′–F′) Lateral view. (J) Ventral view. Scale bars, 100 μm.

to a light pulse. Following 1 hr light exposure, wild-type brains had increased *c-fos* expression in many areas that overlapped with those regions upregulated in *pipnc1a*$^{-/-}$ larvae in the dark, including those in the dorsal and ventral forebrain, midbrain, and posterior hypothalamus (black arrowheads, Figures 3D and 3D′). Additional circuits involved in the detection and sensorimotor transformation of light also upregulated *c-fos* expression, including the optic tectum and hindbrain (Figures 3C and 3D). This indicates that the baseline *c-fos* expression in *pitpnc1a* mutants can be further elevated, at least in some neurons. After 2 hr of light exposure, *c-fos* levels have returned to near pre-pulse levels, with *pitpnc1a*$^{-/-}$ maintaining higher expression than wild-type (Figures 3E and 3E′). Thus, many neuronal areas activated by light in wild-type are already active in *pitpnc1a*$^{-/-}$ larvae in the dark, which is consistent with the increased mutant behavioral arousal during both day and night.

To complement the *c-fos* evidence, we used a measure of neuronal activity based on changes in the ratio of phosphorylated ERK (pERK) to total ERK (tERK), which has faster kinetics relative to *c-fos* transcription (Randlett et al., 2015). Relative to wild-type, *pitpnc1a*$^{-/-}$ larvae have widespread neuronal activation, as detected by increased pERK/tERK ratio, with extensive overlap with areas observed by *c-fos*, including the dorsal and ventral forebrain, midbrain, and hypothalamus (Figure 3G; Figures S3F and S3G). However, pERK upregulation also extends to areas not observed by *c-fos* expression, including the optic tectum and hindbrain, which may reflect the faster kinetics or higher sensitivity of pERK relative to *c-fos*. Morphing this data onto the Z-Brain reference library identified brain regions and neuronal subtypes that are most strongly enriched for upregulated pERK activity in *pitpcn1a*$^{-/-}$ brains (Table S2). This includes two dopaminergic populations of the diencephalon and the hypocretin neurons of the anterior hypothalamus (Figure 3G), which is consistent with an upregulation of known arousal circuits in the diencephalon. Other upregulated areas overlap with areas implicated in arousal, including the locus coeruleus

and dorsal raphe (Table S2). In contrast, few mutant brain areas show a lower relative pERK signal (Figure 3G; Figures S3F and S3G; Table S2).

Wake-Inducing Glucocorticoids Converge on Neurons Activated in *pitpnc1a* Mutants

If *pitpnc1a* mutants are hyperactive due to dysregulation of wake-promoting neurons, we speculated that wake-inducing drugs would activate the same neuronal populations. To identify likely drugs, we normalized the *pitpnc1a*$^{-/-}$ behavioral parameters to wild-type to generate a behavioral fingerprint (Rihel et al., 2010), which was then hierarchically clustered against a dataset of 550 drug-induced behaviors (Figure 3H). The *pitpnc1a*$^{-/-}$ cluster was enriched with anti-inflammatory compounds (46% of hits with a correlation coefficient > 0.7), including glucocorticoids (7 of the top 50), non-steroidal anti-inflammatory drugs (NSAIDs; 3/50), and phosphodiesterase inhibitors (6/50) (Table S3). This cluster is also enriched for NMDA antagonists (7/50). Thus, the *pitpnc1a*$^{-/-}$ behavioral arousal converges onto drugs with shared biological properties that increase zebrafish wakefulness.

To test whether these drugs and the *pitpnc1a* mutation are altering behavior via common or parallel pathways, we examined *pitpnc1a* mutant and wild-type responses to the glucocorticoid flumethasone. As expected, flumethasone increased the waking activity of wild-type animals (Figure 3I). However, flumethasone only increased the waking activity of *pitpnc1a*$^{-/-}$ larvae to the drug-treated wild-type level, indicating that *pitpnc1a*$^{-/-}$ and flumethasone are non-additive and likely converge onto common neuronal circuits. Consistent with this interpretation, flumethasone upregulated *c-fos* expression in similar neuronal populations that are upregulated in *pitpnc1a*$^{-/-}$ animals (Figure 3J).

IGFR Signaling Is Upregulated in *pitpnc1a*$^{-/-}$ Brains

Because PITPNC1 enhances the secretion of pro-metastatic factors and insulin growth factor binding protein 2 (IGFBP2) (Halberg et al., 2016; Png et al., 2011), we tested whether IGF signaling was altered in *pitpnc1a*$^{-/-}$ brains. We used antibodies against IGF-1 receptor beta phosphorylated at Y1135 (hereafter called pIGFR) to detect activation of the IGF pathway. We detected several pIGFR puncta in the dorsal telencephalon of 6 dpf wild-type brain that were even more extensive in *pitpnc1a*$^{-/-}$ brains (Figures 4A and 4B). In the ventral hypothalamus, significantly fewer pIGFR puncta were detected in wild-type brains compared to mutants (Figures 4C–4E). In contrast, in the trunk and tail, which lack Pitpnc1a, pIGFR levels were unaffected (Figures S4A and S4B). To confirm that these puncta represent bona fide pIGFR, we soaked wild-type and mutant larvae in IGFBP2, which abolished nearly all pIGFR puncta (Figure S4C). If IGF signaling is upregulated in the *pitpnc1a*$^{-/-}$ brains, IGF should stimulate increased *pitpnc1a*$^{-/-}$ brain growth (Duan et al., 1999). We found that *pitpnc1a* mutants had a significant increase in the ratio of intra-ocular brain width to body length and a trend toward an increased brain length (Figures S4E–S4G). Thus, *piptnc1a* mutants have increased insulin-like growth factor receptor (IGFR) signaling and an increase in brain growth.

Inhibition of IGF Signaling Rescues *pitpnc1a*$^{-/-}$ Neuronal Activity and Behavior

To test whether increased IGF signaling leads to altered mutant behavior, we first soaked larvae overnight from 36 to 48 hpf in 1 ng/μL recombinant human IGFBP2 and assessed neuronal activity by *c-fos* expression. Similar to 5 dpf brains, untreated *pitpnc1a* mutants had elevated *c-fos* levels, but overnight exposure to IGFBP2 reduced mutant *c-fos* back to wild-type levels (Figure 4F; Figure S4D).

We next tested whether inhibition of phosphatidylinositol 3-kinase (PI3K) and Akt, components downstream of IGFR signaling, could affect the aberrant neural and behavioral hyperactivity in *pitpnc1a* mutants. Overnight (>16 hr) soaking of *pitpnc1a*$^{-/-}$ larvae in either the PI3K inhibitor LY294002 (2 μM) or the allosteric Akt inhibitor MK-2206 (100 nM) rescued *c-fos* staining from strong upregulation in mutants (Figures 4G and 4H) to wild-type levels (Figures 4I and 4J). Soaking *pitpnc1a*$^{-/-}$ larvae in 1 μM MK-2206 also resulted in a reversion of the mutant night-time hyperactivity to wild-type levels (Figure 4K), suggesting that upregulated IGFR signaling in *pitpnc1a* mutants leads to aberrant neuronal and behavioral hyperactivity.

DISCUSSION

We found that the brain-specific, class II lipid transfer protein Pitpnc1a is essential for maintaining proper neuronal activity and wakefulness via modulation of IGF signaling. Our work not only has implications for understanding the impact of phospholipids on behavior but also may have clinical relevance, because PITPNC1 is upregulated in many metastatic tumors (Halberg et al., 2016) and polymorphisms in PITPNC1 have been linked to type 2 diabetes mellitus (Greenawalt et al., 2012; Liu et al., 2017).

Pitpnc1a Modulates Baseline Behavioral Activity

Zebrafish have two highly conserved and differentially expressed orthologs of the human PITPNC1 gene, *pitpnc1a* and *pitpnc1b*, which correspond to the long and short human isoforms, respectively. Evolution often redistributes a protein's expression and function across duplicated genes, or ohnologs (Pasquier et al., 2016), and this feature allowed us to isolate the role of Pitpnc1 proteins in the brain by targeting zebrafish *pitpnc1a*.

CRISPR/Cas9-generated *pitpnc1a* mutants are hyperactive across the day:night cycle and exhibit increased neuronal activity as measured by both *c-fos* and pERK. Many of the neuronal populations that are most strongly upregulated in *pitpnc1a*$^{-/-}$ larvae overlap with neurons implicated in setting levels of zebrafish wakefulness, including arousing hypothalamic and hindbrain populations. In addition, the non-additivity and neuronal convergence between wake-promoting glucocorticoids and *pitpnc1a* mutants indicates that neurons of the ascending arousal system may act in concert to affect *pitpnc1a* mutant hyperactivity. The human PITPNC1 gene resides within a copy number variant associated with a syndromic intellectual disability caused by loss of the neighboring gene PSMD12 (Küry et al., 2017). Individuals that have lost a larger region encompassing PITPNC1 often exhibit hyperactivity.

OPEN ACCESS
CellPress

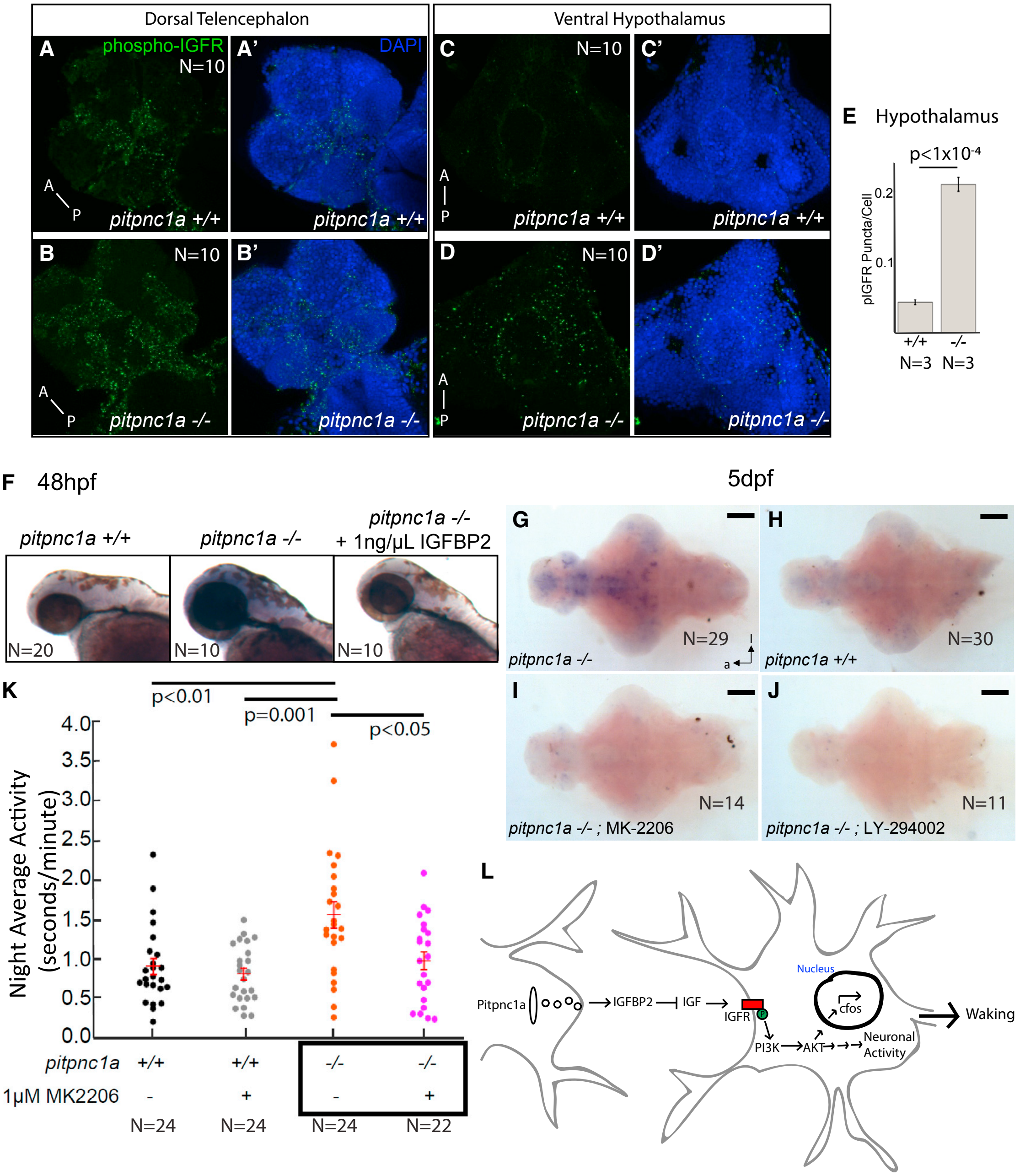

Figure 4. IGFR Signaling Is Enhanced in *pitpnc1a*$^{-/-}$ Brains, and Inhibiting IGFR Signaling Rescues Neuronal and Behavioral Hyperactivity

(A–E) 6 dpf *pitpnc1a*$^{-/-}$ larvae have increased pIGFR puncta in the brain. (A–B′) Confocal projections of the dorsal telencephalon in wild-type (A and A′) and *pitpnc1a*$^{-/-}$ (B and B′) reveal pIGFR puncta (green). Nuclei are DAPI stained (blue) in (A′) and (B′). Confocal projections of the *pitpnc1a*$^{-/-}$ ventral hypothalamus (D and D′) have more pIGFR puncta (C and C′), as quantified in (E) (mean ± SEM, one-way ANOVA).

(F) Upregulated *c-fos* in *pitpnc1a*$^{-/-}$ embryos is reduced to wild-type by soaking in 1 ng/µL IGFBP2 from 36 to 48 hpf.

(G–J) ISH for *c-fos* in 6 dpf brains in constant dark after overnight exposure to PI3K (LY-294002, 2 µM) or Akt (MK-2206, 100 nM) inhibitors. (G and H) *c-fos* is upregulated in *pitpnc1a*$^{-/-}$ brains (G) relative to WT controls (H). (I and J) Akt (I) and PI3K (J) inhibitors reduce mutant *c-fos* expression to wild-type levels. Brains in (G)–(J) are overdeveloped to expose even weak *c-fos* expression.

(K) Exposure to 1 µM Akt inhibitor, MK-2206, reduces average night-time activity of 6 dpf *pitpnc1a*$^{-/-}$ larvae to wild-type levels. Each dot represents a single larva, and the crossbars plot the mean ± SEM (two-way ANOVA, genotype × drug interaction, Tukey's post hoc test).

(L) Model of Pitpnc1a modulation of neuronal activity via IGFBP2 and inhibition of IGF-PI3K-Akt. Loss of Pitpnc1a enhances IGF signaling, leading to increased waking behavior.

(G–J) Ventral view; scale bars, 100 µm. (A–B′) Scale bars, 100 µm. (C–E′) Scale bars, 20 µm.

Pitpnc1a, IGF, and Neuronal Activity

The *pitpnc1a* mutants had increased activation of IGF signaling, and blocking this cascade upstream with IGFBP2 or downstream with PI3K and Akt inhibitors was sufficient to restore neuronal and behavioral activity. Taking this data together with observations in the cancer literature, we propose a model (Figure 4L) in which Pitpnc1a stimulates the secretion of inhibitory IGFBP2 that acts as an IGF counterbalance onto circuits involved in wakefulness. When Pitpnc1a is missing, IGF signaling is too high, leading to PI3K-Akt activation, upregulation of neuronal activity, and ultimately increased wakefulness.

Altered PI3K-Akt signaling has been implicated in a variety of neurodevelopmental and psychiatric diseases, including autism (Chen et al., 2014) and schizophrenia (Zheng et al., 2012), but the mechanisms by which this may affect neuronal circuits remains obscure. In light of the data that Pitpnc1a modulates IGF-PI3K-Akt signaling in the vertebrate brain, the role of lipid transporters in the progression, mitigation, or exacerbation of these complex mental disorders should be further investigated.

EXPERIMENTAL PROCEDURES

Zebrafish Strains

AB/TL zebrafish were maintained at 28.5°C by the University College London (UCL) Fish Facility. *pitpnc1a*$^{-/-}$ animals were generated using CRISPR/Cas9 and outcrossed back to AB/TL for at least 3 generations. Work was in accordance with the Animal Experimental Procedure Act (1986) under license 70/7612.

CRISPR/Cas9 Targeting *pitpnc1a* and Genotyping

CRISPR/Cas9 targeting of *pitpnc1a* exon 2 was carried out as described (Hwang et al., 2013), using the guide RNA sequence (5′-ACCACGGCTC GCGCCCAGCT-3′), which was synthesized as a DNA template using GeneArt Strings (Invitrogen) and then transcribed with a T7 RNA synthesis kit (New England Biolabs). PCS2-Cas9 (Addgene 47322), a gift from Alex Schier, was transcribed using a SP6 mMESSAGE mMACHINE kit (Ambion) and then co-injected with the guide RNA (gRNA) into one-cell embryos. Genomic DNA was Hotshot extracted from single 24 hpf embryos; 85 base pair flanking the cut site were PCR amplified (forward: 5′-TCTGTCCGTCTGCTCTCTTC-3′; reverse: 5′-AGGCTTTCTCCGTCACGTAG-3′) and subjected to high resolution melt curve analysis (HRMA) using Precision Melt Supermix (Bio-Rad). Mutations were confirmed by Sanger sequencing (Source Biosciences).

pERK/tERK Activity Mapping

Immunohistochemistry

Fish were fixed overnight at 4°C in 4% paraformaldehyde (PFA) and 4% sucrose in PBS; permeabilized 45 min in 0.05% trypsin-EDTA on ice; blocked 6 hr at room temperature (RT) in phosphate buffered saline plus 0.05% Triton (PBT) plus 2% normal goat serum, 1% BSA, and 1% DMSO; and then incubated over sequential nights at 4°C in primary antibodies (Cell Signaling Technology 4370 and 4696; 1:500) and secondary antibodies conjugated with Alexa fluorophores (Life Technologies; 1:200) in PBT plus 1% BSA and 1% DMSO.

Imaging

Larvae were mounted in 1.5% low melt agarose and imaged with a custom two-photon microscope (Bruker; Prairie View software) with a 20× water immersion objective (Olympus).

Z-Brain Registration and Mapping

Images were noise filtered using a custom MATLAB (The MathWorks) scripts and registered into Z-Brain using the Computational Morphometry Toolkit (http://www.nitrc.org/projects/cmtk/) with the command string: -a -w -r 0102 -l af -X 52 -C 8 -G 80 -R 3 -A "–accuracy 0.4 –auto-multi-levels 4" -W "–accuracy 1.6" -T 4. Registered images were prepared using a custom MATLAB/MIJ (http://bigwww.epfl.ch/sage/soft/mij/) script to downsize, blur, and adjust the maximum brightness of each stack to the top 0.1% of pixel intensities to preserve dynamic range. Activity maps were generated using MATLAB scripts (Randlett et al., 2015).

Behavioral Analysis

Sleep and Wake

Larvae were raised on a 14:10 hr light:dark cycle at 28.5°C. On 4 dpf, larvae were singly placed into a clear 96-square well plate (Whatman) filled with 650 μL of fish water (0.3 g/L Instant Ocean and 1 mg/L methylene blue [pH 7.0]). The plate was placed in a custom-modified Zebrabox (ViewPoint Life Sciences), and each well was monitored by automated tracking software and analyzed as in Rihel et al. (2010).

Spontaneous Coiling

Wild-type and mutant embryos from 30–36 hpf were kept in their chorion and placed in rows of 10 under a dissecting microscope. Coils per embryo were manually counted in three independent experiments.

Touch Sensitivity

36 and 52 hpf mutant and wild-type larvae were singly pipetted into a dish and then touched on the tail or head with a bent pipet tip. A response was recorded if the larva elicited an escape. One set was performed on larvae from a *pitpnc1a*$^{+/-}$ in-cross followed by genotyping.

In Vivo Drug Experiments

Larvae were dark reared at 28.5°C to 5 dpf and then exposed to the PI3K inhibitor LY294002 or the Akt inhibitor MK-2206 in 5 mL of water in six-well plates (n = 25) overnight, anesthetized with MS-222, and fixed with 4% PFA. For behavioral testing, drugs dissolved in DMSO were added to each well (final concentrations of 100 nM to 10 μM, 0.2% DMSO). Outliers (n = 1) were excluded by Grubb's test ($p < 0.01$). For IGFBP2 soaking, 10 dechorionated 34 hpf embryos were transferred into 100 μL of fish water with 0, 0.05, 0.1, 0.5, and 1 ng/μL of recombinant human IGFBP2 (PeproTech, Catalog no. 350-06B) in a 96-well plate overnight at 28.5°C.

Statistical Analysis

Behavioral data were analyzed for statistical significance using one-way ANOVA followed by Tukey's post hoc test ($\alpha = 0.05$). Interactions between genotype and drug were analyzed by two-way ANOVA ($\alpha = 0.05$). Touch sensitivity was analyzed by chi-square test.

Molecular and Biochemical Methods

See Supplemental Information for additional information.

SUPPLEMENTAL INFORMATION

Supplemental Information includes Supplemental Experimental Procedures, four figures, and three tables and can be found with this article online at https://doi.org/10.1016/j.celrep.2018.07.012.

ACKNOWLEDGMENTS

We thank the Cockcroft and Rihel labs for comments and technical support and Yoshiyuki Yamamoto for his initial zebrafish help. This work was supported by grants to S.C. from BBSRC (FS/15/73/31672) and BHF (BB/J005606/1), a UCL Excellence Grant, and ERC Starting Grant (282027) to J.R.

AUTHOR CONTRIBUTIONS

T.G.A., S.C., and J.R. conceived the experiments and wrote the manuscript. T.G.A. and N.J.B. performed biochemical experiments, and T.G.A., M.G., and J.R. performed behavior and imaging experiments.

DECLARATION OF INTERESTS

The authors declare no competing interests.

Received: May 2, 2018
Revised: June 21, 2018
Accepted: July 3, 2018
Published: August 7, 2018

REFERENCES

Barlow, I.L., and Rihel, J. (2017). Zebrafish sleep: from geneZZZ to neuronZZZ. Curr. Opin. Neurobiol. *44*, 65–71.

Chen, J., Alberts, I., and Li, X. (2014). Dysregulation of the IGF-I/PI3K/AKT/mTOR signaling pathway in autism spectrum disorders. Int. J. Dev. Neurosci. *35*, 35–41.

Cockcroft, S. (2012). The diverse functions of phosphatidylinositol transfer proteins. Curr. Top. Microbiol. Immunol. *362*, 185–208.

Duan, C., Ding, J., Li, Q., Tsai, W., and Pozios, K. (1999). Insulin-like growth factor binding protein 2 is a growth inhibitory protein conserved in zebrafish. Proc. Natl. Acad. Sci. USA *96*, 15274–15279.

Garner, K., Li, M., Ugwuanya, N., and Cockcroft, S. (2011). The phosphatidylinositol transfer protein RdgBβ binds 14-3-3 via its unstructured C-terminus, whereas its lipid-binding domain interacts with the integral membrane protein ATRAP (angiotensin II type I receptor-associated protein). Biochem. J. *439*, 97–111.

Greenawalt, D.M., Sieberts, S.K., Cornelis, M.C., Girman, C.J., Zhong, H., Yang, X., Guinney, J., Qi, L., and Hu, F.B. (2012). Integrating genetic association, genetics of gene expression, and single nucleotide polymorphism set analysis to identify susceptibility Loci for type 2 diabetes mellitus. Am. J. Epidemiol. *176*, 423–430.

Halberg, N., Sengelaub, C.A., Navrazhina, K., Molina, H., Uryu, K., and Tavazoie, S.F. (2016). PITPNC1 recruits RAB1B to the Golgi network to drive malignant secretion. Cancer Cell *29*, 339–353.

Hwang, W.Y., Fu, Y., Reyon, D., Maeder, M.L., Tsai, S.Q., Sander, J.D., Peterson, R.T., Yeh, J.-R.J., and Joung, J.K. (2013). Efficient genome editing in zebrafish using a CRISPR-Cas system. Nat. Biotechnol. *31*, 227–229.

Küry, S., Besnard, T., Ebstein, F., Khan, T.N., Gambin, T., Douglas, J., Bacino, C.A., Craigen, W.J., Sanders, S.J., Lehmann, A., et al. (2017). De novo disruption of the proteasome regulatory subunit psmd12 causes a syndromic neurodevelopmental disorder. Am. J. Hum. Genet *100*, 352–363.

Liu, J.Z., Erlich, Y., and Pickrell, J.K. (2017). Case-control association mapping by proxy using family history of disease. Nat. Genet. *49*, 325–331.

Pasquier, J., Cabau, C., Nguyen, T., Jouanno, E., Severac, D., Braasch, I., Journot, L., Pontarotti, P., Klopp, C., Postlethwait, J.H., et al. (2016). Gene evolution and gene expression after whole genome duplication in fish: the PhyloFish database. BMC Genomics *17*, 368.

Png, K.J., Halberg, N., Yoshida, M., and Tavazoie, S.F. (2011). A microRNA regulon that mediates endothelial recruitment and metastasis by cancer cells. Nature *481*, 190–194.

Randlett, O., Wee, C.L., Naumann, E.A., Nnaemeka, O., Schoppik, D., Fitzgerald, J.E., Portugues, R., Lacoste, A.M.B., Riegler, C., Engert, F., and Schier, A.F. (2015). Whole-brain activity mapping onto a zebrafish brain atlas. Nat. Methods *12*, 1039–1046.

Rihel, J., Prober, D.A., and Schier, A.F. (2010). Monitoring sleep and arousal in zebrafish. Methods Cell Biol. *100*, 281–294.

Takano, N., Owada, Y., Suzuki, R., Sakagami, H., Shimosegawa, T., and Kondo, H. (2003). Cloning and characterization of a novel variant (mM-rdgBbeta1) of mouse M-rdgBs, mammalian homologs of *Drosophila* retinal degeneration B gene proteins, and its mRNA localization in mouse brain in comparison with other M-rdgBs. J. Neurochem. *84*, 829–839.

Tilley, S.J., Skippen, A., Murray-Rust, J., Swigart, P.M., Stewart, A., Morgan, C.P., Cockcroft, S., and McDonald, N.Q. (2004). Structure-function analysis of human [corrected] phosphatidylinositol transfer protein alpha bound to phosphatidylinositol. Structure *12*, 317–326.

Zheng, W., Wang, H., Zeng, Z., Lin, J., Little, P.J., Srivastava, L.K., and Quirion, R. (2012). The possible role of the Akt signaling pathway in schizophrenia. Brain Res. *1470*, 145–158.

Cell Reports, Volume *24*

Supplemental Information

Pitpnc1a Regulates Zebrafish Sleep and Wake Behavior through Modulation of Insulin-like Growth Factor Signaling

Tim G. Ashlin, Nicholas J. Blunsom, Marcus Ghosh, Shamshad Cockcroft, and Jason Rihel

Supplementary Materials

Figure S1-related to Figure 1. Zebrafish have two orthologs of human PITPNC1

Figure S2-related to Figure 1. Zebrafish Pitpnc1a shares biochemical properties with human PITPNC1.

Figure S3-related to Figure 2. A CRISPR/Cas9 generated five base deletion of zebrafish *pitpnc1a* leads to a truncated protein lacking key functional residues.

Figure S4-related to Figure 4. IGFBP2 inhibits IGFR, *c-fos* expression, and brain size in *pitpnc1a*$^{-/-}$ animals.

Supplementary Table S1-related to Figure 2. Behavioral parameters of *pitpnc1a*$^{-/-}$ larvae.

Supplementary Table S2-related to Figure 3. Anti-inflammatory compounds with behavioral fingerprints that co-cluster with *pitpnc1a*$^{-/-}$ larvae.

Supplementary Table S3-related to Figure 3. MAP-Mapped brain regions and transgenic lines that overlap with up- and down- regulated pERK signals in *pitpnc1a*$^{-/-}$ larvae.

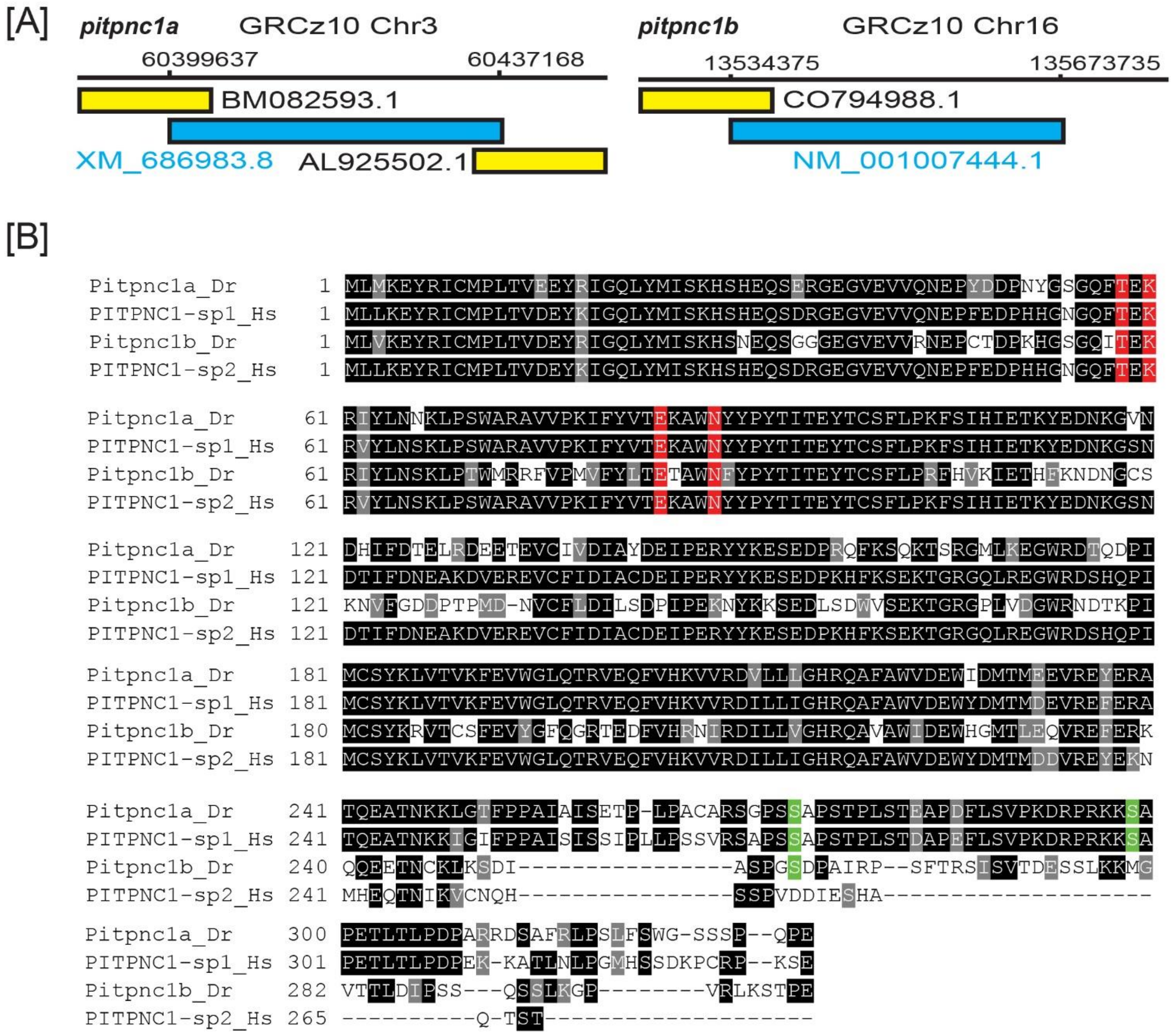

Figure S1-related to Figure 1

<u>Figure S1. Zebrafish have two orthologs of human PITPNC1, related to Figure 1</u> A) A schematic showing the EST sequences that were used to assemble *pitpnc1a* and *pitpnc1b* genes in the absence of annotated sequences. The EST XM_686983.8 was missing part of the expected 5' and 3' ends of the *pitpnc1a* transcript, including the expected start and stop codons. Alignments revealed partial ESTs BM082593.1 and AL925502.1 overlapped with the partial transcript and contained the full length ORF. The complete *pitpnc1b* sequence was assembled from the two ESTs CO794988.1 and NM_001007444.1. B) Alignments of zebrafish Pitpnc1a and Pitpnc1b with the human PITPNC1-sp1 and PITPNC1-sp2 protein sequences. The black background indicates amino acid residues that are identical and gray background indicates amino acid similarity. Key residues important for binding of the inositol ring of phosphatidylinositol (T59, K61, E86, and N90, mouse numbering) are highlighted in red and the phosphorylation sites that are important for 14-3-3 binding are highlighted in green.

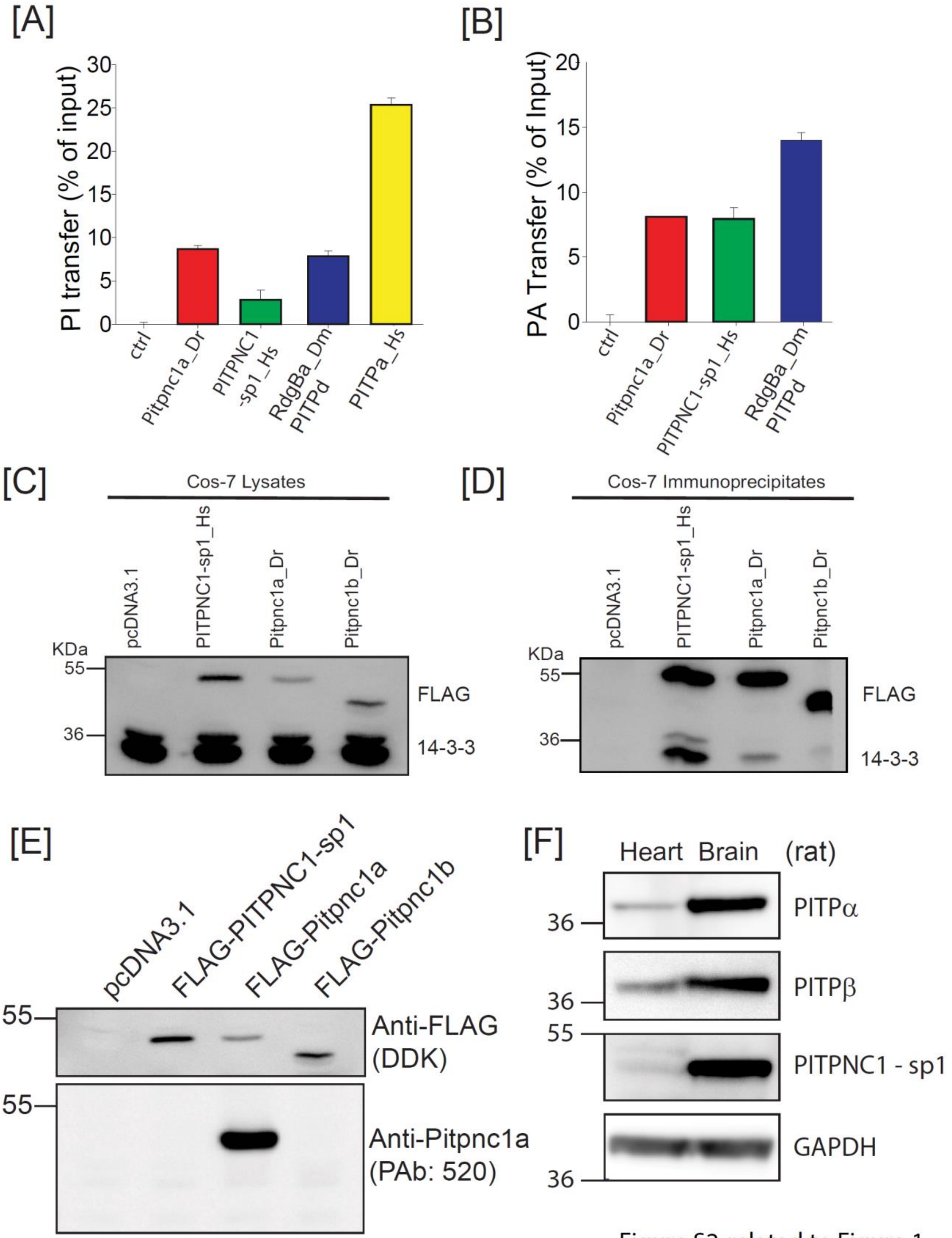

Figure S2-related to Figure 1

Figure S2. Zebrafish Pitpnc1a shares biochemical properties with human PITPNC1, related to Figure 1. A-B) Purified recombinant His-tagged zebrafish Pitpnc1a transfers both PI (A) and PA (B) *in vitro*, similar to human PITPNC1. The data is from a representative experiment done in duplicate; the error bars represent the range. C-D) FLAG-tagged PITPNC1-sp1_Hs, Pitpnc1a_Dr and Pitpnc1b_Dr constructs were electroporated into Cos-7 cells, immunoprecipiated against the FLAG-tags, and simultaneously probed by Western blot with antibodies against the Flag-tag and 14-3-3. Recombinant proteins and 14-3-3 (The 14-3-3 protein family comprises of 28-33 kDa proteins and the antibody detects all isoforms) were detectable in all lysates (C), but only the long form PITPNC1_Hs and Pitpnc1a_Dr were able to pull down 14-3-3 proteins (D). E) Flag-tagged Pitpnc1a and Pitpnc1b were expressed in Cos-7 cells by electroporation. Western blotting with a monoclonal anti-FLAG antibody serves as a positive control (top panel). The antibody recognizes only zebrafish Pitpnc1a. F) Western blot of the cytosolic fractions of rat brain and heart tissues probed with a cross-reacting anti-PITPNC1-sp1 antibody (RB59, see methods) strongly detects PITPNC1-sp1 in the rat brain and weakly in the heart. Antibodies to PITPα (Ab:674) and PITPβ (4A7) also detect these proteins in rat brain and heart.

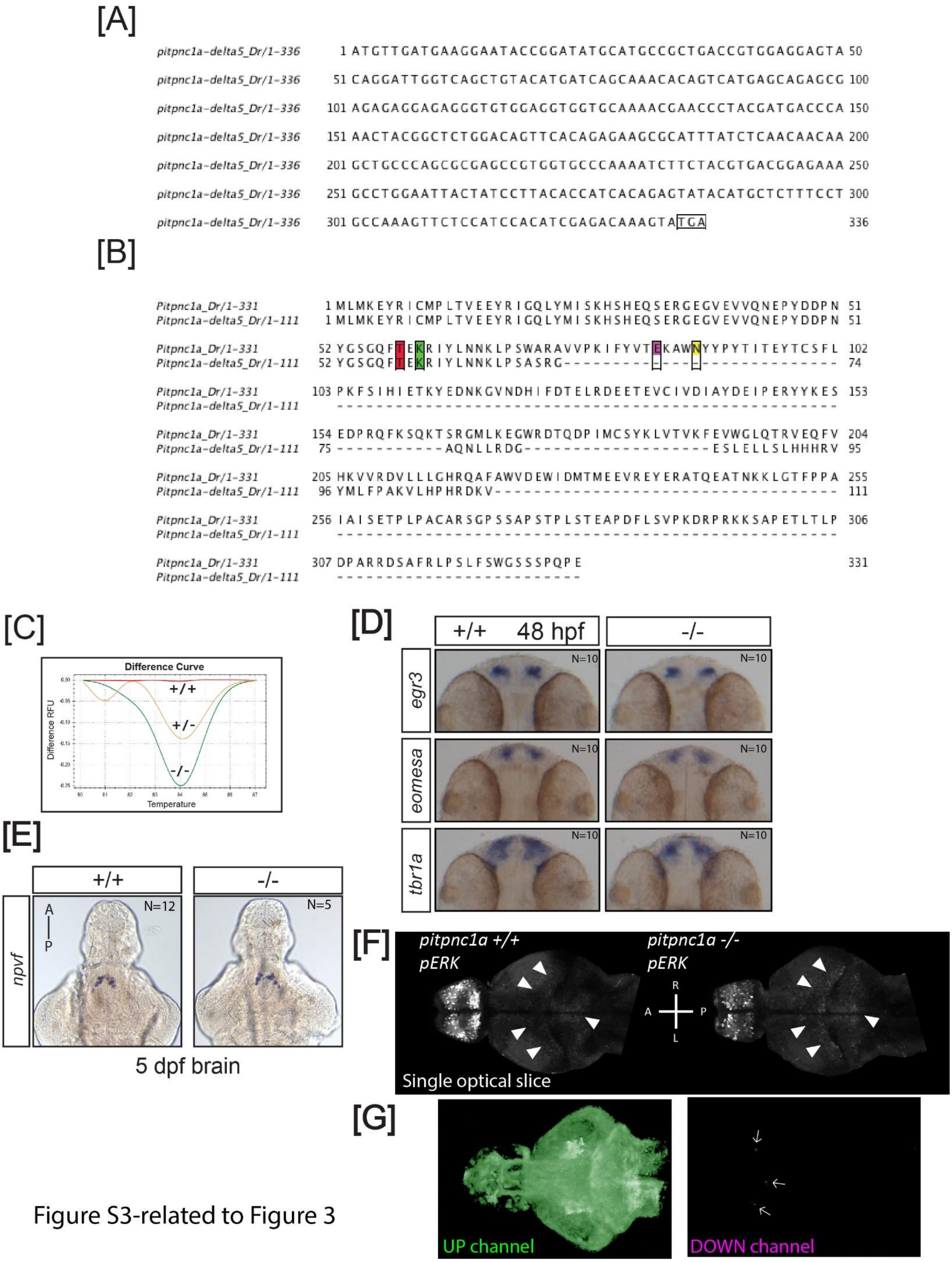

Figure S3-related to Figure 3

Figure S3. A CRISPR/Cas9 generated five base deletion of zebrafish *pitpnc1a* leads to a truncated protein lacking key functional residues, related to Figure 3. A) The *pitpnc1a* Δ5 allele leads to a truncated 336 bp open reading frame. B) Alignment of the full length Pitpnc1a and predicted truncated protein of the *pitpnc1a Δ5* allele. Critical amino acids for binding of the inositol ring of phosphatidylinositol (T59, K61, E86, and N90, mouse numbering) are highlighted, demonstrating the truncated protein lacks two of these critical residues.

C) DNA extracted from whole larvae and subjected to high resolution melt curve analysis (HRMA) is able to distinguish the wild type (+/+, red), heterozygous (+/-, yellow), and homozygous mutant (-/-, green) *pitpnc1a* genotypes. D) In situ hybridization revealed the expression of the dorsal forebrain markers *egr3*, *eomesa*, and *tbr1a* are unaffected in *pitpnc1a*$^{-/-}$ animals. Dorsal views; anterior to the top. E) In situ hybridization for an anterior hypothalamic marker, *npvf*, is unaffected in *pitpnc1a*$^{-/-}$ animals. Ventral views; anterior to the top. F) Representative optical pERK slices (plane 88 of the Z-brain) from wild type and mutant brains stained with pERK/tERK and linearly registered to the Z-Brain reference using the tERK channel. White arrowheads point to examples of areas with upregulated pERK in mutant brains. Images were normalized for intensity using the Stack Normalizer plugin https://imagej.nih.gov/ij/plugins/normalizer.html in Fiji. G) Single channels for the unthresholded maximum projections for the mutant upregulated (green, left) and downregulated (magenta, right) pERK signals.

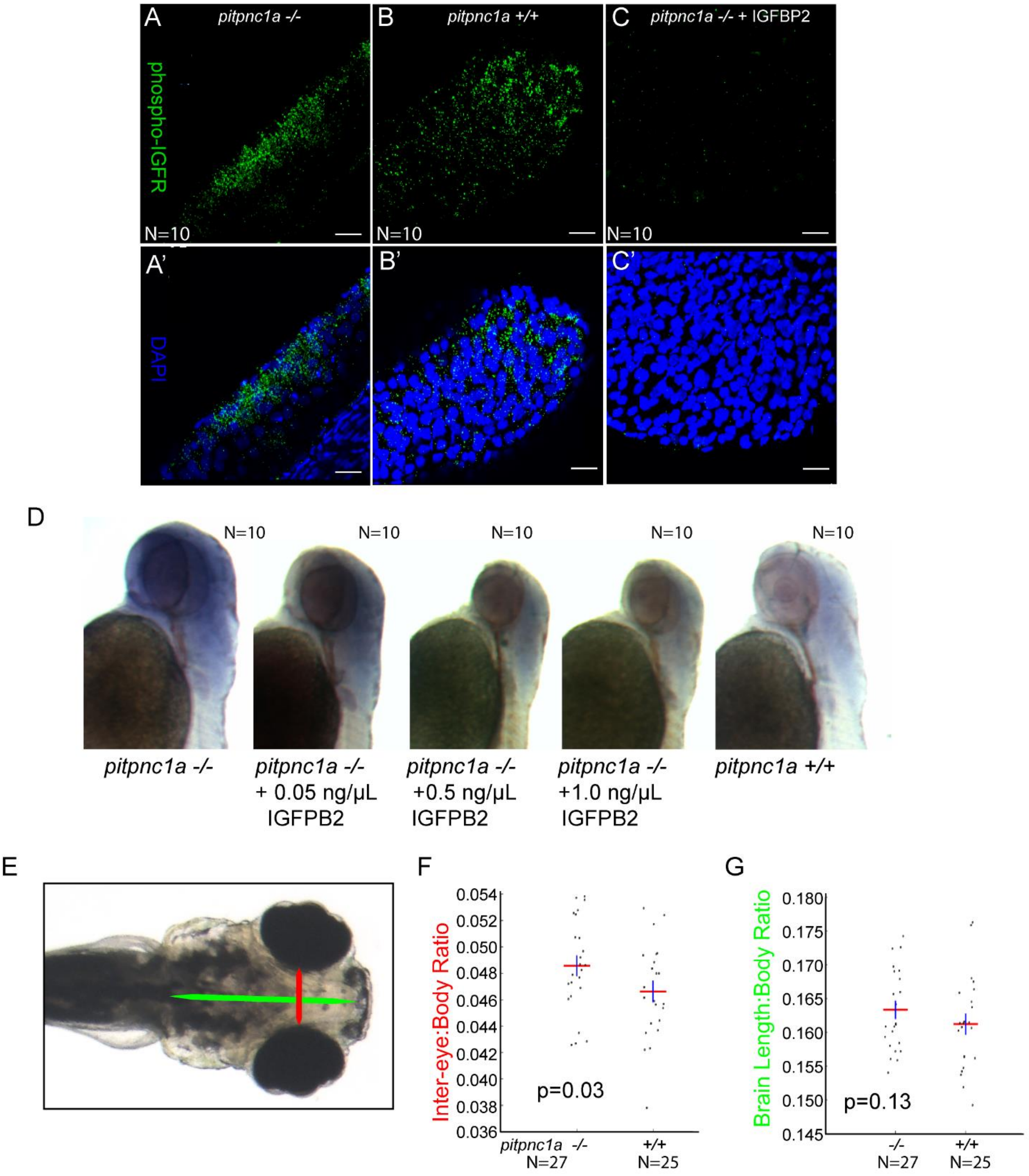

Figure S4-related to Fig 4

<u>Figure S4</u>. <u>IGFBP2 dampens IGF signaling in zebrafish larvae, related to Figure 4</u>. A-C') Confocal images of 48 hpf tailfins labeled with anti-pIGFR antibody (green) reveals numerous puncta in both *pitpnc1a* $^{-/-}$ (B, B') and *pitpnc1a* $^{+/+}$ (C, C') embryos, which are nearly eliminated after soaking in 1 ng/µL IGFBP2 (C, C'). D) IGFBP2 dose-dependently reduces brain *c-fos* levels and reduces growth in *pitpnc1a* $^{-/-}$ embryos. E-G) *pitpnc1a* $^{-/-}$ animals have slightly larger brains than *pitpnc1a*$^{+/+}$ larvae. E) Inter-ocular distance (red line) and brain length (green) were measured relative to total body length (not shown). F) Mutant brains are significantly (4.2%) larger than wild type (p=0.03, one-tailed t-test) as measured by inter-ocular distance G) and trending (1.3%) larger by length (p=0.13, one-tailed t-test). Scale bars, A-C', 20 µm.

Table 1. Behavioral Data

Sleep (min/hr)	**pitpnc1a +/+**<br>Mean +/- SEM	**pitpnc1a +/-**<br>Mean +/- SEM	**pitpnc1a -/-**<br>Mean +/- SEM	**ANOVA**
Day 6	8.3 ± 0.9*	6.0 ± 0.5	5.3 ± 0.5*	p=0.03
Night 6	22.3 ± 1.2	19.4 ± 0.7	19.0 ± 1.1	p=0.06
Sleep Bouts (#/hr)				
Day 6	2.7 ± 0.3*	2.0 ± 0.1*	1.8 ± 0.2*	p=0.01
Night 6	7.0 ± 0.2	6.8 ± 0.1	6.7 ± 0.2	p=0.65
Sleep Length (min/bout)				
Day 6	2.7 ± 0.1	2.4 ± 0.9	2.5 ± 0.2	p=0.10
Night 6	3.3 ± 0.2	2.9 ± 0.1	2.8 ± 0.2	p=0.09
Sleep Latency (min)				
Day 6	57.5 ± 18.7	61.8 ± 10.6	74.6 ± 19.5	p=0.76
Night 6	12.6 + 1.2	12.7 ± 0.8	14.6 ± 3.1	p=0.68
Average Activity (s/hr)				
Day 6	244.6 ± 12.1***	267.3 ± 6.8***	303.6 ±12.4***	p=0.001
Night 6	42.2 ± 2.2	44.5 ± 1.1	53.4 ± 3.8	p=0.002
Waking Activity (s/min)				
Day 6	4.4 ± 0.2***	4.8 ± 0.1***	5.3 ± 0.2***	p=0.002
Night 6	1.00 ± 0.04***	1.00 ± 0.02***	1.17 ± 0.07***	p=0.004

Table S1. Sleep-Wake Measurements on 6 dpf *pitpnc1a* mutants and their siblings, related to Figure 2. This table shows mean ± SEM of all behavioral parameters quantified in the primary screen during day and night for all three genotypes. Only the day and night waking activity measurements are strongly different between *pitpnc1a* $^{-/-}$ and their wild type and heterozygous siblings. The *pitpnc1a* mutants also have a modest reduction in daytime sleep compared to wild type, but not heterozygous, animals.

Regions UPREGULATED in *pitpnc1a* mutants

ROI name	Signal in ROI	Top Label	Signal
**Diencephalon - Otpb Cluster 1**	**25981.0186**	**Anti-TH**	**2.5212**
Rhombencephalon - Cerebellar Neuropil 1	25772.6359	Anti-Zrf2	4.3018
Rhombencephalon - Otpb Cluster 1	25277.9683	Vglut2a-GFP	1.8137
Diencephalon - Hypothalamus 6.7FRhcrtR-Gal4 cluster 2	25024.2484	Elavl3-H2BRFP	2.1732
**Diencephalon - Dopaminergic Cluster 4/5 - posterior tuberculum and**	**24980.4805**	**Anti-TH**	**2.1162**
Rhombencephalon - RoL-R1	24697.8915	SpinalBackfills	4.0324
**Diencephalon - Hypothalamus Hcrt Neurons**	**24444.5146**	**Hcrt-RFP**	**6.4504**
**Diencephalon - Anterior group of the posterior tubercular vmat2 neu**	**24422.164**	**Hcrt-RFP**	**3.7191**
**Diencephalon - Dopaminergic Cluster 3 - hypothalamus**	**24407.3153**	**Hcrt-RFP**	**4.1452**
Rhombencephalon - Oculomotor Nucleus nIV	24391.4688	Isl1-GFP	2.6829
Diencephalon - Oxtl Cluster 3	24326.3727	Oxtl-GFP	2.6515
Rhombencephalon - Neuropil Region 6	24130.3709	Isl2bGal4-uasDendra	1.1822
**Diencephalon - Hypothalamus s1181t Cluster**	**23800.4091**	**Qrfp-GFP**	**10.5892**
Rhombencephalon - Gad1b Cluster 3	23632.5067	EtVmat2-GFP	2.3026
**Diencephalon - Hypothalamus Vglut2 Cluster 2**	**23536.9321**	**Vglut2a-GFP**	**2.5232**
**Diencephalon - Dopaminergic Cluster 2 - posterior tuberculum**	**23485.6244**	**Qrfp-GFP**	**3.3125**
Diencephalon - Hypothalamus 6.7FRhcrtR-Gal4 cluster 1	23337.569	6.7FRhcrtR-Gal4-uas	3.5525
Rhombencephalon - Valvula Cerebelli	23328.3832	Ptf1aGal4-uasKaede	1.8429
Mesencephalon - Torus Longitudinalis	23166.4114	Anti-Zrf1(GFAP)	2.1295
Rhombencephalon - RoM1	23108.3556	SpinalBackfills	1.8507
Mesencephalon - Ptf1a Cluster	22919.6422	Ptf1aGal4-uasKaede	1.9644
Diencephalon - Oxtl Cluster 4 - sparse in hypothalamus	22694.3046	EtVmat2-GFP	1.2291
Diencephalon - Migrated Posterior Tubercular Area (M2)	22472.4579	Vglut2a-GFP	1.4983
Diencephalon - Isl1 cluster 3	22368.3456	Elavl3-H2BRFP	2.0971
Rhombencephalon - Oxtl Cluster 1 Sparse	22030.9352	Gad1b-GFP	1.3576
Rhombencephalon - Interpeduncular Nucleus	22016.2018	Anti-Zrf1(GFAP)	1.4155
Rhombencephalon - Locus Coreuleus	21928.3881	EtVmat2-GFP	2.1319
Mesencephalon - NucMLF (nucleus of the medial longitudinal fascicle)	21688.0025	SpinalBackfills	4.7695
Rhombencephalon - Cerebelluar-Vglut2 enriched areas	21641.3931	Vglut2a-GFP	2.1027
Diencephalon - Hypothalamus Gad1b Cluster 2	21586.3209	Qrfp-GFP	2.6368
Diencephalon - Dopaminergic Cluster 6 - hypothalamus	21585.2101	Anti-Zrf1(GFAP)	1.3578
Rhombencephalon - Lobus caudalis cerebelli	21557.0639	Anti-Zn1	1.9184
Mesencephalon - Otpb Cluster	20895.8368	Isl1-GFP	2.1006
Rhombencephalon - Otpb Cluster 2 - locus coeruleus	20874.9172	EtVmat2-GFP	2.9075
Rhombencephalon - Raphe - Superior	20781.7916	EtVmat2-GFP	2.6177
Mesencephalon - Vmat2 cluster of paraventricular organ	20657.8203	EtVmat2-GFP	2.0563
Rhombencephalon - RoM2	20329.8217	SpinalBackfills	3.9807
Rhombencephalon - Gad1b Cluster 17	20272.9659	Gad1b-GFP	1.4565
Mesencephalon - Oculomotor Nucleus nIII	20207.035	Isl1-GFP	1.9912
Rhombencephalon - Gad1b Cluster 2	20183.2407	Gad1b-GFP	1.2934
Diencephalon - Oxtl Cluster 5	20178.0336	Oxtl-GFP	1.456
Rhombencephalon - 6.7FDhcrtR-Gal4 Stripe 4	20023.4785	6.7FRhcrtR-Gal4-uas	2.4838
Rhombencephalon - Cerebellum	19723.509	Ptf1aGal4-uasKaede	3.5764
Mesencephalon - Sparse 6.7FRhcrtR cluster	19695.47	6.7FRhcrtR-Gal4-uas	1.6002
Mesencephalon - Vglut2 cluster 1	19655.5664	Vglut2a-GFP	2.2592
Rhombencephalon - 6.7FDhcrtR-Gal4 Stripe 3	19617.8059	6.7FRhcrtR-Gal4-uas	2.1304
Diencephalon - Otpb Cluster 4	19520.048	Qrfp-GFP	2.8398
Mesencephalon - Oxtl Cluster Sparse	19519.7829	Anti-Zrf1(GFAP)	1.703
Diencephalon - Posterior Tuberculum	19417.4002	Qrfp-GFP	2.3476
Rhombencephalon - Corpus Cerebelli	19340.7978	Ptf1aGal4-uasKaede	2.5346
Diencephalon - Olig2 Band	19308.3995	Olig2-GFP	2.0385
Diencephalon - Medial vglut2 cluster	19140.9075	Vglut2a-GFP	1.6591
Rhombencephalon - Rhombomere 1	19113.368	Ptf1aGal4-uasKaede	2.2558
Diencephalon - Dorsal Thalamus	19062.259	Elavl3-H2BRFP	1.1639
Diencephalon - Retinal Arborization Field 2 (AF2- Approximate Location	19007.1551	Isl2bGal4-uasDendra	11.7149
Mesencephalon - Tegmentum	18843.8642	Elavl3-H2BRFP	1.1758
Rhombencephalon - Gad1b Cluster 7	18827.3396	Ptf1aGal4-uasKaede	2.6501
Diencephalon - Dopaminergic Cluster 1 - ventral thalamic and periventr	18810.4778	Oxtl-GFP	2.8134
Diencephalon - Hypothalamus Gad1b Cluster 1	18709.752	Gad1b-GFP	1.9097
Mesencephalon - Vmat2 cluster2	18664.0675	Elavl3-H2BRFP	1.7239
Diencephalon - Retinal Arborization Field 3 (AF3)	18483.294	Isl2bGal4-uasDendra	4.2447
Diencephalon - Dopaminergic Cluster 7 - Caudal Hypothalamus	18389.0041	Anti-TH	1.3741
Rhombencephalon - RoL3	18322.6043	SpinalBackfills	3.8889
Diencephalon - Oxtl Cluster 2	18307.4762	Isl2bGal4-uasDendra	2.8835
Diencephalon - Ventral Thalamus	17759.7376	Gad1b-GFP	2.226
Diencephalon - Hypothalamus Qrfp neuron cluster	17530.7811	Qrfp-GFP	13.2006

Rhombencephalon - Cerebellum Gad1b Enriched Areas	17521.9014	Ptf1aGal4-uasKaede	4.1139
Rhombencephalon - Eminentia Granularis	17499.9396	Ptf1aGal4-uasKaede	2.7132
Rhombencephalon - Spiral Fiber Neuron Anterior cluster	17238.6357	6.7FRhcrtR-Gal4-uas	5.2927
Rhombencephalon - RoL2	16980.4361	Anti-Zrf2	1.9921
Rhombencephalon - Posterior Cluster of nV Trigeminal Motorneurons	16410.2899	Isl1-GFP	3.052
Rhombencephalon - RoM3	16289.9706	SpinalBackfills	5.3922
Diencephalon - Pretectal dopaminergic cluster	16280.5438	EtVmat2-GFP	2.0998
Rhombencephalon - Spiral Fiber Neuron Posterior cluster	16252.4079	6.7FRhcrtR-Gal4-uas	4.2119
Diencephalon - Anterior pretectum cluster of vmat2 Neurons	16248.0535	EtVmat2-GFP	2.2833
Rhombencephalon - Olig2 enriched areas in cerebellum	16125.4539	Olig2-GFP	2.2364
Diencephalon - Pretectum	15567.4039	EtVmat2-GFP	1.6205
Rhombencephalon - Glyt2 Cluster 8	15432.0625	SpinalBackfills	1.5654
Rhombencephalon - Otpb Cluster 3	15293.4305	S1181tGal4-uasKaed	1.3101
Rhombencephalon - Glyt2 Cluster 7	15277.5816	SpinalBackfills	1.6773
Rhombencephalon - MiR2	15258.2692	SpinalBackfills	3.9808
Diencephalon - Retinal Arborization Field 4 (AF4)	15138.4915	Isl2bGal4-uasDendra	2.6436
Rhombencephalon - RoV3	15064.6138	SpinalBackfills	2.0345
Mesencephalon - Retinal Arborization Field 9 (AF9)	14977.7142	Anti-Zrf2	2.5295
Rhombencephalon - Vmat2 Cluster 1	14959.5042	EtVmat2-GFP	2.576
Rhombencephalon - Vmat2 Cluster 2	14828.3563	EtVmat2-GFP	2.4008
Rhombencephalon - Anterior Cluster of nV Trigeminal Motorneurons	14795.0733	Isl1-GFP	1.905
Rhombencephalon - Otpb Cluster 4	14765.1643	Anti-Zn1	1.2819
Diencephalon - Pretectal Gad1b Cluster	14085.8988	EtVmat2-GFP	2.4021
Rhombencephalon - Gad1b Cluster 15	14032.8187	Elavl3-H2BRFP	1.7698
Diencephalon - Isl1 cluster 2	13900.8569	Elavl3-H2BRFP	2.4804
Rhombencephalon - Isl1 Cluster 2	13731.7645	Isl1-GFP	1.637
Rhombencephalon - Gad1b Cluster 16	13655.763	Isl1-GFP	1.5329
Diencephalon - Isl1 cluster 1	13567.142	Elavl3-H2BRFP	2.1193
Rhombencephalon - Vglut2 cluster 1	13491.4218	Oxtl-GFP	0.96964
Diencephalon - Oxtl Cluster 1 in Preoptic Area	13280.4796	Oxtl-GFP	6.1066
Rhombencephalon - Gad1b Cluster 8	13176.4207	Elavl3-H2BRFP	1.1957
Rhombencephalon - Vglut2 cluster 2	13120.4868	Vglut2a-GFP	3.2905
Rhombencephalon - S1181t Cluster	13025.7548	Vglut2a-GFP	2.7142
Telencephalon - Anterior Commisure	13002.4827	Anti-Zrf2	4.9087
Rhombencephalon - MiR1	12768.2045	SpinalBackfills	4.3695
Mesencephalon - Isl1 cluster of the mesencephalic region	12710.5215	Elavl3-H2BRFP	1.5298
Mesencephalon - Medial Tectal Band	12628.6121	Gad1b-GFP	2.2948
Rhombencephalon - Rhombomere 2	12488.7939	Anti-GlyR	1.5336
Rhombencephalon - Glyt2 Cluster 14	12428.2177	S1181tGal4-uasKaed	2.6781
Rhombencephalon - Glyt2 Cluster 2	12085.8795	EtVmat2-GFP	1.7867
Diencephalon - Migrated Area of the Pretectum (M1)	11991.6766	Anti-Zrf2	1.9103
Rhombencephalon - Glyt2 Cluster 10	11965.2451	Anti-GlyR	1.6519
Rhombencephalon - Glyt2 Cluster 1	11581.9456	Glyt2-GFP	1.7723
Diencephalon - Rostral Hypothalamus	11512.9118	Qrfp-GFP	4.6131
Rhombencephalon - Oxtl Cluster 2 Near MC axon cap	11119.678	Anti-Zn1	1.5114
Diencephalon -	10916.8181	Elavl3-H2BRFP	1.6346
Rhombencephalon - MiM1	10603.0699	SpinalBackfills	5.774
Rhombencephalon - Neuropil Region 5	10387.5568	Anti-GlyR	2.567
Rhombencephalon - Rhombomere 3	10202.2821	Anti-GlyR	1.8715
Rhombencephalon - Vmat2 Cluster 5	10129.1022	EtVmat2-GFP	2.1134
Diencephalon - Intermediate Hypothalamus	10039.5219	Anti-tERK	1.9929
Rhombencephalon - Gad1b Cluster 18	9819.5775	Gad1b-GFP	2.5535
Mesencephalon - Tectum Stratum Periventriculare	9773.364	Elavl3-GCaMP5G	3.1547
Diencephalon - Otpb Cluster 3	9635.6113	Vglut2a-GFP	2.1056
Rhombencephalon - MiV1	9466.2577	SpinalBackfills	3.9809
Rhombencephalon - Gad1b Cluster 1	9436.1355	Gad1b-GFP	1.5393
Rhombencephalon - Isl1 Cluster 1	9304.3085	Isl1-GFP	2.2877
Diencephalon - Hypothalamus Vglut2 Cluster 1	9300.2474	Vglut2a-GFP	1.9122
Rhombencephalon - Spinal Backfill Vestibular Population	9275.8864	SpinalBackfills	4.302
Diencephalon - Otpb Cluster 2	9253.0628	Oxtl-GFP	9.0204
Rhombencephalon -	9103.588	Anti-GlyR	2.4467
Rhombencephalon - Neuropil Region 4	9018.7376	Anti-Znp1(Synaptota	4.2576
Mesencephalon -	8977.596	Isl2bGal4-uasDendra	4.4506
Rhombencephalon - Olig2 Cluster	8815.4639	Olig2-GFP	2.8556
Diencephalon - Retinal Arborization Field 5 (AF5)	8717.035	EtVmat2-GFP	3.6528
Rhombencephalon - Gad1b Cluster 19	8705.9207	Anti-GlyR	3.159
Diencephalon - Diffuse Nucleus of the Intermediate Hypothalamus	8446.8826	Anti-5HT	2.1637
Diencephalon - Olig2 Band 2	8399.2527	Gad1b-GFP	1.1791

Rhombencephalon - Glyt2 Cluster 9	8342.509	Anti-Znp1(Synaptota	1.766
Rhombencephalon - Gad1b Cluster 9	8218.1532	EtVmat2-GFP	1.4168
Rhombencephalon - Vmat2 Cluster 4	8188.5054	EtVmat2-GFP	2.6607
Rhombencephalon - Ptf1a Cluster 1	8118.8909	Ptf1aGal4-uasKaede	1.6627
Telencephalon - Subpallial Vglut2 Cluster	7846.0808	Vglut2a-GFP	4.7758
Diencephalon - Right Habenula Vglut2 Cluster	7748.972	Vglut2a-GFP	5.4079
Diencephalon - Preoptic Area	7726.9329	Oxtl-GFP	2.5649
Rhombencephalon - MiV2	7578.3955	SpinalBackfills	4.0993
Rhombencephalon - Rhombomere 4	7257.3402	Anti-GlyR	2.4306
Diencephalon - Preoptic area posterior dopaminergic cluster	7187.3107	Elavl3-H2BRFP	2.8357
Rhombencephalon - 6.7FDhcrtR-Gal4 Cluster 3	7139.9224	Elavl3-H2BRFP	2.028
Mesencephalon - Torus Semicircularis	6915.9372	Anti-Znp1(Synaptota	2.1369
Rhombencephalon - Mauthner Cell Axon Cap	6793.16	6.7FRhcrtR-Gal4-uas	4.7516
Rhombencephalon - MiD2	6557.1018	SpinalBackfills	5.1494
Rhombencephalon - 6.7FDhcrtR-Gal4 Cluster 1	6507.8588	6.7FRhcrtR-Gal4-uas	2.136
Diencephalon - Caudal Hypothalamus	6187.7406	Anti-GlyR	2.0664
Rhombencephalon - Rhombomere 5	6170.0327	Anti-GlyR	2.2369
Diencephalon - Hypothalamus - Intermediate Hypothalamus Neural Clu	6140.1942	EtVmat2-GFP	1.4936
Diencephalon - Hypothalamus Olig2 cluster 2	5967.6493	Vglut2a-GFP	2.1072
Rhombencephalon - Gad1b Stripe 3	5723.338	Elavl3-H2BRFP	1.3249
Rhombencephalon - Gad1b Cluster 6	5529.5194	S1181tGal4-uasKaed	1.7194
Rhombencephalon - Glyt2 Cluster 13	5508.4537	Anti-Znp1(Synaptota	3.6382
Rhombencephalon - Mauthner	5495.4979	SpinalBackfills	5.1721
Rhombencephalon - VII Facial Motor and octavolateralis efferent neuro	5429.2693	Isl1-GFP	2.048
Rhombencephalon - Medial Vestibular Nucleus	5362.1351	6.7FRhcrtR-Gal4-uas	2.5527
Rhombencephalon - Glyt2 Stripe 3	5361.9914	Elavl3-H2BRFP	1.5001
Rhombencephalon - Gad1b Cluster 13	5243.6938	6.7FRhcrtR-Gal4-uas	2.4967
Rhombencephalon - Tangential Vestibular Nucleus	5233.631	6.7FRhcrtR-Gal4-uas	6.3976
Rhombencephalon - Glyt2 Cluster 3	5189.2915	Glyt2-GFP	2.1791
Rhombencephalon - Rhombomere 6	5170.1871	Vglut2a-GFP	3.2099
Rhombencephalon - Gad1b Cluster 4	5067.4164	Elavl3-H2BRFP	1.4278
Ganglia - Lateral Line Neuromast OC1	5054.0315	Anti-Zrf1(GFAP)	3.9653
Rhombencephalon - Olig2 Stripe	5005.9219	EtVmat2-GFP	1.6519
Rhombencephalon - Gad1b Cluster 5	4909.4778	Elavl3-H2BRFP	1.5384
Diencephalon - Retinal Arborization Field 6 (AF6)	4781.3851	Isl2bGal4-uasDendra	8.1399
Rhombencephalon - 6.7FDhcrtR-Gal4 Cluster 2 Sparse	4778.3954	Elavl3-H2BRFP	1.5556
Telencephalon - Isl1 cluster 1	4755.3119	Gad1b-GFP	2.7935
Telencephalon - Isl1 cluster 2	4641.2773	Gad1b-GFP	2.1503
Rhombencephalon - Gad1b Cluster 10	4571.3911	SpinalBackfills	2.2392
Rhombencephalon - Ventrolateral population of serotonergic neurons	4333.667	Anti-GlyR	1.6709
Rhombencephalon - Raphe - Inferior	4280.5882	Pet1-GFP	2.7938
Rhombencephalon - Vglut2 Stripe 1	4166.3896	Vglut2a-GFP	6.7061
Rhombencephalon - Gad1b Stripe 1	4156.8606	Gad1b-GFP	1.8978
Diencephalon - Habenula	4101.0755	Vglut2a-GFP	5.4536
Rhombencephalon - VII' Facial Motor and octavolateralis efferent neuro	4031.4279	Vglut2a-GFP	1.7762
Rhombencephalon - Vglut2 cluster 3	3878.5315	EtVmat2-GFP	1.3907
Telencephalon - Olig2 Cluster	3872.6523	Gad1b-GFP	2.6034
Mesencephalon - Retinal Arborization Field 8 (AF8)	3866.7417	EtVmat2-GFP	2.9323
Diencephalon - Pineal Vmat2 cluster	3663.9802	EtVmat2-GFP	5.0057
Rhombencephalon - 6.7FDhcrtR-Gal4 Stripe 2	3612.9157	6.7FRhcrtR-Gal4-uas	2.408
Telencephalon - Subpallium	3576.9327	Gad1b-GFP	3.2122
Rhombencephalon - Vglut2 Stripe 3	3532.7284	Elavl3-H2BRFP	1.4873
Rhombencephalon - Vglut2 Stripe 4	3381.3756	Vglut2a-GFP	1.6588
Rhombencephalon - Glyt2 Cluster 11	3321.0078	6.7FRhcrtR-Gal4-uas	2.656
Mesencephalon - Tecum Neuropil	3318.6878	Isl2bGal4-uasDendra	7.6604
Rhombencephalon - Glyt2 Cluster 6	3145.5263	Elavl3-H2BRFP	1.5583
Rhombencephalon - Otpb Cluster 5	3097.0262	S1181tGal4-uasKaed	2.066
Telencephalon - Subpallial Otpb Cluster 2	2901.4851	Gad1b-GFP	2.3515
Rhombencephalon - Isl1 Cluster 3	2826.002	Vglut2a-GFP	1.4495
Rhombencephalon - Glyt2 Cluster 5	2767.0874	Elavl3-H2BRFP	1.6957
Ganglia - Statoacoustic Ganglion	2576.675	Elavl3-H2BRFP	2.8031
Rhombencephalon - Vmat2 Cluster 3	2416.4021	6.7FRhcrtR-Gal4-uas	2.7124
Rhombencephalon - Gad1b Cluster 12	2316.9806	SpinalBackfills	2.8084
Rhombencephalon - Glyt2 Cluster 12	2315.2386	6.7FRhcrtR-Gal4-uas	2.9278
Telencephalon -	2238.306	Anti-Zrf2	3.5627
Telencephalon - Telencephalic Migrated Area 4 (M4)	2209.1071	Anti-Zrf2	3.6968
Rhombencephalon - Vglut2 Stripe 2	2196.7309	Elavl3-H2BRFP	1.6476
Diencephalon - Left Habenula Vglut2 Cluster	2190.1366	Vglut2a-GFP	5.1551

Telencephalon - Subpallial Gad1b cluster	2175.3643	Gad1b-GFP	4.2321
Rhombencephalon - MiD3	2162.7295	SpinalBackfills	4.6403
Diencephalon - Hypothalamus Vglut2 Cluster 5	2066.5736	Vglut2a-GFP	1.5754
Diencephalon - Pineal	2020.9022	Anti-TH	4.7874
Diencephalon - Preoptic Otpb Cluster	1997.6182	Anti-Zrf2	3.1383
Rhombencephalon - Qrfp neuron cluster sparse	1927.5861	Vglut2a-GFP	5.7229
Rhombencephalon - Vmat2 Stripe1	1647.006	Elavl3-H2BRFP	3.0626
Telencephalon - Vglut2 rind	1640.981	Vglut2a-GFP	3.6075
Rhombencephalon - 6.7FDhcrtR-Gal4 Cluster 4	1567.8025	6.7FRhcrtR-Gal4-uas	2.88
Diencephalon - Eminentia Thalami	1543.9245	Vglut2a-GFP	5.2082
Rhombencephalon - 6.7FDhcrtR-Gal4 Stripe 1	1510.3111	Gad1b-GFP	3.7183
Diencephalon - Hypothalamus Vglut2 Cluster 3	1411.1358	Anti-tERK	2.5902
Rhombencephalon - MiT	1326.4305	Anti-Znp1(Synaptota	1.5978
Telencephalon - Olfactory Bulb	1318.1344	Anti-Zrf1(GFAP)	2.2991
Rhombencephalon - Ptf1a Stripe	1304.9609	Gad1b-GFP	3.0223
Telencephalon - S1181t Cluster	1288.9228	Gad1b-GFP	1.9741
Rhombencephalon - Glyt2 Cluster 4	1207.5299	Glyt2-GFP	1.7452
Rhombencephalon - 6.7FDhcrtR-Gal4 Cluster 5	1196.9757	6.7FRhcrtR-Gal4-uas	2.5026
Ganglia - Lateral Line Neuromast SO3	1093.2377	Anti-Zrf1(GFAP)	5.0817
Diencephalon - Hypothalamus - Caudal Hypothalamus Neural Cluster	1037.0168	Anti-GlyR	2.1094
Telencephalon - Pallium	1026.6531	Vglut2a-GFP	3.2722
Diencephalon - Hypothalamus Gad1b Cluster 3 Sparse	1023.2356	Anti-GlyR	3.0996
Diencephalon - Retinal Arborization Field 1 (AF1- Approximate Location	950.8348	Isl2bGal4-uasDendra	3.3186
Telencephalon - Postoptic Commissure	924.856	Anti-Zrf2	4.2737
Rhombencephalon - Neuropil Region 2	910.6231	Anti-Znp1(Synaptota	4.0571
Rhombencephalon - Neuropil Region 3	910.5548	Anti-GlyR	2.98
Diencephalon - Hypothalamus Vglut2 Cluster 6	677.2467	Anti-tERK	2.6876
Diencephalon - Postoptic Commissure	594.5268	Qrfp-GFP	4.2828
Rhombencephalon - Rhombomere 7	585.4897	Gad1b-GFP	4.5624
Rhombencephalon - Gad1b Cluster 11	570.2383	Elavl3-H2BRFP	1.7336
Telencephalon - Optic Commissure	547.3087	Isl2bGal4-uasDendra	23.5869
Rhombencephalon - Glyt2 Stripe 2	534.1997	Elavl3-H2BRFP	1.6053
Diencephalon - Anterior preoptic dopaminergic cluster	491.3548	Gad1b-GFP	3.6267
Rhombencephalon - Gad1b Cluster 14	304.9341	6.7FRhcrtR-Gal4-uas	2.4987
Rhombencephalon - Glyt2 Stripe 1	294.9262	Elavl3-H2BRFP	1.7704
Diencephalon - Preoptic area Vglut2 cluster	271.119	Elavl3-H2BRFP	2.2216
Rhombencephalon - Vmat2 Stripe2	239.9459	6.7FRhcrtR-Gal4-uas	1.7529
Rhombencephalon - X Vagus motorneuron cluster	154.2477	Elavl3-H2BRFP	1.9454
Rhombencephalon - Otpb Cluster 6	131.0848	Elavl3-H2BRFP	1.9067
Ganglia - Lateral Line Neuromast SO2	109.4393	Otpb.A-Gal4-UAS-GC	3.0218
Ganglia - Facial Sensory Ganglion	105.8146	Elavl3-H2BRFP	3.0389
Telencephalon - Vmat2 cluster	103.7865	Vglut2a-GFP	2.378
Ganglia - Anterior Lateral Line Ganglion	78.0785	Elavl3-H2BRFP	4.4
Ganglia - Olfactory Epithelium	61.0479	Elavl3-H2BRFP	4.6833
Ganglia - Lateral Line Neuromast N	59.6494	Anti-GlyR	NaN
Ganglia - Trigeminal Ganglion	58.9868	Isl2bGal4-uasDendra	3.2657
Mesencephalon - Retinal Arborization Field 7 (AF7)	42.9445	Isl2bGal4-uasDendra	5.8098
Spinal Cord - 6.7FDhcrtR-Gal4 Stripe	33.9127	Elavl3-H2BRFP	2.9276
Rhombencephalon - Gad1b Stripe 2	33.9108	Elavl3-H2BRFP	1.7682
Diencephalon - Hypothamic VentroLateral VMAT cluster	19.0024	Elavl3-H2BRFP	5.0399
Rhombencephalon - Vglut2 cluster 4	12.8417	EtVmat2-GFP	6.3303
Telencephalon - Olfactory bulb dopaminergic neuron areas	8.0333	Gad1b-GFP	2.8758
Rhombencephalon - Caudal Ventral Cluster Labelled by Spinal Backfills	5.6987	SpinalBackfills	2.5794

Regions DOWNREGULATED in *pitpnc1a* mutants

ROI name	Signal in ROI	Top Label	Signal
Telencephalon - Pallium	5.5763	Anti-tERK	3.7072
Telencephalon - Vglut2 rind	4.8038	Vglut2a-GFP	4.3117
Telencephalon -	2.7383	Anti-Zrf2	5.7175
Rhombencephalon - Rhombomere 7	0.11117	Pet1-GFP	5.2942
Rhombencephalon -	0.04976	Pet1-GFP	6.3857

Table S2. MAP-Mapped brain regions and transgenic lines that overlap with up- and down-regulated pERK signals in *pitpnc1a*$^{-/-}$ larvae, related to Figure 3. Differential pERK signal in *pitpnc1a*$^{-/-}$ versus wild type larvae were morphed into the Z-brain atlas to identify annotated brain regions and the most correlated, overlapping transgenic lines. As Z-brain uses overlapping identifiers to name brain sub-regions, some areas and transgenes are represented multiple times.

Supplementary Table 3 - Enriched Drug Classes that Cluster with *pitpnc1a* mutant phenotypes

Rank	Name	Correlation
**PDE Inhibitor**		
1	Skf 94836	0.9353
6	Fosfosal	0.8754
7	Propentofylline	0.8733
24	Ibudilast	0.8004
33	Papaverine	0.7479
36	Ro20-1724	0.7377
**NSAID**		
16	Fenoprofen	0.8359
20	Aspirin	0.8243
26	Bufexamac	0.7931
**Glucocorticoids**		
4	Flunisolide	0.8953
12	Clobetasol	0.8489
30	Desoxycorticosterone	0.7541
32	Betamethasone	0.7509
34	Flumethasone	0.7443
42	Medrysone	0.7184
48	Hydrocortisone	0.7103
**Other Anti-inflammatory**		
5	Aminophenazone	0.8782
8	Capsazepine	0.8600
17	Nicotine Ditartrate	0.8321
25	(-)-Nicotine	0.7980
29	Theaflavin	0.7567
37	Diphenylpyraline	0.7283
38	Valproate	0.7264
**NMDA Antagonists**		
3	L-701,324	0.9083
10	Sinapic Acid, Methyl Ester	0.8571
11	Dizocilpine	0.8492
15	L-701,324	0.8457
19	L-701,324	0.8258
45	(-)-Mk801	0.7117
47	7-Chlorokynurenic Acid	0.7108

Table S3. Anti-inflammatory compounds with behavioral fingerprints that co-cluster with *pitpnc1a*$^{-/-}$ larvae, related to Figure 3. The compounds are organized by functional class, in order of rank (1=most closely correlated with *pitpnc1a* $^{-/-}$). Weighted Pearson correlation coefficients are also listed.

Extended Methods

Antibodies

A custom rabbit polyclonal antibody was raised to a specific peptide from zebrafish Pitpnc1a called PAb:520; the peptide sequence used for immunisation was LPSLFSWGSSSPQPE (Eurogentec). A rabbit polyclonal antibody raised against a specific peptide from human PITPNC1 called RB59 was a kind gift from S.F.Tavazoie. RB59 was raised to the C-terminus of human PITPNC1-sp1 with a sequence of DPEKKATLNLPGMHSSDK. Antibodies against PITPα (PAb:674) and PITPβ (MAb 4A7) were raised in house and have been described elsewhere (Carvou et al., 2010). Antibodies against the FLAG-tag and pan 14-3-3 were obtained commercially from Origene (TA50011) and Santa Cruz Biotechnology (sc-629), respectively. Antibodies against phospho-IGF1 receptor beta (Y1135) (#3918) were obtained from Cell Signaling Technologies, and antibody against GAPDH was obtained from Thermo (MA5-15738).

Identification and alignment of zebrafish PITPNC1 orthologs

A BLASTP search was carried out within the *Danio rerio* protein database (Refseq, NCBI) using the amino acid sequence of human PITPNC1-sp1 as a query (NP_036549.2). The obtained sequences of the zebrafish orthologues were truncated, therefore further EST analysis was then performed to obtain the full-length protein coding sequences by aligning up and downstream EST sequences.

Isolation of zebrafish cDNA

Total RNA was prepared from 24 hpf embryos using the RNeasy Plus Mini Kit (Qiagen). Around 30 embryos were homogenised in 350 µl of Buffer RLT plus and frozen at -80ºC until ready for purification. The lysate was then thawed and centrifuged at 13,000 rpm for 3 min to remove any debris and pigmentation. The resulting supernatant was subjected to spin column purification following the manufacturer’s protocol. Total RNA was then used for a reverse transcriptase reaction using Superscript II (Life Technologies) according to the manufacturer’s instructions.

PCR

RT-PCR against regions of *pitpnc1a (*LOC563621) and *pitpnc1b* (ENSDARG00000022807) was carried out using our zebrafish cDNA library as a template. PCR reactions were completed using Platinum Taq Polymerase (Life Technologies). The resulting PCR products were analysed by agarose gel electrophoresis and then purified using a QIAquick PCR purification kit (Qiagen). The PCR products were cloned using the pGEM-T easy vector system (Promega) and their sequence was verified by Sanger sequencing (MWG Eurofins).

pitpnc1a Forward Primer 5’ - CGGATTCCAGGAGTCATTTC

pitpnc1a Reverse Primer 5' - TGTCTGTGACCCAAGAGCAG

pitpnc1b Forward Primer 5' - TCCTACCGAGGTTCCATGTC

pitpnc1b Reverse Primer 5' - ATTGAATGGTTTCGCTCCAG

Cloning of the complete coding sequence with an additional FLAG-tag

RT-PCR was carried out on our zebrafish cDNA library to amplify the complete coding sequences of *pitpnc1a* and *pitpnc1b* using PfuTurbo Hot Start (Agilent Technologies) according to the manufacturer's instructions. An N-terminal FLAG epitope tag (DYKDDDDK) was engineered during the PCR reaction. The resulting PCR products were analysed by gel electrophoresis and then restriction digested with BamHI and HindIII (Promega). The digested product was ligated into the mammalian expression vector PCDNA3.1. The sequence of the resulting vector was verified by Sanger sequencing (MWG Eurofins).

FLAG-Pitpnc1a_Dr Forward Primer 5' - GACGGATCCCCACCATGGATTACAAGGATGACGACGATAAGGCAGTGTTGATGAAGGAATACCGGATATG

FLAG- Pitpnc1a_Dr Reverse Primer 5' - CTAAAGCTTTTATTACTCGGGCTGCGGGCTGCTG

FLAG- Pitpnc1b_Dr Forward Primer 5' - GACGGATCCCCACCATGGATTACAAGGATGACGACGATAAGGCAGTGTTGGTCAAAGAGTACCGGATATGCATGCCGCTC

FLAG- Pitpnc1b_Dr Reverse Primer 5' - CTAAAGCTTTTATTATTCTGGGGTTGATTTTAATCTCACAGGACCCTTG

Sub cloning with 6XHis-tag

The coding sequence of *pitpnc1a* was sub cloned by PCR from the FLAG-Pitpnc1a_Dr vector using PfuTurbo Hot Start (Agilent Technologies) according to the manufacturer's instructions. The N-terminal FLAG epitope tag (DYKDDDDK) was replaced with a 6xHis epitope tag during the PCR reaction. The resulting PCR products were analysed by gel electrophoresis and then restriction digested with BamHI and HindIII (Promega). The digested product was ligated into the mammalian expression vector PCDNA3.1. The sequence of the resulting vector was verified by Sanger sequencing (MWG Eurofins).

6xHis-Pitpnc1a_Dr Forward Primer 5' - GACGGATCCCCACCATGCATCATCATCATCATCATGCAGCAGTGTTGATGAAGGAATACCGGATATG

6xHis-Pitpnc1a_Dr Reverse Primer 5' - CTAAAGCTTTTATTACTCGGGCTGCGGGCTGCTG

Protein purification

293F cells were grown to a density of 1.0-1.4 x 10^6 cells/mL in a volume of 1 L. The cells were transfected with plasmid DNA using polyethylenenimine (PEI). To prepare the transfection mix 40 mL of OptiPro was mixed with L-glutamine to a final concentration of 4 mM. The resulting mixture was subsequently filter sterilised through a 0.22 µm filter. 1.25 mg of plasmid DNA was then added along with 1.875 mg of PEI and this mixture was allowed to complex at room temperature for 10 min. The PEI:DNA complex was added to the cells and the protein was harvested after 3-4 days of culture. To purify the protein cells were

harvested by centrifugation at 3000 rpm for 15 min at 4°C. The cell pellet was then re-suspended in 20 mL of ice-cold lysis buffer (25 mM Tris/HCl pH 7.4, 250 mM NaCl, 20 mM Imidazole, 10 mM Benzamidine) that was supplemented with protease inhibitor cocktail I and II (Sigma Aldrich). The cells were then lysed by sonication (on ice) and the resulting lysate was cleared by centrifugation at 18000 rpm for 2 hr at 4°C. The supernatant was added to a HIS-Select Nickel Affinity gel as described previously (Fensome et al., 1996). The recombinant proteins were desalted into Pipes buffer (20 mM Pipes, 137 mM NaCl and 3 mM KCl, pH 6.8) and analysed by SDS/PAGE for purity. The protein concentration was adjusted accordingly and the proteins were stored at −80ºC. From a 1 L prep, only 100 µg of protein was obtained.

Lipid transfer assays

Phosphatidylinositol (PI) transfer activity was assayed by measuring the transfer of [^{3}H]-PI from radiolabelled rat liver microsomes to unlabelled synthetic liposomes [Phosphatidylcholine (PC)/PI molar ration of 98:2] by recombinant Pitpnc1a_Dr (250 µL, 10 µg/µL), as described previously (Thomas et al., 1993). Phosphatidic acid (PA) transfer activity was assayed by measuring the transfer of [^{3}H]-PA from liposomes to unlabelled rat liver mitochondrial preparations by recombinant Pitpnc1a_Dr (250 µL, 10 µg/µL) (Yadav et al., 2015). Transfer activity was calculated as a percentage of the total radioactivity present in the assay after subtraction of the number of counts transferred in the absence of a recombinant source. Transfer activity was monitored in duplicate samples.

Electroporation of Cos-7 cells

Cos-7 cells were transfected with either pcDNA3.1 or pcDNA3.1-FlagPITPNC1 by electroporation. The cells were trypsinised and mixed with 10 µg of the respective construct in a sterile electroporation cuvette. For electroporation two pulses of 0.220 kV and 950 µF were delivered and the cells were transferred directly to ice for 5 min. Cells were then transferred to a tissue culture flask and returned to the incubator (37°C, 5% CO_2). Cells were ready for experimental use 48 hr post transfection.

Immunoprecipitations

Cells were harvested by trypsinisation and were washed with ice cold PBS. The cells were re-suspended in PIPES buffer (20 mM Pipes, 137 mM NaCl and 3 mM KCl, pH 6.8) supplemented with protease and phosphatase I and II inhibitors (Sigma–Aldrich, P8340, P2850 and P5726 respectively). The cells were sonicated and the membrane fraction was pelleted by centrifugation (50,000 rpm for 1 hr at 4°C), the cytosol fraction (supernatant) was retained for further analysis. The BCA (bicinchoninic acid) assay was used to determine the protein concentration of the cytosol fraction. For immunoprecipitation, 1200 µg of cytosolic protein was incubated with 50 µL of equilibrated anti-FLAG M2 affinity gel (Sigma–Aldrich, A2220) on a rotating wheel for 1 hr at 4°C. The tubes were centrifuged at 10,000 rpm for 2 min (4°C) and the supernatant was discarded. The beads were washed four times with PIPES buffer; the final wash was carried out in a fresh Eppendorf tube. After the final centrifugation, the supernatant was aspirated and replaced with 30 µL of NuPAGE LDS sample buffer under reducing conditions (Life Technologies).

Cos-7 protein preparation

The Cos-7 cell monolayer was washed with ice cold PBS and the cells were then harvested with RIPA buffer (50 mM Tris/HCl pH 8, 150 mM NaCl, 1% Triton-X 100, 0.5% Sodium Deoxycholate, 0.1% SDS) supplemented with protease inhibitors (Sigma Aldrich) on ice. The lysates were briefly sonicated and then cleared by centrifugation at 15,000 rpm, 4°C. The BCA assay was used to determine the protein concentration of the samples.

Zebrafish tissue protein preparation

Zebrafish tissues were homogenised with RIPA buffer (50 mM Tris/HCl pH 8, 150 mM NaCl, 1% Triton-X 100, 0.5% Sodium Deoxycholate, 0.1% SDS) supplemented with protease inhibitors (Sigma Aldrich) on ice. The homogenates were briefly sonicated and then cleared by centrifugation at 15,000 rpm, 4°C. The BCA assay was used to determine the protein concentration of the samples.

Cytosol protein preparation from rat tissues

Rat tissues were homogenized in PIPES buffer (20 mM PIPES, 137 mM NaCl and 3 mM KCl, pH 6.8) in the presence of a protease inhibitor cocktail (Sigma). To prepare membranes and cytosol, the lysates were centrifuged for 10 minutes (15,000 rpm, 4°C) to pellet the nuclei and unbroken cells. The lysate was then centrifuged for 1 hour at 50,000 rpm, 4°C (rotor type TLA55, Beckman Optima benchtop ULTRA) to pellet the membranes, and the supernatant consisting of the cytosolic fraction was retained for further analysis.

HEK293 standard preparation

1×10^6 HEK293 cells were seeded into a 10 cm dish and allowed to adhere overnight (37°C, 5% CO_2). The cells were then transfected using FugeneHD (Promega) with FLAG-Pitpnc1a according to the manufacturer’s instructions. The cells were ready for experimental use 24 hr after transfection.

Western blot analysis

Protein samples were prepared in NuPAGE LDS Sample Buffer under reducing conditions (Life Technologies) and were boiled prior to loading on the gel (70°C, 5 min). SDS-PAGE was carried out using a Novex NuPAGE gel electrophoresis system (Life Technologies). Within this system the samples were analysed on NuPAGE 4-12% Bis-Tris gels (1.5 mm thick, 10-well) according to the manufacturer’s instructions. After SDS-PAGE the proteins were transferred to an Immobilin-P PVDF membrane (Millipore) for blotting. The following primary antibody dilutions were used; PAb:520 = 1:100; GAPDH = 1:3000; FLAG = 1:2000; 14-3-3 = 1,1000; PAb:647 = 1:1000; MAb:4A7 = 1:1000; RB59 = 1:50.

Whole-mount in situ hybridisation (colorimetric)

Digoxigenin (DIG)-labelled sense and antisense riboprobes corresponding to regions of *pitpnc1a*, *pitpnc1b*, *npvf*, and *galanin* mRNA were synthesized using T7 and SP6 RNA polymerases (Roche) using linearized

PGEMT vectors as a template. DIG-labelled anti-sense probes for *egr3*, *tbr1a*, *eomesa* and *fosab* were a kind gift from the Steve Wilson Group (UCL).

Prior to in situ hybridization, dechorionated embryos were fixed at the appropriate stage using 4% Paraformaldehyde (PFA) in PBS overnight at 4°C. The following day the embryos were rinsed into PBST and bleached (3% H_2O_2, 1% KOH) for 10-15 minutes at room temperature. Adult brains were dissected and fixed using 4% PFA in PBS overnight at 4°C. The samples were then dehydrated through a PBST:methanol series (25%, 50%, 75%, 100%) each step taking 5 minutes at room temperature. Embryos were stored in 100% methanol at -20°C at least for overnight and until ready for use.

Embryos that were prepared for brain dissection were fixed with 4% PFA and 4% Sucrose in PBS overnight at 4°C, followed by the brain dissection with sharp forceps and methanol dehydration and storage (as above).

Embryos or dissected brains were then rehydrated back through a methanol:PBST series (25%, 50%, 75%, 100%) each step taking 5 minutes at room temperature. The samples were then subjected to a proteinase K treatment followed by a post fixation for 20 minutes at room temperature in 4% PFA in PBS.

Samples were treated in pre-hybridisation buffer (50% formamide, 5X SSC, 0.1mg/mL tRNA, 0.01 mg/mL heparin, 9.2 mM citric acid, 0.1% Tween 20) at 67°C for at least 1 hour. Probes were then added and the samples were incubated at 67°C overnight. The samples were then subjected to washes to remove excess unbound probes. The hybridisations were developed using nitroblue tetrazolium (NBT)/5-bromo-4-chloro-3-indolyl phosphate (BCIP) substrate (Roche). Whole-mount specimens were mounted in 80% glycerol and photographed with a Nikon SMZ1500 microscope mounted with a Leica MC190HD camera.

Immunohistochemistry

Wild type and *pitpnc1a*$^{-/-}$ larvae were fixed overnight at 4ºC in 4% PFA, washed 2 times for 5 min in PBST, then dehydrated through a MeOH series (25%, 50%, 75%, 100%) and stored at -80ºC. Larvae were rehydrated in PBST and the brains were exposed by dissection of the skin and head with forceps. Mutant and wild type larvae were then placed in the same tubes for all subsequent steps, with the mutants distinguished from wild type by cutting the tail. Larvae were washed 3X in PBS then blocked for 2 hr in 10% goat serum, 1%DMSO, PBT, then incubated overnight at 4ºC with anti-pIGFR beta (Y1135 DA7A8) diluted 1:500 in blocking solution. The next day, larvae were washed 6X in PBT for 15 min, then incubated overnight at 4ºC with goat anti-rabbit Alexa 488 (A11034, Life Technologies) at 1:200. Larvae were then washed 6X in PBT before mounting in 60% glycerol for imaging with a confocal microscope.

Supplemental References

Carvou, N., Holic, R., Li, M., Futter, C., Skippen, A., and Cockcroft, S. (2010). Phosphatidylinositol- and phosphatidylcholine-transfer activity of PITPbeta is essential for COPI-mediated retrograde transport from the Golgi to the endoplasmic reticulum. J. Cell Sci. *123*, 1262–1273.

Fensome, A., Cunningham, E., Prosser, S., Tan, S.K., Swigart, P., Thomas, G., Hsuan, J., and Cockcroft, S. (1996). ARF and PITP restore GTP gamma S-stimulated protein secretion from cytosol-depleted HL60 cells by promoting PIP2 synthesis. Curr. Biol. CB *6*, 730–738.

Thomas, G.M.H., Cunningham, E., Fensome, A., Ball, A., Totty, N.F., Troung, O., Hsuan, J.J.C., and S. (1993). An essential role for phosphatidylinositol transfer protein in phospholipase C-mediated inositol lipid signalling. Cell *74*, 919–928.
